# Greater traditionalism predicts COVID-19 precautionary behaviors across 27 societies

**DOI:** 10.1038/s41598-023-29655-0

**Published:** 2023-04-11

**Authors:** Theodore Samore, Daniel M. T. Fessler, Adam Maxwell Sparks, Colin Holbrook, Lene Aarøe, Carmen Gloria Baeza, María Teresa Barbato, Pat Barclay, Renatas Berniūnas, Jorge Contreras-Garduño, Bernardo Costa-Neves, Maria del Pilar Grazioso, Pınar Elmas, Peter Fedor, Ana Maria Fernandez, Regina Fernández-Morales, Leonel Garcia-Marques, Paulina Giraldo-Perez, Pelin Gul, Fanny Habacht, Youssef Hasan, Earl John Hernandez, Tomasz Jarmakowski, Shanmukh Kamble, Tatsuya Kameda, Bia Kim, Tom R. Kupfer, Maho Kurita, Norman P. Li, Junsong Lu, Francesca R. Luberti, María Andrée Maegli, Marinés Mejia, Coby Morvinski, Aoi Naito, Alice Ng’ang’a, Angélica Nascimento de Oliveira, Daniel N. Posner, Pavol Prokop, Yaniv Shani, Walter Omar Paniagua Solorzano, Stefan Stieger, Angela Oktavia Suryani, Lynn K. L. Tan, Joshua M. Tybur, Hugo Viciana, Amandine Visine, Jin Wang, Xiao-Tian Wang

**Affiliations:** 1 Department of Anthropology, Center for Behavior, Evolution, and Culture, University of California, Los Angeles, CA 90095 USA; 2 Department of Anthropology, Center for Behavior, Evolution, and Culture, Bedari Kindness Institute, University of California, Los Angeles, CA 90095 USA; 3 Department of Cognitive and Information Sciences, University of California, Merced, CA 95343 USA; 4 Department of Political Science, Aarhus University, 8000 Aarhus C, Denmark; 5 Laboratorio de Evolución y Relaciones Interpersonales, Universidad de Santiago de Chile, Santiago, Chile; 6 Department of Psychology, University of Guelph, Guelph, ON N1G 2W1 Canada; 7 Institute of Psychology, Vilnius University, Vilnius, Lithuania; 8 Escuela Nacional de Estudio Superiores, Universidad Nacional Autónoma de México, Unidad Morelia, 58190 Morelia, Michoacán Mexico; 9 Lisbon Medical School, University of Lisbon, 1649-028 Lisbon, Portugal; 10 Centro Hospitalar Psiquiátrico de Lisboa, 1749-002 Lisbon, Portugal; 11 Centro Integral de Psicología Aplicada, Universidad del Valle de Guatemala, Guatemala City, 01015 Guatemala; 12 Proyecto Aiglé Guatemala, Guatemala City, Guatemala; 13 Department of Psychology, Adnan Menderes University, Aydın, Turkey; 14 Department of Environmental Ecology and Landscape Management, Faculty of Natural Sciences, Comenius University, 842 15 Bratislava, Slovakia; 15 Facultad de Humanidades, Universidad Rafael Landivár, Guatemala City, 01016 Guatemala; 16 Departamento de Psicología, Universidad Francisco Marroquín, Guatemala City, Guatemala; 17 CICPsi Research Center for Psychological Science, Universidade de Lisboa, Lisbon, Portugal; 18 School of Psychology, Universidade de Lisboa, Lisbon, Portugal; 19 The School of Biological Sciences, The University of Auckland, Auckland, 1010 New Zealand; 20 Department of Sustainable Health, University of Groningen, Campus Fryslân, Groningen, The Netherlands; 21 Division of Psychological Methodology, Department of Psychology and Psychodynamics, Karl Landsteiner University of Health Sciences, Krems an der Donau, Austria; 22 Psychology Program, Department of Social Sciences, College of Arts and Sciences, Qatar University, 2713 Doha, Qatar; 23 College of Arts and Sciences, Partido State University, Goa, 4422 Camarines Sur Philippines; 24 Institute of Psychology, Nicolaus Copernicus University in Toruń, 87-100 Toruń, Poland; 25 Department of Psychology, Karnatak University, Dharwad, Karnataka 580003 India; 26 Department of Social Psychology, The University of Tokyo, Tokyo, 113-0033 Japan; 27 Brain Science Institute, Tamagawa University, Tokyo, 194-8610 Japan; 28 Center for Experimental Research in Social Sciences, Hokkaido University, Sapporo, 060-0810 Japan; 29 Department of Psychology, Pusan National University, Busan, South Korea; 30 Department of Psychology, Nottingham Trent University, Nottingham, NG1 4FQ UK; 31 School of Social Sciences, Singapore Management University, Singapore, 188065 Singapore; 32 School of Humanities and Social Science, The Chinese University of Hong Kong (Shenzhen), Shenzhen, 518172 China; 33 Department of Psychology, Nipissing University, North Bay, ON P1B 8L7 Canada; 34 Department of Psychology, Universidad del Valle de Guatemala, Guatemala City, 01015 Guatemala; 35 Department of Management, Ben-Gurion University of the Negev, Be’er Sheva, Israel; 36 Japan Society for the Promotion of Science, Tokyo, 102-0083 Japan; 37 Lazaridis School of Business and Economics, Wilfrid Laurier University, Waterloo, ON N2L 3C5 Canada; 38 Department of Political Science, University of California, Los Angeles, CA 90095 USA; 39 Institute of Zoology, Slovak Academy of Sciences, 845 06 Bratislava, Slovakia; 40 Coller School of Management, Tel Aviv University, Tel Aviv, Israel; 41 Atma Jaya Catholic University of Indonesia, Jakarta, 12930 Indonesia; 42 Department of Experimental and Applied Psychology, Vrije Universiteit Amsterdam, Amsterdam, 1081 HV The Netherlands; 43 Departamento de Filosofía y Lógica y Filosofía de la Ciencia, Universidad de Sevilla, 41018 Seville, Spain; 44 L’Institut Agro Montpellier, 34060 Montpellier, France

**Keywords:** Psychology, Human behaviour

## Abstract

People vary both in their embrace of their society’s traditions, and in their perception of hazards as salient and necessitating a response. Over evolutionary time, traditions have offered avenues for addressing hazards, plausibly resulting in linkages between orientations toward tradition and orientations toward danger. Emerging research documents connections between traditionalism and threat responsivity, including pathogen-avoidance motivations. Additionally, because hazard-mitigating behaviors can conflict with competing priorities, associations between traditionalism and pathogen avoidance may hinge on contextually contingent tradeoffs. The COVID-19 pandemic provides a real-world test of the posited relationship between traditionalism and hazard avoidance. Across 27 societies (*N* = 7844), we find that, in a majority of countries, individuals’ endorsement of tradition positively correlates with their adherence to costly COVID-19-avoidance behaviors; accounting for some of the conflicts that arise between public health precautions and other objectives further strengthens this evidence that traditionalism is associated with greater attention to hazards.

## Introduction

Traditionalism—the tendency to embrace what are perceived to be the longstanding norms and values of one’s group, while rejecting changes to them—varies across individuals^1^. Given the centrality of sociality and culture for humans, individuals’ orientations toward traditions have important downstream consequences. These include the tendency to embrace or reject innovations in the face of environmental change^2^, the ability to coordinate actions with fellow group members^3^, and the shaping of political attitudes and ideologies in democratic contexts^4^. It is therefore vital to understand factors that contribute to variation in traditionalism.

Emerging research demonstrates associations between individual differences in traditionalism and variation in the propensity to attend, and respond, to hazards^3,5^. Initial evidence indicates that individual variation in traditionalism may in part associate with variation in pathogen avoidance, the motivation to take actions to alleviate the costs of potential pathogen threats^3,6–10^. Hence, what is termed the *traditional norms* account^7^ identifies pathogen avoidance as an important factor relating to traditionalism. Consistent with the traditional norms account, multiple evolutionary pathways may lead individuals to leverage adherence to tradition as a way of ameliorating danger^3,7^.

First, as a result of cultural evolutionary processes favoring beliefs and practices that benefit individual and group fitness^11^, some traditions may have instrumental value for addressing particular pathogen threats^12^. While it is possible that individuals explicitly or implicitly understand the connections between some instrumental norms and their outcomes, the functionality of norms is frequently opaque to those who adopt them^13,14^. If the average instrumental benefit of adhering to traditions when confronting danger outweighs the costs of imprecision resulting from causal opacity, then individuals may be motivated to broadly embrace traditions in pursuit of safety. Co-evolution may have resulted in psychological adaptations or reaction norms connecting traditions to threat if the above cost–benefit structure was common over evolutionary time.

The benefits of sociality generate a second pathway by which an association between traditionalism and the salience of pathogen threats could arise. Adherence to traditional norms might provide broad payoffs via increased social support, for example by signaling in-group identity in cooperative exchanges and systems of indirect reciprocity, and/or by facilitating in-group coordination^15–17^. Such benefits might plausibly include cost amelioration in the face of pathogen threats, for example by obtaining care and resources during periods of illness^18^.

For all of the above possibilities, natural selection could have produced either (a) stable dispositional linkages between pathogen-threat concerns and long-term preferences for tradition, (b) facultative plasticity, such that individuals prophylactically upregulate their embrace of tradition in response to cues indicating an increased risk of disease, or (c) both. Together, these considerations generate the prediction that, ceteris paribus, relative to individuals less invested in tradition, those who evince greater traditionalism will be more inclined to attempt to diminish the risk of acquiring transmissible disease.

Note that the theorized connection between traditionalism and threat avoidance mirrors a similar putative relationship between social conservatism and threat avoidance, where socially conservative beliefs reflect support for tradition^see^^19,20^ in contexts where people hold political ideologies. Indeed, much of the theoretical work connecting traditional attitudes with threat reactivity comes out of political psychology, where extensive prior research has long recognized the role that motives to mitigate uncertainty, fear, and threat—particularly disease threats and threats to the stability of the social system—play in shaping socially conservative ideology^19,21–23^.

In the present research, our focus is on traditionalism writ large rather than social conservatism in particular. Political ideologies are culturally relevant in some contexts but not others. In contrast, by virtue of their translatability across cultural and political contexts, attitudinal antecedents such as traditionalism are better suited for large-scale cross-cultural investigation. That said, the underlying evolutionary logic presented here draws on, and is consistent with, seminal theoretical perspectives in political psychology that identify the existential motivations (compare to the proximate motivation to reduce threat), epistemic motivations (compare to the potential instrumental value of traditionalism/conservatism in reducing threat), and relational motivations (compare to the potential sociality benefit of traditionalism/conservatism in reducing threat) that underly political ideologies^24^.

Although adherence to tradition can provide benefits, it can also entail costs. In addition to political considerations, there are often tangible costs to sticking to the tried-and-true—most notably because innovations may generate higher payoffs than existing practices. Any given manifestation of a linkage between threat-mitigating behavior and traditionalism may therefore depend in part on how individuals assign weights to the cost–benefit structure characterizing the specific context, and exceptions to that connection should be expected when competing priorities arise. Moreover, behaviors that mitigate the costs of a threat may lead to costs in other areas, either directly, or indirectly due to the zero-sum nature of the time, attention, and resources available. Taken in sum, the relationship between traditionalism and pathogen avoidance may not be straightforward if responses to pathogen threats are perceived to clash with other priorities.

Much of the previous literature on the relationship between traditionalism and pathogen avoidance does not take account of the costs of the latter. Investigators often rely on subjective responses to hypothetical scenarios^(e.g.^^25,26^^)^—for example, feeling sick after witnessing someone vomit—that do not distinguish the real-world contexts, conflicting goals, or costs of the relevant behaviors (such as opportunity costs, allocation tradeoffs between—or vulnerabilities to—different threats, etc.). Using hypothetical scenarios is sensible in research that aims to measure emotional and/or behavioral tendencies—which may correlate with general behavioral tendencies^27^—while holding contextual factors equal. However, hypotheticals cannot capture the specific tradeoffs that likely determine how such propensities play out in consequential real-world decision making.

Past research predominantly employs samples from a narrow range of societies. Given that cost–benefit structures are likely culturally variant, the observed associations between traditionalism and pathogen avoidance may be rooted in aspects of particular practices, values, or beliefs within those societies. Hence, at present, the extent to which traditionalism and threat-avoidance behaviors are related across the highly variable traditional practices and beliefs of diverse societies is not fully known.

Encouragingly, research has begun to take the costs of pathogen avoidance into account^10,28,29^. Likewise, though relying on hypothetical scenarios, a recent study examined the relationship between disgust sensitivity and traditionalism in a large cross-cultural sample^7^. However, to date, no large-scale international investigation has addressed the relationship between pathogen avoidance and traditionalism in a real-world context, or assessed the potential for conflicts between pathogen avoidance and competing goals to impact said relationship. The COVID-19 pandemic affords such research.

The pandemic involves a pathogen threat that is both salient for much of the world’s population^30^ and has had marked effects on behavior^31^. Further, these real-world behaviors are inherently costly^32^, and may epitomize the kinds of cost–benefit tradeoffs individuals face when various priorities are perceived to clash. Moreover, individuals are influenced by their information environments, which can in turn shape perceptions of costs and benefits regardless of the actual underlying distribution. Concordantly, from an error management perspective^33^, individuals must balance the relative costs and frequencies of type 1 and type 2 errors when it comes to disease threats (i.e. the cost of taking insufficient precautions against a hazardous disease versus the social and opportunity costs entailed by being overly cautious). Indeed, individuals appear to be influenced by decision processes that reduce the probability of committing the more costly error in the context of disease avoidance^34,35^. In addition to the tradeoffs between disease avoidance and social opportunities, in social ecologies wherein COVID-19 precautions are positively or negatively moralized, error-management considerations will likely also include the reputational costs of locally counter-normative behavior.

The traditional norms account of the relationship between traditionalism and threat avoidance predicts that, all else equal, precautionary COVID-19 health behaviors should correlate with traditionalism, given that such behaviors can accurately index general pathogen avoidance motivations by virtue of occurring in a real-world context. Specifically, if traditionalism and pathogen avoidance motivations are linked, then the extent to which individuals engage in COVID-19 prophylaxis should correspond with the extent to which they embrace traditions.

Despite the apparent simplicity of the above prediction, all else may not be equal in the case of reactions to the current pandemic, as group-level and individual-level contextual factors may parochially shape the perceived cost–benefit structure of COVID-19 health precautions. For example, at the group level, precautions promulgated by public health authorities may be seen as threatening economic prosperity or personal liberty to a greater extent in some cultural contexts than in others. Individual assessments of those countervailing tradeoffs, shaped by the social and political environment, will likely vary as well. Furthermore, some public health precautions may directly interfere with traditional practices; for example, social distancing restrictions preclude the kinds of ritual gatherings that are often important for religious services and other activities central to in-group identity. Finally, as stated above, individuals’ characterizations of the cost–benefit structures may or may not be accurate: miscalculations or erroneous beliefs can arise. In particular, for politically, ideologically, and socially salient issues such as the pandemic, individuals’ information environments may shape inaccurate beliefs about such tradeoffs. In sum, these clashes potentially reduce, or even reverse, the observed relationship between pathogen avoidance behaviors—in this case, COVID-19 health precautions—and traditionalism.

Recent research has found support for both the traditional norms account and the presence of tradeoffs. At the national level, consistent with the logic connecting traditions and threat mitigation, researchers have found that greater cultural tightness (i.e. stronger and more heavily enforced social norms and constraints) correlated negatively with COVID-19 incidence rates^36^. At the individual level, two recent studies in the U.S^10^ found that variables such as greater economic conservatism and lower trust in scientists statistically suppressed the traditionalism-COVID-19 precautions relationship. Concordantly, consonant with the close relationship between traditionalism and social conservatism^4^, other research provides evidence for an increase in social conservatism in the U.S., Poland, and the U.K. following the start of the pandemic^8,37,38^^, but see^^39^. However, these results come from only three societies, and may be contingent on the parochial conditions obtaining therein, notably including the extensive politicization of the pandemic in the U.S. and Poland^40,41^. We therefore investigated the relationship between COVID-19 precautions and traditionalism across 27 countries, examining both the zero-order relationships and the direct relationships after statistically accounting for indirect effects (i.e., mediation or suppression) of variables related to the perception that COVID-19 precautions exacerbate other threats or otherwise conflict with competing priorities.

## Research questions

### Do COVID-19 health precautions, as potential manifestations of general pathogen avoidance tendencies, positively correlate with traditionalism across diverse societies?

Our primary goal was to assess whether the hypothesis that traditionalism and pathogen avoidance covary at the individual level obtains across a wide array of cultural contexts. Specifically, we were interested in whether individuals’ choices to adopt precautionary COVID-19 behaviors positively associated with their own endorsement of traditionalism. We used individuals’ self-reports of their actual COVID-19 precautionary health behaviors (such as mask wearing, social distancing, and supplement taking) as a complex, real-world manifestation of pathogen avoidance behavior. We selected precautionary behaviors that had been widely adopted across the globe, and that had been plausibly viewed as medically- or public health-derived preventative measures by experts and/or laypeople. The actual efficacy of the precautions in question varied. In contrast to previous methods that left the costs of pathogen avoidance unspecified, individuals’ decisions about COVID-19 precautions intrinsically embody the kinds of tradeoff calculations discussed above.

Because specific traditions and cultural practices vary substantially across societies, to measure traditionalism, we examined individuals’ general tendency to endorse or reject the traditional norms and values of their society writ large, rather than the specific content of those traditions themselves. This allowed us to measure traditionalism in a relatively consistent manner across study sites, affording comparisons despite wide variation in the contents of traditions.

Testing the individual-level relationship between traditionalism and COVID-19 precautions across many cultural contexts was important for at least two reasons. First, given claims of an evolved link between traditionalism and general pathogen avoidance, it is critical to determine whether that relationship is evident across a broad swath of humanity. Second, given that clashes between pathogen avoidance and other priorities are likely often parochial as a function of different cultural values and beliefs, examining the individual-level traditionalism-pathogen avoidance relationship across many societies affords identification of overarching patterns despite local variation.

### Do perceived tradeoffs between health precautions and other priorities influence the traditionalism-precautions relationship?

Parochial factors interacting with individual preferences may conceal direct relationships between pathogen avoidance and traditionalism. For example, a recent study found evidence that, in the U.S., greater economic conservatism, greater social dominance orientation (SDO), and lower trust in science statistically suppressed the direct precautions-traditionalism relationship^10^. Consistent with the importance of tradeoffs in shaping the relationship between pathogen avoidance and traditionalism, we expect such suppression to occur when competing priorities that also associate with traditionalism, such as personal liberties, are perceived to clash with COVID-19 precautions.

It is an open question whether, in other societies, individuals similarly weight the components of the cost–benefit tradeoffs previously identified in the U.S. On the one hand, many aspects of the U.S.’ socio-political environment are unlikely to generalize beyond its borders. On the other hand, pathogen avoidance precautions—particularly in the case of COVID-19—may commonly be perceived to clash with benefits derived from social interaction, including both economic and community activity. Therefore, in the present study, we also sought to investigate the extent to which the suppressive dynamics identified in previous research in the U.S.^10^ emerge across a much broader range of socio-political contexts.

Drawing on the previous research conducted in the U.S., we tested seven theoretically relevant variables that may suppress traditionalism-precautions relationships in some cultural contexts. First, we measured concerns over personal liberties and the economy, as well as perceived tradeoffs between personal liberties, the economy, and the practice of traditions on the one hand, and COVID-19 public health precautions on the other. Here, we explicitly pitted public health precautions against priorities that have been commonly perceived to clash in some societal contexts. Second, we measured trust in scientists regarding COVID-19 information. Because many scientific explanations for natural phenomena are incompatible with many traditional explanations thereof, trust in scientists may negatively correlate with traditionalism in many cultural contexts. If this is the case, and if COVID-19 public health precautions are perceived to derive from the advice of scientists, traditionalists may discount these precautions, resulting in suppression of any direct positive relationships between traditionalism and COVID-19 precautions. The precise configuration, however, will depend on culturally parochial relationships between traditional and scientific meaning systems.

Finally, related to the logic regarding trust in scientists, we included a measure of SDO. SDO contributes to distrust in both scientists and various scientific findings, likely because scientists are more likely to be viewed as actors seeking to disrupt the social hierarchies preferred by individuals with higher SDO^42^. This may be particularly true when hierarchy-promoting authoritarian leaders denounce the legitimacy of scientists in the context of COVID-19, or imply that their recommended practices are only for the weak. Likewise, SDO may reflect preferences for fewer constraints on individual liberties regardless of their effects on public goods^43^. Because traditionalism also intersects with preferences for authoritarian leaders^1^, and associates with SDO in some socio-political contexts^44,45^, SDO might act as a statistical suppressor of any direct relationship between traditionalism and COVID-19 precautions when the above conditions are met.

We did not make specific predictions about the effects of each of the above variables at each of the study sites, and we did not expect to find suppression across all countries given the likelihood that many of these tradeoff dynamics are parochial. Further, this was not an exhaustive test of every possible dynamic that may be relevant to the zero-order relationship across individual societies. Rather, we sought to explore the generalizability of the extent to which the particular factors operating in the U.S. also exert suppressive effects elsewhere, perhaps reflective of some relatively common ways in which pathogen avoidance behaviors can clash with competing priorities.

## Results

### Baseline relationships between COVID-19 precautions and traditionalism across study sites

**Figure 1:**
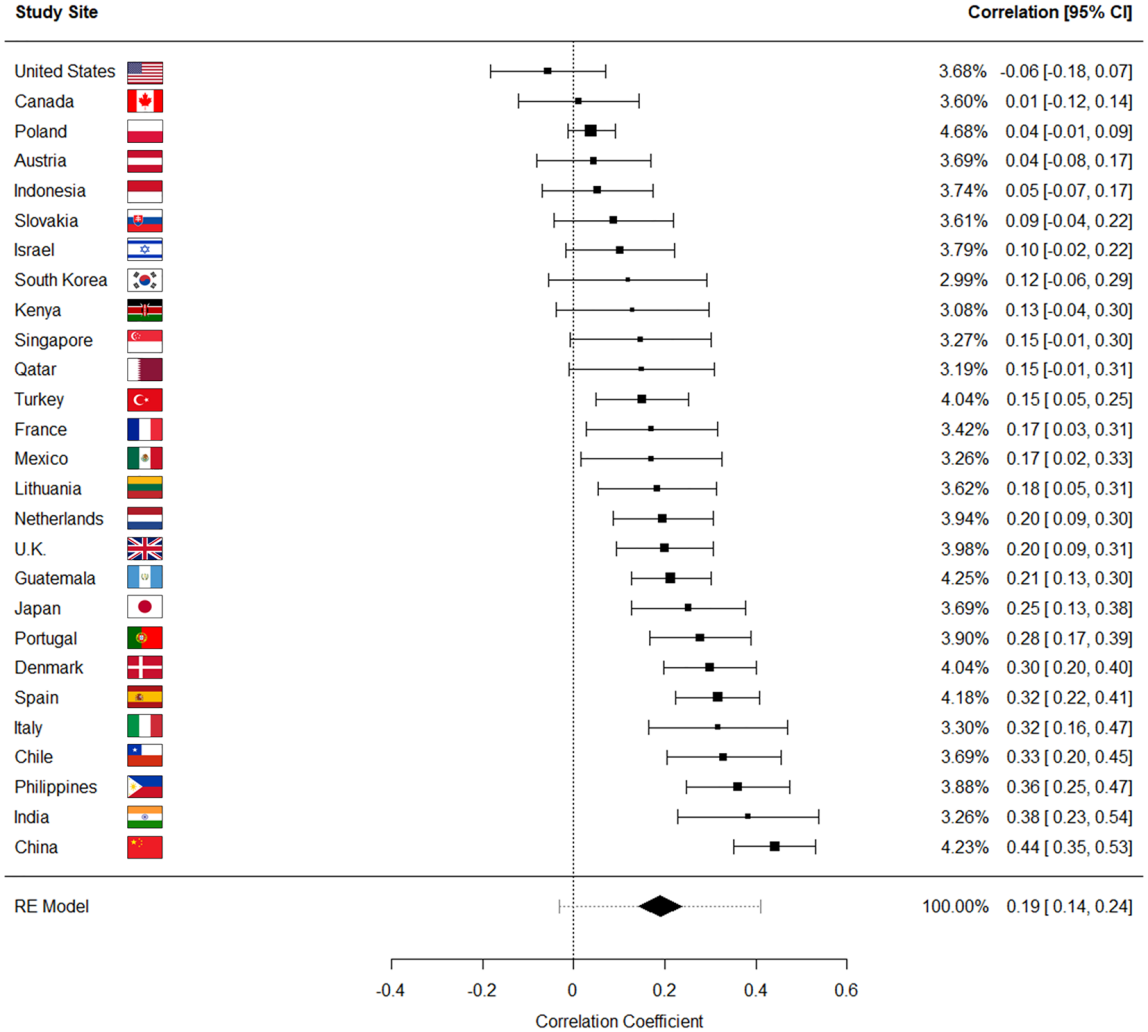
Results of a random effects, restricted maximum likelihood meta-analysis in which each study site was treated as a separate sample. Plot shows zero-order product-moment correlations between traditionalism and COVID-19 health precautions at each study site, ordered by effect size. For the individual country estimates, the location of the square along the x-axis corresponds with the correlation coefficient, the size of the square corresponds with the weight of that study site in the meta-analysis, and bands are 95% confidence intervals. At the bottom of the plot, an overall meta-analyzed point estimate is provided. The midpoint of the diamond corresponds with that point estimate, the width of the diamond corresponds with the 95% CI, and the dotted bands correspond with the 95% prediction interval. On the right side of the plot, weights, correlation coefficients, and 95% CIs respectively are numerically listed for both the site-specific correlations, as well as the overall estimate. Note that for the overall meta-analyzed point estimate, the 95% confidence interval does not overlap with zero, while the 95% prediction interval does.

**Figure 2:**
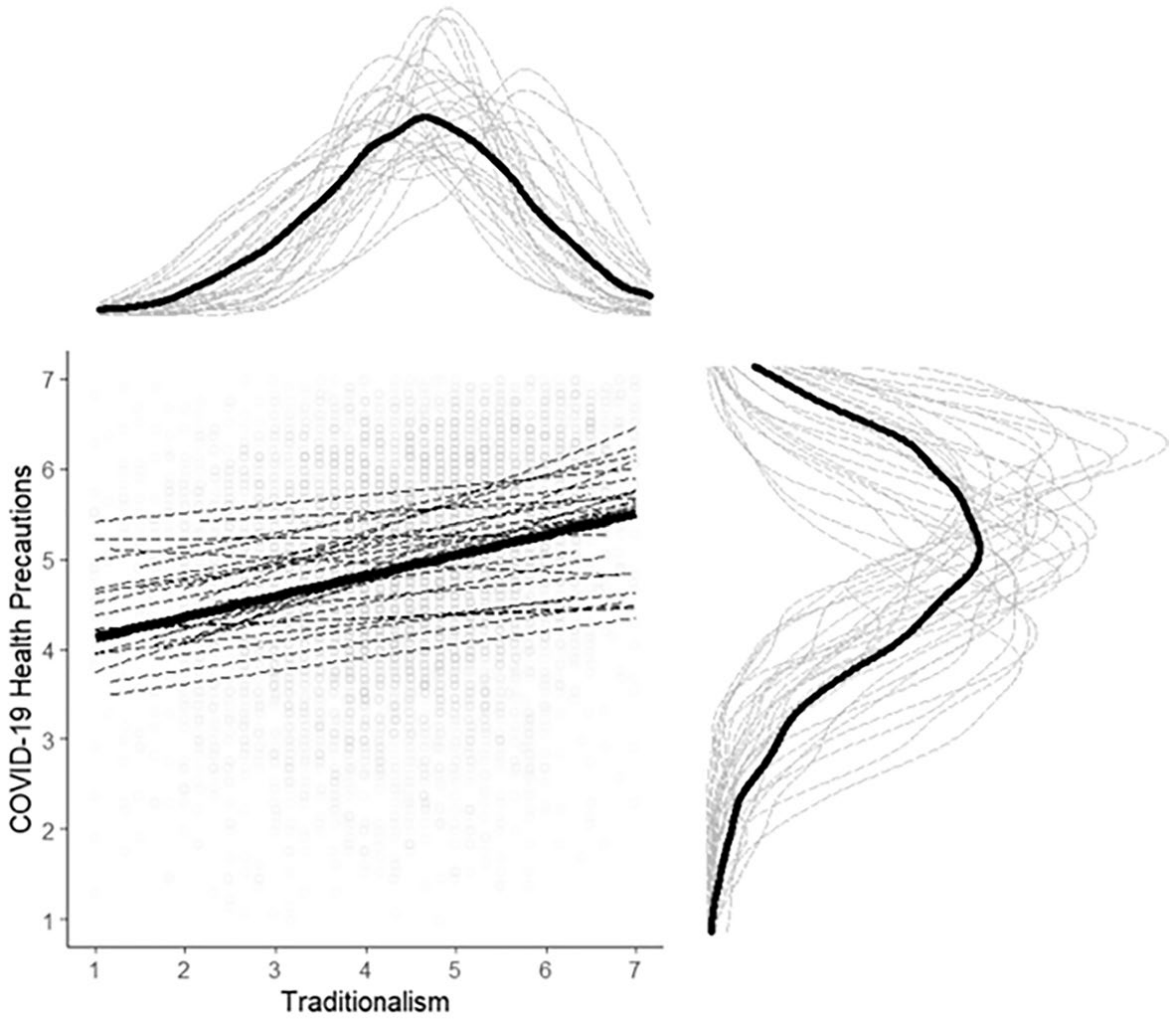
Graphical visualization of the country-specific correlations listed in Fig. 1. Dotted lines are study site-specific product-moment correlations between traditionalism and COVID-19 health precautions. The solid thick line is the unweighted product-moment correlation in the pooled sample across all study sites. Dots show individual data points, jittered along the x- and y-axes to aid interpretability. Density plots along the x- and y-axes represent the raw distributions of the traditionalism and COVID-19 health precautions composites. Thin grey lines show density distributions at individual study sites, whereas the thick black lines show the overall distribution in the pooled sample across all study sites. Study sites are unlabeled to improve readability. For labeled study-site specific correlations and density distributions, see Figs. S2–S4 in the Supplement.

Treating each study site as a separate sample, we conducted a random effects meta-analysis to test the extent to which overall indices of COVID-19 precautions and traditionalism were related across study sites (see Figs. 1 and 2). At the majority of study sites (16 of 27), the relationship between traditionalism and COVID-19 precautions was positive and significant, as was the overall meta-analyzed point estimate representing a weighted average of the effects found for each study site (*r* = 0.19, 95% confidence interval [0.14, 0.24]; note that the 95% confidence interval for the overall estimate does not overlap with zero). There was also substantial variation across study sites, as indicated by observed levels of heterogeneity (I^2^ = 78.34%; 95% prediction interval [− 0.03, 0.41]); concordantly, the 95% prediction interval overlapped with zero, suggesting that if similar nations were randomly added to the sample, some of their true effect sizes would be null, or even negative^46^.

These results were robust to the inclusion of demographic controls—including age and education—as well as COVID-19-related covariates, such as participants’ estimates of COVID-19 prevalence (see Fig. S5; see “Methods” section for details on COVID-19-related covariates). Additionally, the reliability of the traditionalism composite varied widely across study sites (*α*s 0.39–0.88, mean *α* = 0.74; see Table S8). To address this, (a) we performed item-by-item meta-analyses using each item from the traditionalism composite separately (see Supplement page S44)—results were conceptually unchanged compared to the composite, and were similar across items, with some variation in effect size—and (b) given the possibility of measurement error contributing to unreliability, we performed random effects meta-analyses using the traditionalism composite that disattenuated for unreliability^47^, see Fig. S7. These analyses used averages of raw scores to create composite indices for traditionalism and COVID-19 precautions, where item inclusion was based on the results of factor analyses (see “Methods” section and Supplement pages S30 and S38 for details). While averaged composites are easier to interpret, they may make unrealistic assumptions about the relative weights of each item in the composites. We therefore tested whether using factor scores instead of raw averages for the traditionalism and COVID-19 precautions indices conceptually altered the results. Factor scores were highly correlated with the raw average composites (marginal R2s = 0.96–0.98), and using them in place of the raw average composites did not conceptually change results (see Supplement page S62 for details). Finally, country-specific estimates of COVID-19 prevalence at the time of data collection did not explain any of the variance in effect sizes between study sites when tested in a meta-regression (see Supplement page S23), although the reliability of officially reported prevalence numbers may vary across study sites.

### Exploring the effects of potential suppressor variables:

To test the generalizability of suppression phenomena originally observed in the U.S. socio-political context, we examined the extent to which the potential suppressor variables assessed in those studies affected the zero-order precautions-traditionalism relationship across study sites. Here, suppression refers to variables that result in a negative indirect relationship between traditionalism and health precautions in a mediation analysis, such that accounting for them in a regression increases (rather than decreases, as in a traditional mediation analysis) the effect size of the direct positive traditionalism-precautions relationship^48^. We therefore conducted a second random effects meta-analysis on the traditionalism-precautions relationship accounting for the effects of potential suppressor variables.

In order to use the same set of candidate suppressors for each study site in the meta-analysis, we first identified suppressors in a pooled sample across all study sites. Using bootstrapping procedures to determine confidence intervals, we utilized mixed-effects mediation analyses with study site set as a random effect to test whether any of the seven candidate variables were suppressing the precautions-traditionalism relationship in the pooled sample. Of the seven variables, we identified five suppressors in the pooled sample (see Table S1): SDO; distrust in scientists; and perceived tradeoffs between COVID-19 public health efforts and personal liberties, the economy, and personal traditions, respectively. See Supplement pages S66–S71 for information on mean levels of each suppressor variable across study sites.

Next, we assessed the combined effects of all five suppressors at each study site (see Table S2). We observed a wide range of indirect effects across study sites, ranging from suppression in slightly less than half of the study sites, all the way to partial mediation at three of the sites. This suggests that while the suppression effects originally observed in the U.S. *are* shared with some other societies, the effects of these five variables on the traditionalism-precautions relationship are parochial, and contingent on socio-political dynamics and perceptions that vary widely across societies.

**Figure 3:**
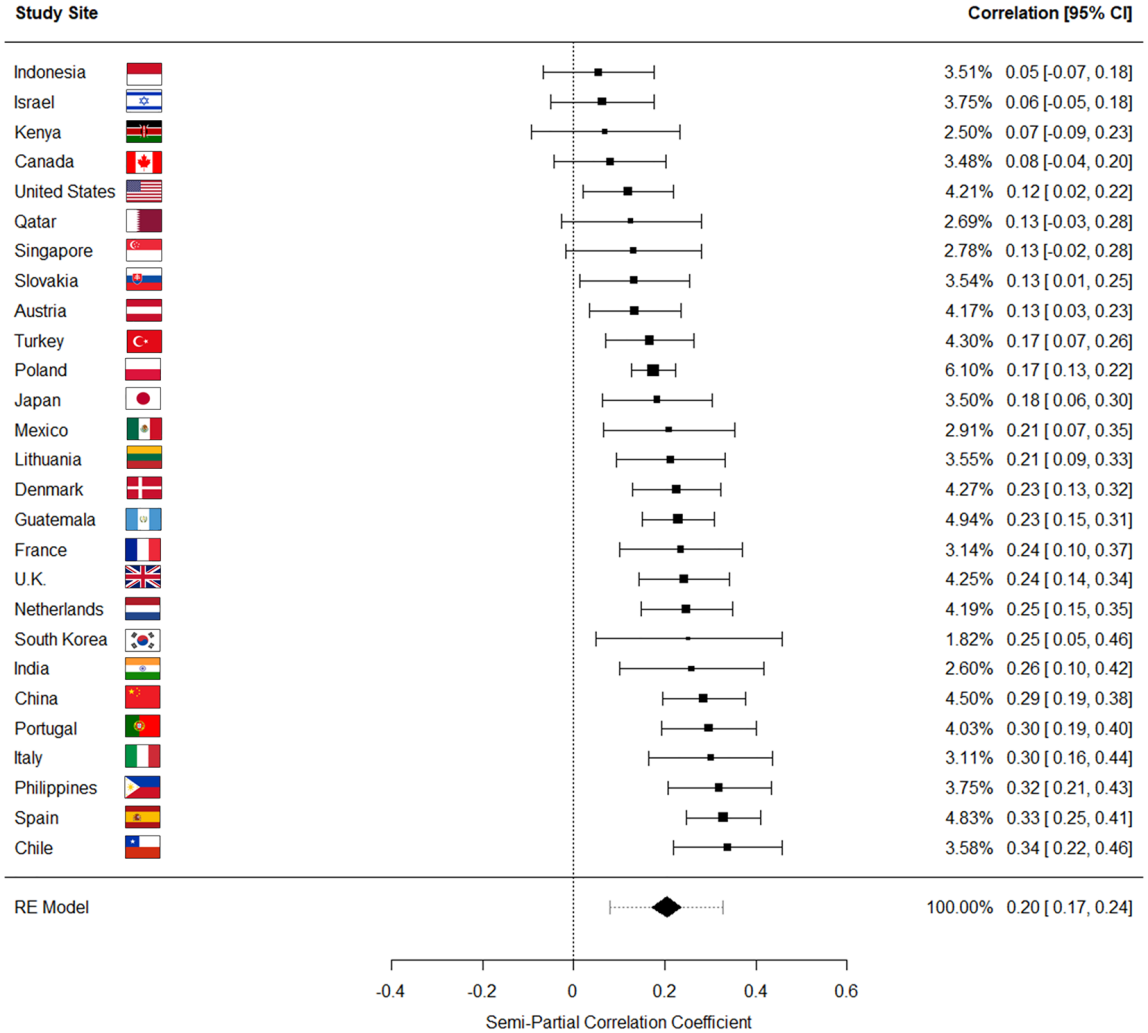
Results of a random effects, restricted maximum likelihood meta-analysis in which each study site was treated as a separate sample. The plot shows semi-partial correlations^54,55^ between traditionalism and COVID-19 health precautions at each study site, after adjusting for the effects of the five identified suppressor variables in multiple linear regressions where health precautions were regressed on traditionalism and each of those five variables. Covariates were identical across study sites. Note that the semi-partial correlations indicate the variance in health precautions uniquely explained by the aspects of traditionalism separate from the five suppressor variables, and the effect sizes can be interpreted using the same metrics applied to product-moment correlations. See Fig. 1 for a description of how to interpret the forest plot. For the overall meta-analyzed point estimate, neither the 95% confidence interval nor the 95% prediction interval overlap with zero.

We then ran a new set of random effect meta-analyses examining the relationship between traditionalism and overall COVID-19 health precautions, adjusting for the joint effects of the five aforementioned variables (see Fig. 3). While the overall meta-analyzed point estimate was conceptually indistinguishable from the effect size of the zero-order meta-analysis, accounting for the five variables resulted in the following observations: (a) the amount of heterogeneity in effect sizes across study sites was substantially reduced (I^2^ = 56.39%; 95% prediction interval [0.08, 0.33]); (b) the 95% prediction intervals suggest that if similar nations were randomly added to the sample, their true effect sizes would be positive and significant if adjusted for the five variables; and (c) the traditionalism-precautions relationship was now positive and significant in 21 out of 27 study sites. Taken together, these results suggest that the suppressive effects of these five variables emerge in a variety of socio-political contexts across the countries included in this study, and adjusting for their effects reveals a more consistent positive relationship in the direct pathway between pathogen avoidance and traditionalism across societies in our models. Note that these results remain robust after accounting for the same demographic and COVID-19-related covariates used previously (see Fig. S6), as well as when disattenuating for scale unreliability (see Fig. S8); when using factor scores in place of raw average composites (see Supplement page S62); and when conducting item-by-item analyses of the traditionalism composite items (see Supplement page S44).

### External-facing versus internal-facing precautions

As discussed in the “Methods” section, exploratory factor analysis revealed that the COVID-19 health precaution items can be decomposed into two factors, interpretable as distinguishing between actions in which other actors are salient, and which are often publicly visible (e.g., mask wearing and social distancing; hereafter *external-facing precautions*), versus actions in which other actors are not salient, and which often occur in private (e.g., hand washing and surface disinfection; hereafter *internal-facing precautions*). Because we did not predict this factor structure in advance, and therefore did not have a priori predictions about how it would affect the precautions-traditionalism relationship, the following analyses are exploratory.

**Figure 4:**
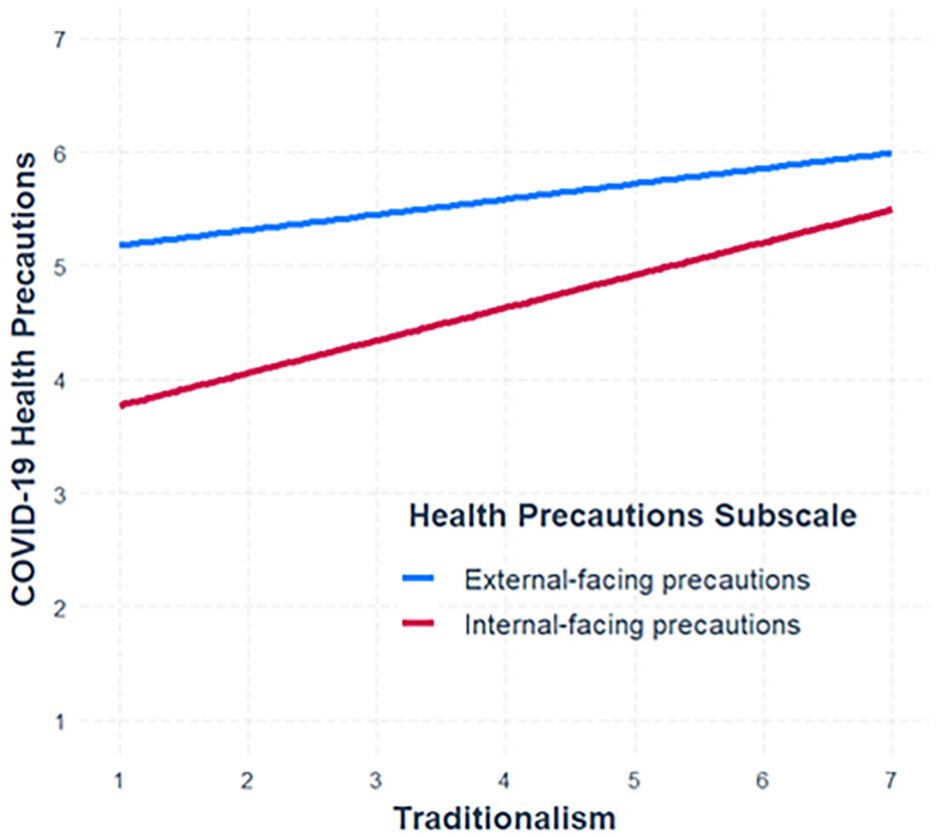
Results of a restricted maximum likelihood moderated mixed linear regression in which COVID-19 health precautions were regressed on traditionalism, a health precautions indicator variable (e.g., either internal-facing or external-facing), and the interaction between those two variables in the pooled sample. The model included participants nested within study sites as random effects. To test this interaction, there were two observations for each participant; the first observation contained each participants’ internal-facing precautions score, and the second their external-facing precautions score. We simultaneously created an indicator variable specifying which health precautions subscale corresponded with each observation. Simple slopes were then plotted in the figure. There was an interaction between health precautions subscale and traditionalism (*B* = 0.16, SE = 0.01, *t*(7,535) = 12.76, *p* < 0.001). A simple slopes analysis revealed that the correlation between traditionalism and internal-facing precautions (*B* = 0.29, SE = 0.01, *t*(7,535) = 23.17, *p* < 0.001) was about twice as strong as the correlation between traditionalism and external-facing precautions (*B* = 0.14, SE = 0.01, *t*(7,535) = 10.84, *p* < 0.001). Note that these results were robust to the inclusion of demographic and COVID-19-related covariates, and they were not conceptually affected when the five suppressor variables were included as covariates (see Supplement page S26). Further, results did not conceptually change when using factor scores instead of averaged composites (see Supplement page S63). Finally, we considered the possibility that the presence—or lack of presence—of planning precautions may be confounding our interpretation of the external- and internal-facing precautions subscales. Specifically, the internal-facing subscale has more items related to planning precautionary behaviors (such as the importance of obtaining prophylactic supplies), whereas the external-facing subscale has more items related to actual precautionary behavior (such as wearing a mask when outside the home). To address this possibility, we created a modified internal-facing precautions composite that excluded all planning-related precautions. Using the planning-less internal-precautions composite did not conceptually affect these results (see Supplement S26), suggesting that planning behaviors versus actual behaviors are not confounding our explanation for the moderating effect of external- versus internal-facing precautions.

To examine whether the relationship between traditionalism and COVID-19 precautionary behaviors varies as a function of whether precautions are external- or internal-facing, we assessed whether subscale moderated the traditionalism-precautions relationship in a mixed linear regression. We found that the strength of the traditionalism-precautions relationship was greater for internal-facing precautions relative to external-facing precautions (see Fig. 4).

## Discussion

Consistent with a postulated link between traditionalism and motivations to mitigate dangers, across 27 nations, we found evidence that at the individual level, traditionalism associates positively with health precautions aimed at a global pathogen threat. In addition, in some socio-political contexts, perceived tradeoffs between health precautions and priorities concerning the economy, personal liberties, and the ability to practice traditions statistically suppressed the zero-order relationship between traditionalism and COVID-19 precautions, as did low trust in scientists and high social dominance orientation. Importantly, accounting for the effects of the suppressor variables resulted in a more consistent positive correlation between traditionalism and COVID-19 precautions. This suggests that when individuals’ weightings of the costs, benefits, and tradeoffs of pathogen-threat mitigation and competing priorities—many of which are themselves tied to traditionalism—are taken into account, statistical associations between traditionalism and pathogen avoidance are more likely to be detected within any given cultural context.

These results both support the traditional norms account of the relationship between traditionalism and threat avoidance, and underscore the importance of parochial, countervailing preferences, many of which concern competing threat responses. Understanding the weights accorded to the costs and benefits of particular pathogen-avoidance behaviors in the real world is thus critical when assessing the extent to which traditionalism and pathogen avoidance covary among individuals. As expected, we found considerable heterogeneity in effect sizes across study sites, further highlighting the importance of parochial factors, and the contribution of cultural variation in shaping traditionalism-pathogen avoidance relationships. Indeed, given the nested relationship between cultural evolution and the production of traditional norms, any evolutionary explanation for relationships between pathogen avoidance and traditionalism must take into account the possibility of substantial variation within cultures across contexts, and across cultures. For example, the extent to which traditions protect against pathogen threats may depend in part upon the content of those traditions, either via traditions’ instrumental effects, or via the effects of adherence on ingroup cooperation and/or coordination.

Consistent with prior research on the tradeoffs attending COVID-19 prophylactic behaviors^29^, we found that the relationships between traditionalism and COVID-19 precautions were stronger for internal-facing precautions (e.g., hand washing) than for external-facing precautions (e.g., mask wearing). This may owe to differences in the extent to which these two types of precautions are constrained by factors outside of personal control. Because external-facing precautions are more likely to be regulated by government rules—such as mask mandates—individuals may have less leeway to align their behavior with their preferences. Alternately, because they are more likely to conflict with the pursuit of a wide variety of benefits obtained through sociality, external-facing precautions may reflect valuation of the latter to a greater extent than internal-facing precautions. Indeed, external-facing precautions are probably more likely to clash with traditions, as precautions such as social distancing will often interfere with activities such as traditional religious practices. There are thus multiple plausible potential reasons why traditionalism covaries with external-facing precautions to a lesser extent than with internal-facing ones.

This study has multiple limitations. First, samples were recruited on the basis of convenience, and were not representative of their countries more broadly. In particular, given that participants needed access to computing devices and internet connectivity, and because some samples were comprised of students, socio-economic status and levels of formal education are not representative. Of equal importance, in addition to a lack of representativeness within study sites, the countries included were not globally representative. Countries from the Global North were overrepresented, while countries from Africa and South America were especially underrepresented. In both cases, our sampling procedures limit the generalizability of our findings. In particular, the relatively high frequency at which suppression was observed using a limited variable set derived from prior work conducted in the U.S. may reflect the over-representation of countries having shared cultural and political histories.

The effect sizes that we observed, though analogous in magnitude to those obtained in similar previous research^7,10^, are relatively small. This likely owes in part to the fact that traditionalism is complex and multidetermined, and variation in it is not solely explained by pathogen-avoidance motivations. The same logic applies with regard to COVID-19 health precautions. Other sources of measurement error are also possible, such as the translatability and coherence of folk concepts and terminologies across societies and languages. In particular, our use of a broad but shallow assessment of traditionalism was likely one source of noise.

We measured the general proclivity to endorse one’s society’s traditions without examining the actual content of those traditions. This facilitated comparison across study sites irrespective of the particulars of any given society’s traditions; point estimates indicate the relationships between traditionalism and precautions as construed at each particular study site. Nevertheless, by leaving the content of those traditions unspecified, this approach is unable to explore the rich cultural particulars that may importantly drive variation across study sites. Such particulars likely vary markedly across social contexts and across cultures. Hence, we think it is inappropriate to closely compare the magnitudes of precise point estimates between the 27 study sites, or test causal explanations for heterogeneity in those estimates, especially given the issue of non-independence in country-level analyses^49^. Additionally, our samples were collected on a convenience basis, and none can be considered nationally representative. Although putative cultural dimensions such as tightness-looseness and collectivism-individualism might plausibly moderate the individual-level relationship between traditionalism and COVID-19 precautions^36,50^, for all of the aforementioned reasons, these data are not structured in such a way as to test nation-level hypotheses. Relatedly, it is beyond the purview of this project to unpack why effects may have obtained in specific study sites but not others, although we encourage future research that delves into particular social contexts more deeply, as well as possible culture-level moderators.

We examined only a relatively narrow set of possible suppressor variables, selected on the basis of their effects in previous research in the U.S. Our intention was to use these variables to probe whether, across diverse cultural contexts, cost–benefit tradeoffs and conflicting attitudes could influence traditionalism-pathogen avoidance relationships, rather than to exhaustively document all such possible tradeoffs. The latter would have been impractical in the present project given the large number of study sites and the diverse parochial factors germane to tradeoffs, and subjective weightings of those tradeoffs, entailed by COVID-19 precautions. Future studies, focused more narrowly on one or a small number of societies, should explore such tradeoffs in detail, including the extent to which politicization influences how individuals perceive cost–benefit structures.

Future work should elucidate the proximate mechanisms linking traditionalism and threat responsivity. Are traditionalists prone to perceive threats as relatively more attention-grabbing, and/or important, and/or susceptible to resolution through threat-mitigating action? Or, given the established links between traditionalism and respect for authority figures^1^, might traditionalists simply be more adherent to the directives of relevant leaders in times of crisis? Relatedly, traditionalism may be linked with a propensity for collective coalitional action which facilitates threat-responsive behaviors in concert with others. The extent to which any or all of these complementary potential pathways contribute to the link between traditionalism and pathogen-avoidance is currently unknown. More broadly, whereas we have focused here on a real-world pathogen threat, might comparable dynamics obtain with regard to traditionalism and the propensity to take action in response to threats in other domains, such as intergroup conflict or resource scarcity?

We have approached the construct of traditionalism in an underspecified manner loosely isomorphic with a folk concept of “tradition” that recurs reliably across societies. Having found a cross-culturally replicable association, we encourage investigators to explore the particular facets of traditionalism driving the relationship with COVID-19 precautions. Are there specific in-group practices and/or beliefs of perceived antiquity (i.e., traditions) more closely associated with threat responsivity? If so, are these contingent on the nature of distinct threat domains? (E.g., are the components of traditionalism driving associations with pathogen avoidance distinct from components associated with threat responses to intergroup conflict?) Future work should examine which aspects encompassed by the superordinate construct of tradition are most strongly linked with pathogen-threat responsivity, as well responsivity to contrastive threats. Such work may require focusing on fewer societies to allow more detailed consideration of the relative contributions of parochial beliefs and practices.

Ours is the first study to systematically investigate the relationship between traditionalism and avoidance of a specific infectious disease across a wide range of societies, attending to the kinds of costly, real-world behaviors that reflect the tradeoffs that shape actual decision making. Examining these phenomena at a global scale, we required methods that were coarse with regard to the particulars of the pandemic and its interactions with traditions in any one cultural setting. Despite this lack of granularity, consistent with the thesis that individual differences in the propensity to adhere to traditions are driven in part by differences in threat responsivity, we found evidence of a positive direct relationship between traditionalism and avoidance of a specific disease. When the individual and/or social contexts facilitated the alignment of traditionalism and health precautions, we observed that relationship at the zero order without needing to take other factors into account. When other preferences were perceived to clash with public health measures against COVID-19, stronger positive relationships between traditionalism and health precautions were detected in many cases after the effects of those clashing objectives were held constant.

Our findings have practical relevance for public health authorities and clinicians seeking to promulgate behavior changes that slow the spread of a disease that has claimed over six million victims worldwide. Whereas casual reflection might suggest that those who adhere to values and practices rooted in the past would be more hesitant to change behaviors or utilize new medical resources in the service of protecting themselves and others from a novel illness, in actuality, these may be the very people for whom, all else being equal, threats such as those posed by COVID-19 evoke mitigating action. The challenge may be that the same disposition to respond to this pathogen threat may also incline traditionalists to respond to other threats having conflicting mitigation requirements. It is thus crucial to recognize and address potential conflicts or tradeoffs that may inhibit tradition-minded individuals from adopting vital prophylactic and treatment practices beneficial to themselves, their societies, and the global community. More broadly, understanding the relationship between traditionalism and the extent to which danger prompts corrective action may prove vital as humanity confronts worldwide threats, from emerging pandemics to climate change, that can only be overcome through innovation and the adoption of new practices.

## Methods

### Project overview

This study was approved by the UCLA Office of the Human Research Protection Program, and all methods were performed in accordance with relevant guidelines and regulations. Informed consent was obtained before participation. Complete questionnaire in English, translations, datasets, analysis code, and preregistrations of predictions and methods are available at https://osf.io/6vu5b/?view_only=873259d429c346d2912303fc44df5079. See Supplement page S1 for a list of questionnaire items and composite scales.

Adult participants were recruited online for an observational, cross-sectional survey-based study between October 2020 and July 2021 in 27 countries, with a final N of 7844. Countries were selected on a convenience basis, and both the range of possible study sites and the representativeness of samples recruited at each were constrained by our use of remote internet-mediated interactions for recruitment and participation. Nevertheless, we endeavored to collect data in a wide range of societies, selected from diverse major culture areas; see Fig. S1. Where appropriate, survey materials were translated from English by fluent bilingual speakers. While most participants were unpaid volunteers, recruitment and compensation schemes varied across study sites. A mix of non-student and student populations were used, depending on the study site. See Table S3 in the supplementary materials for a summary of study sites, study site-specific Ns, exclusions, as well as full information on survey languages, recruitment procedures, and participant demographics for each study site. Data were prescreened for minimum completeness and correct answers to attention checks.

### Measures

Measures were consistent across study sites, with some small deviations where necessary (e.g., items addressing education levels differed across study sites according to the local education structure). A full list of these differences can be found on the OSF repository (see link above).

#### COVID-19 health precautions

COVID-19 health precautions were measured with a 13-item scale examining participants’ self-reported real-world behaviors. Questions addressed behaviors which, at the time, were widely thought by public health authorities to have significant protective value against COVID-19 (e.g., the frequency of mask wearing, hand washing, and social distancing, as well as the importance to the participant of stocking up on supplies such as hand sanitizer). Items were rated on 7-point scales, either from “never” to “as often as possible”, or from “not important at all”, to “extremely important”. Based on the results of an exploratory factor analysis (see Table S4), a composite COVID-19 health precautions variable was created for the purposes of analysis by averaging across the thirteen items. The factor analysis also revealed that this scale can be subdivided into two subscales: *external-facing health precautions* (e.g., observing mask wearing and social distancing), and *internal-facing health precautions* (e.g., washing hands). These factors are consistent with results from prior research on COVID-19 precautions^29^. Main text analyses report results using the combined composite, unless otherwise noted. See Supplement page S31 for details on scale development and factor analysis.

#### Traditionalism

Because we were unable to identify a culturally neutral traditionalism scale in the prior literature, we drew upon two instruments that had previously been deployed in large-scale cross-cultural research. These scales jointly assessed the concept of traditionalism, or the tendency to endorse and place importance on the practice of traditional norms. To increase comparability across study sites, questions were designed to measure participants’ general tendency to endorse or reject their own society’s traditional social norms and values. The two scales were the conventionalism subscale of the Aggression-Submission-Conventionalism scale^51^, which measures the general tendency to endorse one’s society’s traditional social norms without specifying the content of those traditions (e.g., “Traditions are the foundation of a healthy society and should be respected”), as well as items from the authority subscale from the Moral Foundations Questionnaire Short Version^52,53^, which similarly assesses whether individuals respect traditions and authorities, both generally (e.g., “To what extent are the following considerations relevant to your thinking… Whether or not someone conformed to the traditions of society”), and in relation to specific values regarding gender and age roles (e.g., “Respect for authority is something all children need to learn”). Items were rated on 7-point scales, either from “Not at all relevant” to “Extremely relevant”, or from “Strongly Disagree” to “Strongly Agree”. After conducting an exploratory factor analysis on items from both scales jointly (see Table S7), a six-item averaged composite traditionalism variable was computed for analyses involving traditionalism. See Supplement page S39 for details on scale development and factor analysis.

#### Potential suppressor variables

We included seven variables related to potential perceived conflicts between COVID-19 health precautions and other priorities: distrust in science regarding the COVID-19 pandemic; SDO (measured using the 4-item short form scale^30^); concern about the effects of the COVID-19 pandemic on the economy and personal liberties; and perceptions that COVID-19 health precautions were clashing with personal liberties, one’s own traditions, and the health of the economy, respectively. Unless otherwise noted, these variables were measured using single items.

#### Demographics, COVID-19-related covariates, and attention checks

Participants indicated their gender identity and age, and their income relative to others in their country. Education was also measured, but because different countries in the study have different educational systems, levels of education examined varied across study sites. For the purposes of analysis, education was therefore coded into a universal four-level structure: primary school, secondary school, undergraduate-level, and postgraduate-level. We also measured a number of covariates relevant to the pandemic itself, including perceived COVID-19 prevalence in participants’ local communities; the population density of those communities; whether participants’ jobs required that they leave the home; and whether participants had certain pre-existing medical conditions that may put them at higher risk for severe disease. Finally, we included several attention checks.

## Supplementary Information

Supplementary Information.

## Data Availability

All relevant data are openly available via the Open Science Framework at the following link: https://osf.io/6vu5b/?view_only=873259d429c346d2912303fc44df5079.

## References

[1] Duckitt J, Bizumic B, Krauss SW, Heled E (2010). A tripartite approach to right-wing authoritarianism: The authoritarianism-conservatism-traditionalism model. Polit. Psychol..

[2] Giuliano, P. & Nunn, N. *Understanding Cultural Persistence and Change*. w23617 http://www.nber.org/papers/w23617.pdf (2017) 10.3386/w23617.

[3] Claessens S, Fischer K, Chaudhuri A, Sibley C, Atkinson Q (2020). The dual evolutionary foundations of political ideology. Nat. Hum. Behav..

[4] Jost JT, Federico CM, Napier JL (2009). Political ideology: Its structure, functions, and elective affinities. Annu. Rev. Psychol..

[5] Hibbing JR, Smith KB, Alford JR (2014). Differences in negativity bias underlie variations in political ideology. Behav. Brain Sci..

[6] Murray DR, Schaller M (2012). Threat(s) and conformity deconstructed: Perceived threat of infectious disease and its implications for conformist attitudes and behavior. Eur. J. Soc. Psychol..

[7] Tybur JM (2016). Parasite stress and pathogen avoidance relate to distinct dimensions of political ideology across 30 nations. Proc. Natl. Acad. Sci..

[8] Fischer, K., Chaudhuri, A. & Atkinson, Q. *Responses to the COVID-19 Pandemic Reflect the Dual Evolutionary Foundations of Political Ideology*. https://psyarxiv.com/qeap8/ (2020) doi:10.31234/osf.io/qeap8. 10.1038/s41562-020-0850-9 32231279

[9] Makhanova A, Plant EA, Monroe AE, Maner JK (2019). Binding together to avoid illness: Pathogen avoidance and moral worldviews. Evol. Behav. Sci..

[10] Samore T, Fessler DMT, Sparks AM, Holbrook C (2021). Of pathogens and party lines: Social conservatism positively associates with COVID-19 precautions among U.S. Democrats but not Republicans. PLoS ONE.

[11] Henrich J, McElreath R (2003). The evolution of cultural evolution. Evol. Anthropol. Issues News Rev..

[12] Murray DR, Fessler DMT, Kerry N, White C, Marin M (2017). The kiss of death: Three tests of the relationship between disease threat and ritualized physical contact within traditional cultures. Evol. Hum. Behav..

[13] Henrich, J. *A Cultural Species: How Culture Drove Human Evolution*. 10.1037/e519392012-002 (2011).

[14] Zwirner E, Thornton A (2015). Cognitive requirements of cumulative culture: Teaching is useful but not essential. Sci. Rep..

[15] Navarrete CD, Fessler DMT (2005). Normative bias and adaptive challenges: A relational approach to coalitional psychology and a critique of terror management theory. Evol. Psychol..

[16] Fessler DMT, Carruthers P, Laurence S, Stich S (2006). Steps toward an evolutionary psychology of a culture-dependent species. The Innate Mind: Volume 2: Culture and Cognition.

[17] Mcelreath R, Boyd R, Richerson PJ (2003). Shared norms and the evolution of ethnic markers. Curr. Anthropol..

[18] Sugiyama LS (2004). Illness, injury, and disability among Shiwiar forager-horticulturalists: implications of health-risk buffering for the evolution of human life history. Am. J. Phys. Anthropol..

[19] Jost JT, Glaser J, Kruglanski AW, Sulloway FJ (2003). Political conservatism as motivated social cognition. Psychol. Bull..

[20] Conover PJ, Feldman S (1981). The Origins and Meaning of Liberal/Conservative Self-Identifications. Am. J. Polit. Sci..

[21] Wilson GD (1973). The Psychology of Conservatism.

[22] Terrizzi JA, Shook NJ, McDaniel MA (2013). The behavioral immune system and social conservatism: A meta-analysis. Evol. Hum. Behav..

[23] Jost JT, Stern C, Rule NO, Sterling J (2017). The politics of fear: Is there an ideological asymmetry in existential motivation?. Soc. Cogn..

[24] Jost JT (2017). Ideological asymmetries and the essence of political psychology. Polit. Psychol..

[25] Duncan LA, Schaller M, Park JH (2009). Perceived vulnerability to disease: Development and validation of a 15-item self-report instrument. Personal. Individ. Differ..

[26] Olatunji BO (2009). Confirming the three-factor structure of the disgust scale—Revised in eight countries. J. Cross-Cult. Psychol..

[27] Karinen, A., Tybur, J. M. & de Vries, R. E. The disgust traits: Self-other agreement in pathogen, sexual, and moral disgust sensitivity and their independence from HEXACO personality. 10.1037/emo0000795 (2019). 10.1037/emo0000795 34043408

[28] Tybur JM, Lieberman D, Fan L, Kupfer TR, de Vries RE (2020). Behavioral immune trade-offs: Interpersonal value relaxes social pathogen avoidance. Psychol. Sci..

[29] Gul P (2021). Disease avoidance motives trade-off against social motives, especially mate-seeking, to predict social distancing: Evidence from the COVID-19 pandemic. Soc. Psychol. Personal. Sci..

[30] Ritchie, H. *et al.* Coronavirus pandemic (COVID-19). *Our World Data* (2020).

[31] Hensel L (2022). Global behaviors, perceptions, and the emergence of social norms at the onset of the COVID-19 pandemic. J. Econ. Behav. Organ..

[32] Czeisler MÉ (2020). Mental health, substance use, and suicidal ideation during the COVID-19 pandemic—United States, June 24–30, 2020. MMWR Morb. Mortal. Wkly. Rep..

[33] Haselton MG, Buss DM (2000). Error management theory: A new perspective on biases in cross-sex mind reading. J. Pers. Soc. Psychol..

[34] Galperin A, Haselton MG (2013). Error management and the evolution of cognitive bias. Social Thinking and Interpersonal Behavior.

[35] Neuberg SL, Kenrick DT, Schaller M (2011). Human threat management systems: Self-protection and disease avoidance. Neurosci. Biobehav. Rev..

[36] Gelfand MJ (2021). The relationship between cultural tightness–looseness and COVID-19 cases and deaths: A global analysis. Lancet Planet. Health.

[37] Karwowski M (2020). When in danger, turn right: Does COVID-19 threat promote social conservatism and right-wing presidential candidates?. Hum. Ethol..

[38] Rosenfeld DL, Tomiyama AJ (2021). Can a pandemic make people more socially conservative? Political ideology, gender roles, and the case of covid-19. J. Appl. Soc. Psychol..

[39] Leeuwen, F. van, Jaeger, B., Sleegers, W. & Petersen, M. B. Do experimental manipulations of pathogen avoidance motivations influence conformity? 10.31234/osf.io/t3bcw (2021). 10.1177/01461672231160655 PMC11143762 36945750

[40] Pennycook G, McPhetres J, Bago B, Rand DG (2021). Beliefs about COVID-19 in Canada, the United Kingdom, and the United States: A novel test of political polarization and motivated reasoning. Pers. Soc. Psychol. Bull..

[41] Carothers, T. & O’Donohue, A. Polarization and the Pandemic. *Carnegie Endowment for International Peace* https://carnegieendowment.org/2020/04/28/polarization-and-pandemic-pub-81638 (2020).

[42] Kerr JR, Wilson MS (2021). Right-wing authoritarianism and social dominance orientation predict rejection of science and scientists. Group Process. Intergroup Relat..

[43] Pratto F (2013). Social dominance in context and in individuals: Contextual moderation of robust effects of social dominance orientation in 15 languages and 20 countries. Soc. Psychol. Personal. Sci..

[44] Roccato M, Ricolfi L (2005). On the correlation between right-wing authoritarianism and social dominance orientation. Basic Appl. Soc. Psychol..

[45] Wilson MS, Sibley CG (2013). Social dominance orientation and right-wing authoritarianism: Additive and interactive effects on political conservatism: SDO, RWA, and conservatism. Polit. Psychol..

[46] Spineli LM, Pandis N (2020). Prediction interval in random-effects meta-analysis. Am. J. Orthod. Dentofacial Orthop..

[47] Hunter, J. & Schmidt, F. *Methods of Meta-analysis Corrected Error and Bias in Research Findings*. *Educational Researcher* vol. 20 (2004).

[48] MacKinnon DP, Krull JL, Lockwood CM (2000). Equivalence of the mediation, confounding and suppression effect. Prev. Sci. Off. J. Soc. Prev. Res..

[49] Claessens, S. & Atkinson, Q. The non-independence of nations and why it matters. 10.31234/osf.io/m6bsn (2022).

[50] Maaravi Y, Levy A, Gur T, Confino D, Segal S (2021). “The tragedy of the commons”: How individualism and collectivism affected the spread of the COVID-19 pandemic. Front. Public Health.

[51] Dunwoody P, Funke F (2016). The aggression-submission-conventionalism scale: Testing a new three factor measure of authoritarianism. J. Soc. Polit. Psychol..

[52] Graham J (2011). Mapping the moral domain. J. Pers. Soc. Psychol..

[53] Graham, J., Haidt, J. & Nosek, B. A. Questionnaires | Moral Foundations Theory. https://moralfoundations.org/questionnaires/ (2008).

[54] Aloe AM, Thompson CG (2013). The synthesis of partial effect sizes. J. Soc. Soc. Work Res..

[55] Pituch KA, Stevens JP (2016). Applied Multivariate Statistics for the Social Sciences.

