# Supplementary material for: Greater traditionalism predicts COVID-19 precautionary behaviors across 27 societies
Source: Sci Rep. 2023 Apr 11;13:4969. doi: 10.1038/s41598-023-29655-0 (PMC10090070; doi:10.1038/s41598-023-29655-0)
Supplement: Supplementary file 1 — Supplementary Information. [file 41598_2023_29655_MOESM1_ESM.docx]

**Supplementary Information for**

Greater Traditionalism Predicts COVID-19 Precautionary Behaviors Across 27 Societies.

Theodore Samore*, Daniel M.T. Fessler, Adam Maxwell Sparks, Colin Holbrook, Lene Aarøe, Carmen Gloria Baeza, María Teresa Barbato, Pat Barclay, Renatas Berniūnas**,** Jorge Contreras-Garduño, Bernardo Costa-Neves, Maria del Pilar Grazioso, Pınar Elmas, Peter Fedor, Ana Maria Fernandez, Regina Fernández-Morales, Leonel Garcia-Marques, Paulina Giraldo-Perez, Pelin Gul, Fanny Habacht, Youssef Hasan, Earl John Hernandez, Tomasz Jarmakowski, Shanmukh Kamble, Tatsuya Kameda, Bia Kim, Tom R. Kupfer, Maho Kurita, Norman P. Li, Junsong Lu, Francesca R. Luberti, María Andrée Maegli, Marinés Mejia, Coby Morvinski, Aoi Naito, Alice Ng’ang’a, Angélica Nascimento de Oliveira, Daniel N. Posner, Pavol Prokop, Yaniv Shani, Walter Omar Paniagua Solorzano, Stefan Stieger, Angela Oktavia Suryani, Lynn K.L. Tan, Joshua M. Tybur, Hugo Viciana, Amandine Visine, Jin Wang, Xiao-Tian Wang

***Corresponding author:** theo.samore@gmail.com

Contents

[Supplementary Procedure 1](#_Toc122089797)

[1. Composite scales and other variables 1](#_Toc122089798)

[2. Differences between pre-registration and final manuscript 6](#_Toc122089799)

[3. Analysis software 7](#_Toc122089800)

[4. Software version and source information 7](#_Toc122089801)

[Map of Study Sites 14](#_Toc122089802)

[Analyses Supporting Main Text 14](#_Toc122089803)

[1. Traditionalism-precautions correlations and density distributions by study site 14](#_Toc122089804)

[2. Traditionalism-precautions relationship adjusting for covariates 17](#_Toc122089805)

[3. Precautions-traditionalism relationship disattenuated for unreliability 21](#_Toc122089806)

[4. Effects of COVID-19 prevalence on study estimates of traditionalism-precautions relationship 23](#_Toc122089807)

[5. Identifying suppressor variables 23](#_Toc122089808)

[6. Precautions subscale interaction adjusting for covariates, suppressor variables, and planning items 26](#_Toc122089809)

[7. Summary statistics and other information by study site 27](#_Toc122089810)

[8. COVID-19 health precautions scale development 31](#_Toc122089811)

[9. Traditionalism scale development 39](#_Toc122089812)

[10. Traditionalism-precautions relationship using factor scores 63](#_Toc122089813)

[11. Suppressor variable descriptives by country 66](#_Toc122089814)

[Additional Analyses 72](#_Toc122089815)

[1. COVID-19 precautions and gender 72](#_Toc122089816)

[References 76](#_Toc122089817)

## Supplementary Procedure

### Composite scales and other variables

Full survey items in English can be found in the open archives, as well as all translations.

*COVID-19 Public Health Precautions Composite:* Composite of both external- and internal-facing precautions.

Note that although the following item—“When you leave your home and may be near other people, how often do you… wear gloves”—was included as a precaution item in the survey, it did not load onto either factor, and therefore was not included in any composite.

Internal-facing precautions:

Compared to before the pandemic, how important has it been for you to have adequate supplies of... [1 – not at all important … 7 – extremely important]

1. Cleaning supplies (such as bleach, disinfectant spray, disinfectant wipes, etc.)
2. Hand sanitizer/hand soap
3. Masks and gloves

Compared to before the pandemic, how important has been for you to… [1 – not at all important … 7 – extremely important]

1. Clean your hands with soap or sanitizer
2. Disinfect surfaces in your house, like doorknobs or counters
3. Eat or drink things to boost your immune system

When you leave your home and may be near other people, how often do you each of the following? [1 – never … 7 – as often as possible]

1. Disinfect surfaces upon returning home

External-facing precautions:

When you leave your home and may be near other people, how often do you each of the following? [1 – never … 7 – as often as possible]

1. Wear a mask and/or face shield/visor
2. Stay farther than 2 meters/6 feet away from people [*note:* *unit of distance varied according to local norms]*

To what degree were you careful in the last week to avoid interaction with people outside your household? [1 – not careful at all … 7 – as careful as possible]

In your daily life, how important is it that you take actions that protect yourself and others from COVID-19? [1 – not at all important … 7 – extremely important]

Compared to before the pandemic, I have changed many aspects of my everyday behavior to protect myself and others from COVID-19 [1 – strongly disagree … 7 – strongly agree]

*Religious precautions:*

How often do you engage in… [1 – never … 7 – very frequently]

1. Individual religious behavior such as prayer (for example praying alone) to protect yourself and others from COVID-19
2. Collective religious behavior such as attending a church/synagogue/mosque/temple/shrine to protect yourself and others from COVID-19

*Conventionalism:* From the Aggression-Submission-Conventionalism scale^1^, a measure of right-wing authoritarianism.

The following questions concern values that people may or may not hold. Please select a number to indicate the degree to which you agree or disagree with each statement. [1 – strongly disagree … 7 – strongly agree]

1. People emphasize tradition too much. (r)
2. It would be better for society if more people followed social norms.
3. People should respect social norms.
4. Traditions are the foundation of a healthy society and should be respected.
5. Traditions interfere with progress. (r)
6. People should challenge social traditions in order to advance society. (r)

*Moral Foundations authority subscale:* Short-form measure^2^.

When you decide whether something is right or wrong, to what extent are the following considerations relevant to your thinking? Please rate each statement using this scale: [1 – not at all relevant … 7 – extremely relevant]

1. Whether or not someone showed a lack of respect for authority
2. Whether or not someone conformed to the traditions of society

Please read the following sentences and indicate your agreement or disagreement [1 – strongly disagree … 7 – strongly agree]

1. Men and women each have different roles to play in society.
2. Respect for authority is something all children need to learn.

*Traditionalism Factor:* Items derived from Conventionalism and Moral Foundations authority subscales.

- - - 1. It would be better for society if more people followed social norms (see anchors above).

1. People should respect social norms (see anchors above).
2. Traditions are the foundation of a healthy society and should be respected. (see anchors above).
3. Whether or not someone showed a lack of respect for authority (see anchors above).
4. Whether or not someone conformed to the traditions of society (see anchors above).
5. Respect for authority is something all children need to learn (see anchors above).

*Social dominance orientation composite:* Four-item Short Social Dominance Orientation Scale^3^

There are many types of groups in the world: men and women, ethnic and religious groups, nationalities, political factions, etc. Please select a number to rate the degree to which you oppose or favor each statement about groups, where higher numbers mean you favor the statement more, and lower numbers mean you oppose the statement more. [1 – extremely oppose … 7 – extremely favor]

- - - 1. In setting priorities, we must consider all groups. (r)
      2. We should not push for group equality.

1. Group equality should be our ideal. (r)
2. Superior groups should dominate inferior groups.

*Single item suppressor variables:*

1. Distrust in scientists:

How much do you think scientists provide advice based on accurate information about what to do during the COVID-19 outbreak? [1 – not at all accurate … 7 – extremely accurate]

1. Concern over the effects of COVID-19 on the economy:

How concerned are you about the economic effects of the COVID-19 pandemic? [1 – not at all concerned … 7 – extremely concerned]

1. Concern over the effects of COVID-19 on personal liberties:

How concerned are you about losing personal rights because of the COVID-19 pandemic? [1 – not at all concerned … 7 – extremely concerned]

1. Perceived tradeoffs between the COVID-19 pandemic and personal rights

The public health benefits of policies addressing the COVID-19 pandemic are not worth the potential costs to personal rights [1 – strongly disagree … 7 – strongly agree]

1. Perceived tradeoffs between the COVID-19 pandemic and the economy

The public health benefits of policies addressing the COVID-19 pandemic are not worth the potential costs to the economy [1 – strongly disagree … 7 – strongly agree]

1. Perceived tradeoffs between the COVID-19 pandemic and practicing traditions

Following my traditional cultural practices is more important than following public health recommendations about COVID-19 when those guidelines interfere with my traditional cultural practices [1 – strongly disagree … 7 – strongly agree]

*COVID-19-relevant covariates:*

1. Perceived COVID-19 prevalence:

In your opinion, how prevalent is COVID-19 in your local community? [1 – not at all prevalent … 7 – extremely prevalent]

1. Population density:

How would you best describe the area where you live?

- Large city
- Small city
- Town or suburb
- Village or countryside

1. Job requirements:

If applicable, does your job currently require that you leave the home?

- Always required to leave the home
- Sometimes required to leave the home
- Rarely required to leave the home
- Never required to leave the home
- I don’t have a job

1. Health conditions:

Has a doctor or other health professional ever diagnosed you with any of the following health conditions?

- Autoimmune disease
- Weak immune system
- Diabetes
- High blood pressure
- Heart disease
- Asthma
- Kidney disease

*Demographic variables and attention checks:*

1. Gender (some response options differed across study sites, see OSF repository for details):

What is your gender identity?

- Woman
- Man
- Other

1. Education (Response options differed across study sites based on local education systems. For the purposes of analysis, those response options were binned into the following four categories. see OSF repository for details):

Your highest level of education completed?

- Primary school
- Secondary school
- Undergraduate level
- Advanced/post-graduate level

1. Age:

What is your age in years?

1. Relative wealth:

Compared to other people in your country, how would you describe your wealth? [1 – much less wealthy than most other people in my country … 7 – much wealthier than most other people in my country]

1. Attention check 1:

When you look up on a clear day, what color is the sky?

- Train station
- Laptop
- Blue
- Cardboard box
- Chicken
- Green
- Book
- Lamp

1. Attention check 2:

Did you carefully consider your responses to this survey (please be honest)?

- Yes
- No

### Differences between pre-registration and final manuscript

There are several differences between the pre-registered measures and those reported in the main text and supplement. Here, we explain those differences.

- *Survey items reserved for separate projects:* We included a number of measures in the surveys that are not reported in the main text because they are being reserved for separate projects. In addition to listing these reserved variables below, they can also be found in the full surveys in the open archive.

Reserved measures:

1. COVID-19 religious precautions subscale (see items above)
2. Pathogen disgust sensitivity scale^4^
3. Belief in a dangerous world scale^5^
4. Generalized social trust item (not included at every study site)
5. Social conservatism item (not included at every study site)
6. Economic conservatism item (not included at every study site)
7. Belief in a deity/deities (not included at every study site)
8. Various measures that were included at individual study sites only (see study-site specific full surveys in open archive for details).
9. Parental status

- *Unincluded study sites*: In addition to the 27 countries included in the manuscript, we pre-registered that we would collect data in the following additional countries: Russia, Brazil, Colombia, Egypt, and Armenia. However, these countries were not included in the final sample for a variety of unanticipated circumstances. In Armenia, Brazil, and Russia, data collection never began due to extenuating circumstances. In Egypt and Colombia, data collection began, but we were unable to recruit more than 60 participants in either country after exclusion criteria were applied. Therefore, they were excluded from the study, and the existing underpowered data was never analyzed in any way. We specified in the pre-registration that study sites may be excluded on the basis of insufficient participant recruitment.
- *COVID-19 infection status:* Participants were asked whether they were currently known to be infected with COVID-19. We intended to use this as a covariate with the other COVID-19-related covariates in relevant meta-analyses. However, at some study sites, no participants reported being infected with COVID-19. Therefore, it was dropped from analysis.

### Analysis software

We used R^6^, RStudio^7^, and the R-packages devtools^8^, ggplot2^9^, GPArotation , gridExtra^10^, interactions^11^, kableExtra^12^, lavaan^13^, lme4^14^, lmerTest^15^, MASS^16^, Matrix^17^, mediation^18^, metafor^19^, mvtnorm^20,21^, parameters^22^, psych^23^, report^24^, sandwich^25,26^, scales^27^, sjPlot^28^, and tidyverse^29^ for our analyses. The code that produced all analyses in the main text and supplement is openly available at: https://osf.io/6vu5b/?view_only=873259d429c346d2912303fc44df5079.

### Software version and source information

- Session info ---------------------------------------------------------------------------------------------------

version R version 4.1.1 (2021-08-10)

os Windows 10 x64 (build 19043)

system x86_64, mingw32

ui RStudio

language (EN)

collate English_United States.1252

ctype English_United States.1252

date 2022-06-18

rstudio 2021.09.0+351 Ghost Orchid (desktop)

pandoc 2.14.0.3 @ C:/Program Files/RStudio/bin/pandoc/ (via rmarkdown)

- Packages (attached & loaded via a namespace) ----------------------------------------------------------

package * version date (UTC) lib source

assertthat 0.2.1 2019-03-21 [1] CRAN (R 4.1.1)

backports 1.3.0 2021-10-27 [1] CRAN (R 4.1.1)

base * 4.1.1 2021-08-10 [?] local

base64enc 0.1-3 2015-07-28 [1] CRAN (R 4.1.1)

bayestestR 0.11.5 2021-10-30 [1] CRAN (R 4.1.2)

boot 1.3-28 2021-05-03 [2] CRAN (R 4.1.1)

brio 1.1.3 2021-11-30 [1] CRAN (R 4.1.3)

broom 0.7.9 2021-07-27 [1] CRAN (R 4.1.1)

bslib 0.3.1 2021-10-06 [1] CRAN (R 4.1.2)

cachem 1.0.6 2021-08-19 [1] CRAN (R 4.1.2)

callr 3.7.0 2021-04-20 [1] CRAN (R 4.1.1)

cellranger 1.1.0 2016-07-27 [1] CRAN (R 4.1.1)

checkmate 2.0.0 2020-02-06 [1] CRAN (R 4.1.1)

cli 3.3.0 2022-04-25 [1] CRAN (R 4.1.3)

cluster 2.1.2 2021-04-17 [2] CRAN (R 4.1.1)

coda 0.19-4 2020-09-30 [1] CRAN (R 4.1.2)

colorspace 2.0-2 2021-06-24 [1] CRAN (R 4.1.1)

compiler 4.1.1 2021-08-10 [2] local

crayon 1.4.1 2021-02-08 [1] CRAN (R 4.1.1)

data.table 1.14.2 2021-09-27 [1] CRAN (R 4.1.1)

datasets * 4.1.1 2021-08-10 [2] local

datawizard 0.2.3 2022-01-26 [1] CRAN (R 4.1.1)

DBI 1.1.1 2021-01-15 [1] CRAN (R 4.1.1)

dbplyr 2.1.1 2021-04-06 [1] CRAN (R 4.1.1)

desc 1.4.0 2021-09-28 [1] CRAN (R 4.1.2)

devtools * 2.4.3 2021-11-30 [1] CRAN (R 4.1.3)

digest 0.6.28 2021-09-23 [1] CRAN (R 4.1.1)

dplyr * 1.0.7 2021-06-18 [1] CRAN (R 4.1.1)

effectsize 0.6.0.1 2022-01-26 [1] CRAN (R 4.1.1)

ellipsis 0.3.2 2021-04-29 [1] CRAN (R 4.1.1)

emmeans 1.7.2 2022-01-04 [1] CRAN (R 4.1.2)

estimability 1.3 2018-02-11 [1] CRAN (R 4.1.1)

evaluate 0.14 2019-05-28 [1] CRAN (R 4.1.1)

fansi 0.5.0 2021-05-25 [1] CRAN (R 4.1.1)

fastmap 1.1.0 2021-01-25 [1] CRAN (R 4.1.1)

forcats * 0.5.1 2021-01-27 [1] CRAN (R 4.1.1)

foreign 0.8-81 2020-12-22 [2] CRAN (R 4.1.1)

Formula 1.2-4 2020-10-16 [1] CRAN (R 4.1.1)

fs 1.5.0 2020-07-31 [1] CRAN (R 4.1.1)

generics 0.1.1 2021-10-25 [1] CRAN (R 4.1.1)

ggeffects 1.1.1 2021-07-29 [1] CRAN (R 4.1.2)

ggplot2 * 3.3.5 2021-06-25 [1] CRAN (R 4.1.1)

glue 1.6.2 2022-02-24 [1] CRAN (R 4.1.3)

graphics * 4.1.1 2021-08-10 [2] local

grDevices * 4.1.1 2021-08-10 [2] local

grid 4.1.1 2021-08-10 [2] local

gridExtra * 2.3 2017-09-09 [1] CRAN (R 4.1.1)

gtable 0.3.0 2019-03-25 [1] CRAN (R 4.1.1)

haven 2.4.3 2021-08-04 [1] CRAN (R 4.1.1)

highr 0.9 2021-04-16 [1] CRAN (R 4.1.1)

Hmisc 4.6-0 2021-10-07 [1] CRAN (R 4.1.1)

hms 1.1.1 2021-09-26 [1] CRAN (R 4.1.1)

htmlTable 2.3.0 2021-10-12 [1] CRAN (R 4.1.1)

htmltools 0.5.2 2021-08-25 [1] CRAN (R 4.1.1)

htmlwidgets 1.5.4 2021-09-08 [1] CRAN (R 4.1.1)

httr 1.4.2 2020-07-20 [1] CRAN (R 4.1.1)

insight 0.15.0 2022-01-07 [1] CRAN (R 4.1.2)

interactions * 1.1.5 2021-07-02 [1] CRAN (R 4.1.2)

jpeg 0.1-9 2021-07-24 [1] CRAN (R 4.1.1)

jquerylib 0.1.4 2021-04-26 [1] CRAN (R 4.1.1)

jsonlite 1.7.2 2020-12-09 [1] CRAN (R 4.1.1)

jtools 2.1.4 2021-09-03 [1] CRAN (R 4.1.2)

kableExtra * 1.3.4 2021-02-20 [1] CRAN (R 4.1.1)

knitr 1.36 2021-09-29 [1] CRAN (R 4.1.1)

lattice 0.20-44 2021-05-02 [2] CRAN (R 4.1.1)

latticeExtra 0.6-29 2019-12-19 [1] CRAN (R 4.1.1)

lavaan * 0.6-9 2021-06-27 [1] CRAN (R 4.1.1)

lifecycle 1.0.1 2021-09-24 [1] CRAN (R 4.1.1)

lme4 * 1.1-27.1 2021-06-22 [1] CRAN (R 4.1.1)

lmerTest * 3.1-3 2020-10-23 [1] CRAN (R 4.1.2)

lpSolve 5.6.15 2020-01-24 [1] CRAN (R 4.1.1)

lubridate 1.8.0 2021-10-07 [1] CRAN (R 4.1.1)

magrittr 2.0.1 2020-11-17 [1] CRAN (R 4.1.1)

MASS * 7.3-54 2021-05-03 [2] CRAN (R 4.1.1)

mathjaxr 1.4-0 2021-03-01 [1] CRAN (R 4.1.2)

Matrix * 1.3-4 2021-06-01 [2] CRAN (R 4.1.1)

mediation * 4.5.0 2019-10-08 [1] CRAN (R 4.1.1)

memoise 2.0.1 2021-11-26 [1] CRAN (R 4.1.3)

metafor * 3.0-2 2021-06-09 [1] CRAN (R 4.1.2)

methods * 4.1.1 2021-08-10 [2] local

minqa 1.2.4 2014-10-09 [1] CRAN (R 4.1.1)

mnormt 2.0.2 2020-09-01 [1] CRAN (R 4.1.1)

modelr 0.1.8 2020-05-19 [1] CRAN (R 4.1.1)

munsell 0.5.0 2018-06-12 [1] CRAN (R 4.1.1)

mvtnorm * 1.1-3 2021-10-08 [1] CRAN (R 4.1.1)

nlme 3.1-152 2021-02-04 [2] CRAN (R 4.1.1)

nloptr 1.2.2.2 2020-07-02 [1] CRAN (R 4.1.1)

nnet 7.3-16 2021-05-03 [2] CRAN (R 4.1.1)

numDeriv 2016.8-1.1 2019-06-06 [1] CRAN (R 4.1.1)

pander 0.6.4 2021-06-13 [1] CRAN (R 4.1.2)

parallel 4.1.1 2021-08-10 [2] local

parameters * 0.16.0 2022-01-12 [1] CRAN (R 4.1.2)

pbivnorm 0.6.0 2015-01-23 [1] CRAN (R 4.1.1)

performance 0.8.0 2021-10-01 [1] CRAN (R 4.1.2)

pillar 1.6.4 2021-10-18 [1] CRAN (R 4.1.1)

pkgbuild 1.3.1 2021-12-20 [1] CRAN (R 4.1.2)

pkgconfig 2.0.3 2019-09-22 [1] CRAN (R 4.1.1)

pkgload 1.2.4 2021-11-30 [1] CRAN (R 4.1.3)

png 0.1-7 2013-12-03 [1] CRAN (R 4.1.1)

prettyunits 1.1.1 2020-01-24 [1] CRAN (R 4.1.1)

processx 3.5.2 2021-04-30 [1] CRAN (R 4.1.1)

ps 1.6.0 2021-02-28 [1] CRAN (R 4.1.1)

psych * 2.1.9 2021-09-22 [1] CRAN (R 4.1.1)

purrr * 0.3.4 2020-04-17 [1] CRAN (R 4.1.1)

R6 2.5.1 2021-08-19 [1] CRAN (R 4.1.1)

RColorBrewer 1.1-2 2014-12-07 [1] CRAN (R 4.1.1)

Rcpp 1.0.7 2021-07-07 [1] CRAN (R 4.1.1)

readr * 2.0.2 2021-09-27 [1] CRAN (R 4.1.1)

readxl 1.3.1 2019-03-13 [1] CRAN (R 4.1.1)

remotes 2.4.2 2021-11-30 [1] CRAN (R 4.1.3)

report * 0.4.0 2021-09-30 [1] CRAN (R 4.1.2)

reprex 2.0.1 2021-08-05 [1] CRAN (R 4.1.1)

rlang 1.0.2 2022-03-04 [1] CRAN (R 4.1.3)

rmarkdown 2.11 2021-09-14 [1] CRAN (R 4.1.1)

rpart 4.1-15 2019-04-12 [2] CRAN (R 4.1.1)

rprojroot 2.0.2 2020-11-15 [1] CRAN (R 4.1.2)

rstudioapi 0.13 2020-11-12 [1] CRAN (R 4.1.1)

rvest 1.0.2 2021-10-16 [1] CRAN (R 4.1.1)

sandwich * 3.0-1 2021-05-18 [1] CRAN (R 4.1.1)

sass 0.4.0 2021-05-12 [1] CRAN (R 4.1.2)

scales 1.1.1 2020-05-11 [1] CRAN (R 4.1.1)

sessioninfo 1.2.2 2021-12-06 [1] CRAN (R 4.1.3)

sjlabelled 1.1.8 2021-05-11 [1] CRAN (R 4.1.2)

sjmisc 2.8.9 2021-12-03 [1] CRAN (R 4.1.2)

sjPlot * 2.8.10 2021-11-26 [1] CRAN (R 4.1.2)

sjstats 0.18.1 2021-01-09 [1] CRAN (R 4.1.2)

splines 4.1.1 2021-08-10 [2] local

stats * 4.1.1 2021-08-10 [2] local

stats4 4.1.1 2021-08-10 [2] local

stringi 1.7.5 2021-10-04 [1] CRAN (R 4.1.1)

stringr * 1.4.0 2019-02-10 [1] CRAN (R 4.1.1)

survival 3.2-11 2021-04-26 [2] CRAN (R 4.1.1)

svglite 2.0.0 2021-02-20 [1] CRAN (R 4.1.1)

systemfonts 1.0.3 2021-10-13 [1] CRAN (R 4.1.1)

testthat 3.1.4 2022-04-26 [1] CRAN (R 4.1.3)

tibble * 3.1.5 2021-09-30 [1] CRAN (R 4.1.1)

tidyr * 1.1.4 2021-09-27 [1] CRAN (R 4.1.1)

tidyselect 1.1.1 2021-04-30 [1] CRAN (R 4.1.1)

tidyverse * 1.3.1 2021-04-15 [1] CRAN (R 4.1.1)

tmvnsim 1.0-2 2016-12-15 [1] CRAN (R 4.1.1)

tools 4.1.1 2021-08-10 [2] local

tzdb 0.1.2 2021-07-20 [1] CRAN (R 4.1.1)

usethis * 2.1.6 2022-05-25 [1] CRAN (R 4.1.3)

utf8 1.2.2 2021-07-24 [1] CRAN (R 4.1.1)

utils * 4.1.1 2021-08-10 [2] local

vctrs 0.3.8 2021-04-29 [1] CRAN (R 4.1.1)

viridisLite 0.4.0 2021-04-13 [1] CRAN (R 4.1.1)

webshot 0.5.2 2019-11-22 [1] CRAN (R 4.1.1)

withr 2.5.0 2022-03-03 [1] CRAN (R 4.1.3)

xfun 0.27 2021-10-18 [1] CRAN (R 4.1.1)

xml2 1.3.2 2020-04-23 [1] CRAN (R 4.1.1)

xtable 1.8-4 2019-04-21 [1] CRAN (R 4.1.2)

yaml 2.2.1 2020-02-01 [1] CRAN (R 4.1.1)

zoo 1.8-9 2021-03-09 [1] CRAN (R 4.1.1)

---------------------------------------------------------------------------------------------------------------------

## Map of Study Sites


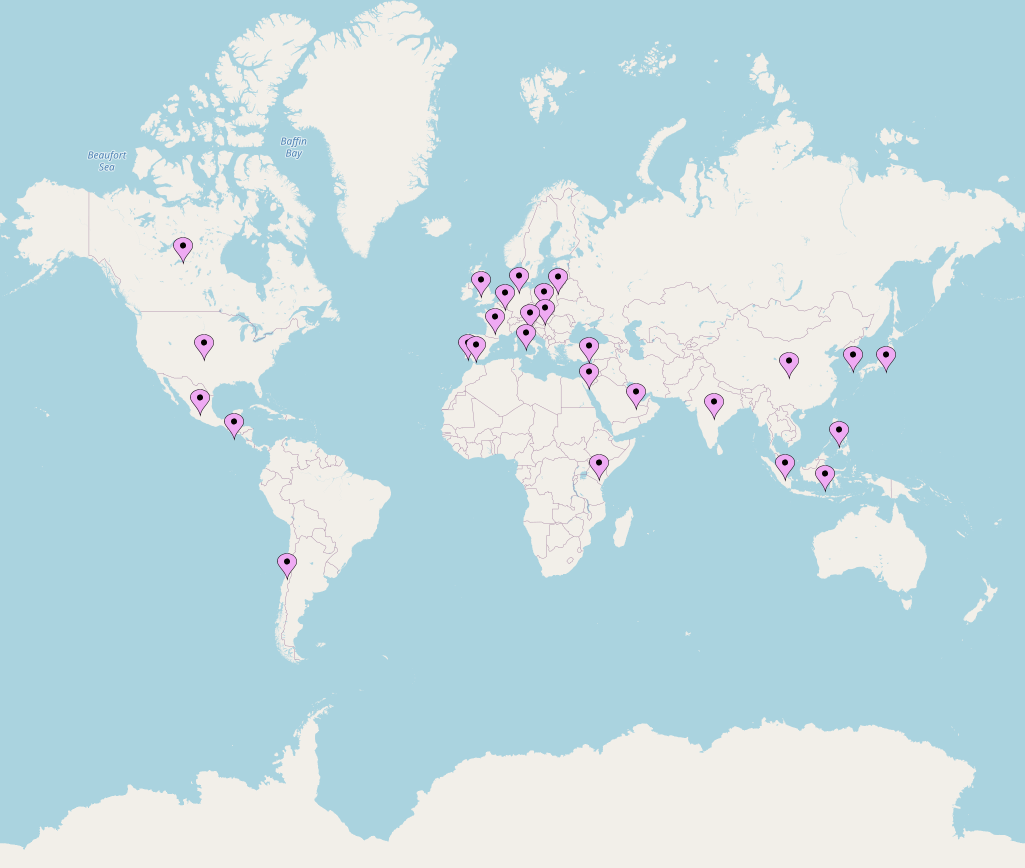


**Figure S1.** Map of countries (purple pins) that were included in the study. See a list of study sites in Table S3. This map was created by the authors using www.mapcustomizer.com.

## Analyses Supporting Main Text

### Traditionalism-precautions correlations and density distributions by study site

In the caption for Figure 2 in the main text, we noted that study-site-specific labeled regression plots and density distribution plots for traditionalism and COVID-19 health precautions could be found in the Supplement. Here, we provide those plots (Figures S2-S4).


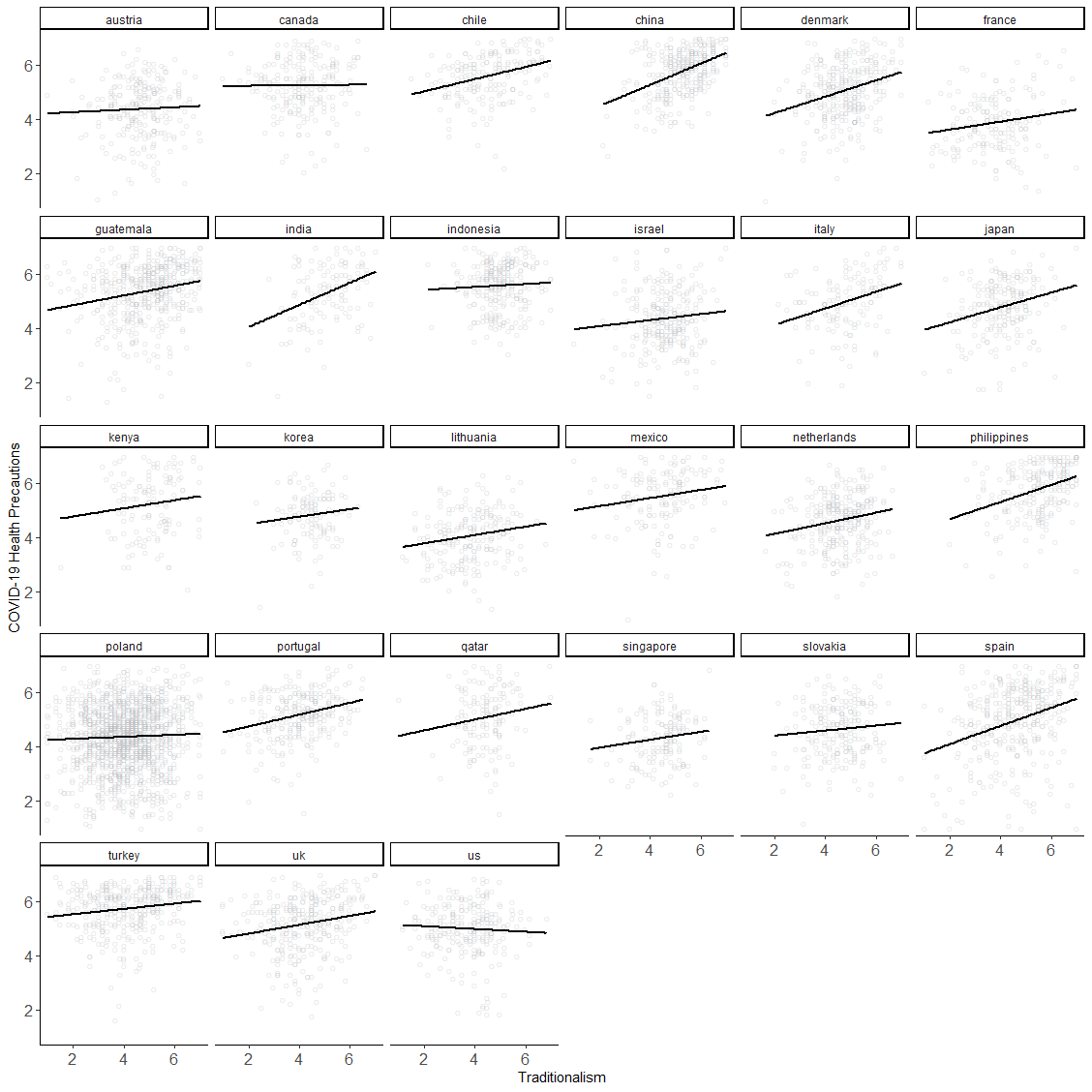


**Figure S2.** Regression lines plotting zero-order correlations between traditionalism and COVID-19 health precautions at each study site individually. Beans show raw data points. Compare to Figure 2 in the main text.


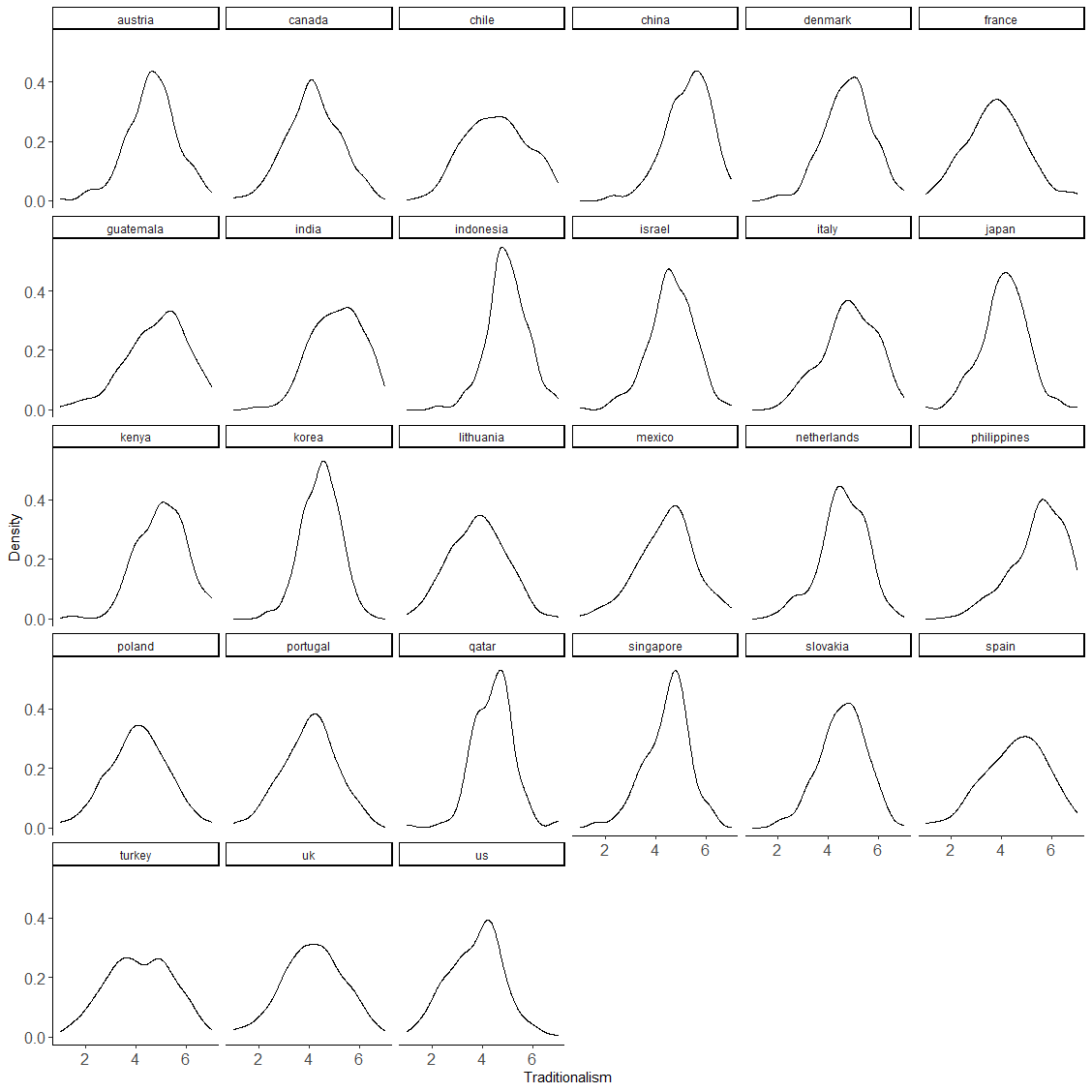


**Figure S3.** Raw density distributions of traditionalism composite across each study site individually. Compare to Figure 2 in the main text.

**Figure S4.** Raw density distributions of COVID-19 health precautions composite across each study site individually. Compare to Figure 2 in the main text.


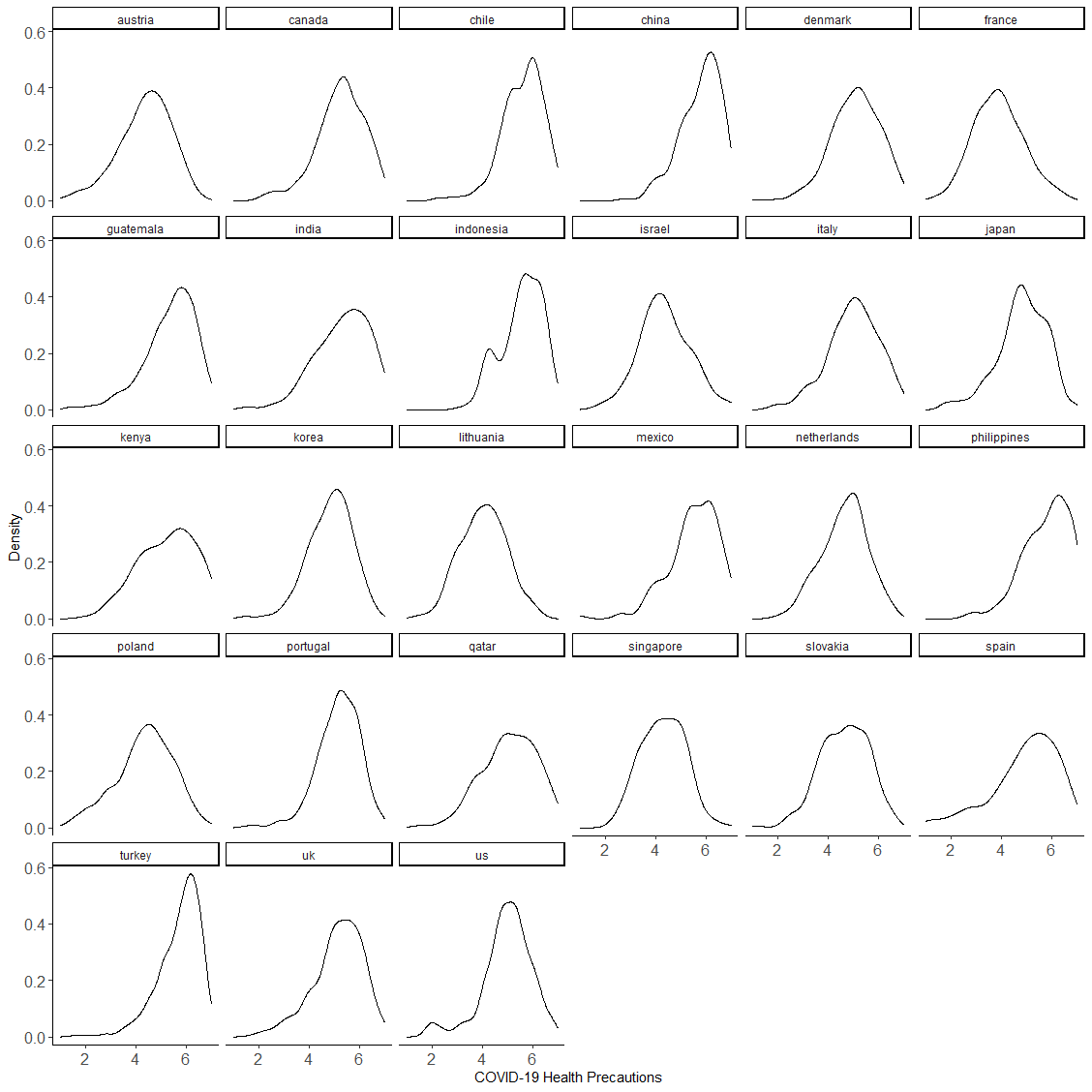


**Figure S4.** Raw density distributions of COVID-19 health precautions composite across each study site individually. Compare to Figure 2 in the main text.

### Traditionalism-precautions relationship adjusting for covariates

In the main text, we noted that the bivariate correlation between COVID-19 health precautions and traditionalism is robust to the inclusion of the following demographic controls and COVID-19 related covariates: age; gender; education; relative income, perceived COVID prevalence in participants’ local communities; the population density of those communities; whether participants’ jobs required that they leave home; and whether participants had certain pre-existing medical conditions that may put them at higher risk for severe disease. To test this, we conducted a random-effects, restricted maximum likelihood meta-analysis in which each study site was treated as a separate sample. We examined the semi-partial correlation between traditionalism and health precautions after adjusting for the effects of those seven variables in multiple linear regressions where health precautions were regressed on traditionalism and the seven covariates. Covariates were identical across study sites. As seen in Figure S5, adjusting for these demographic and COVID-related controls did not conceptually change the results.


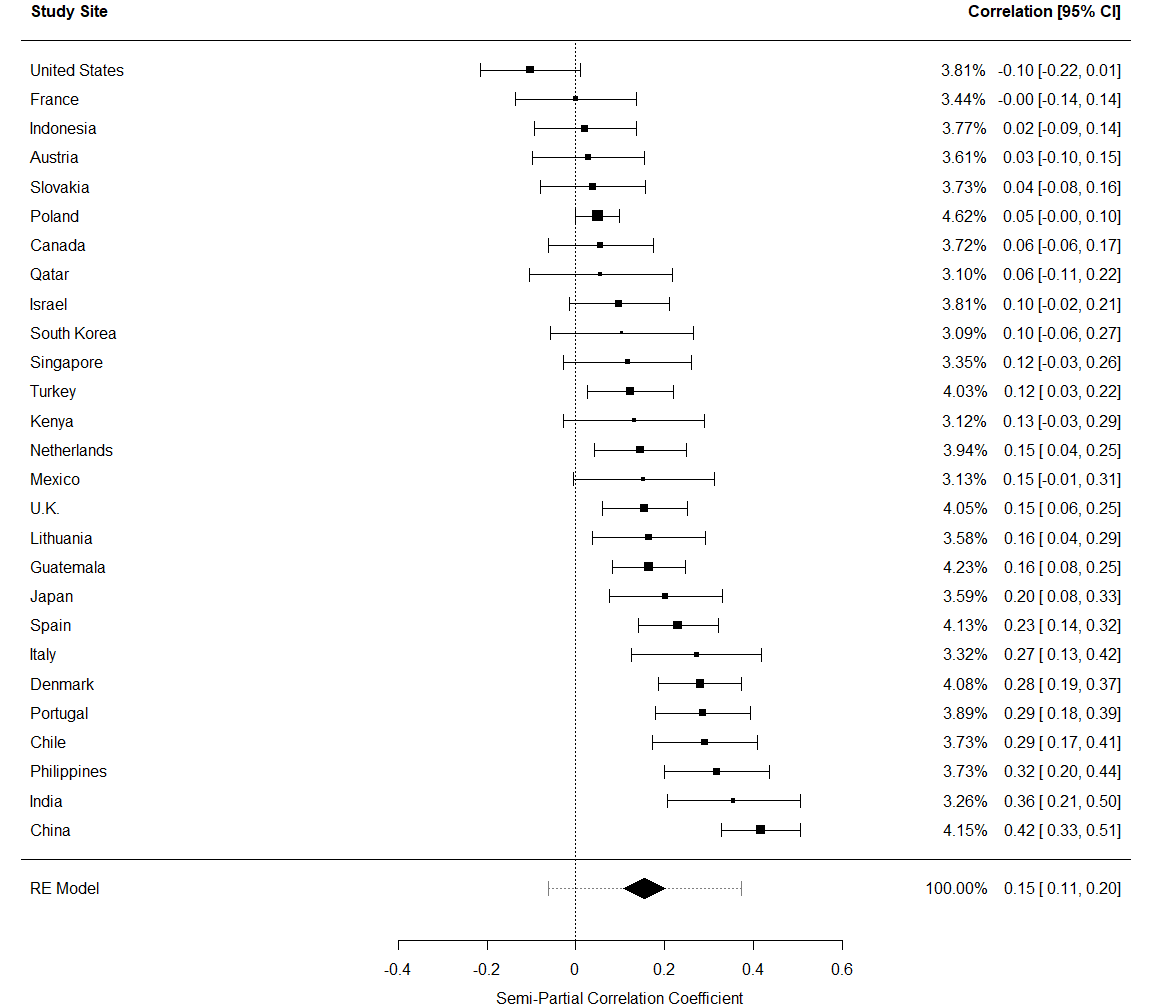
We also noted in the main text that the same set of demographic and COVID-related covariates did not conceptually change the precautions-traditionalism relationship after adjusting for the effects of the identified suppressor variables. To test this, we ran a meta-analysis similar to the one conducted for Figure S5, however this time also including the five suppressor variables as covariates in the regression models. As seen in Figure S6, adjusting for these demographic and COVID-related controls did not conceptually change the results.

**Figure S5.** Results of random-effects meta-analysis examining the relationship between traditionalism and COVID-19 health precautions after adjusting for demographic variables and COVID-relevant covariates. See Figure 1 in the main text for a description of how to interpret the forest plot.


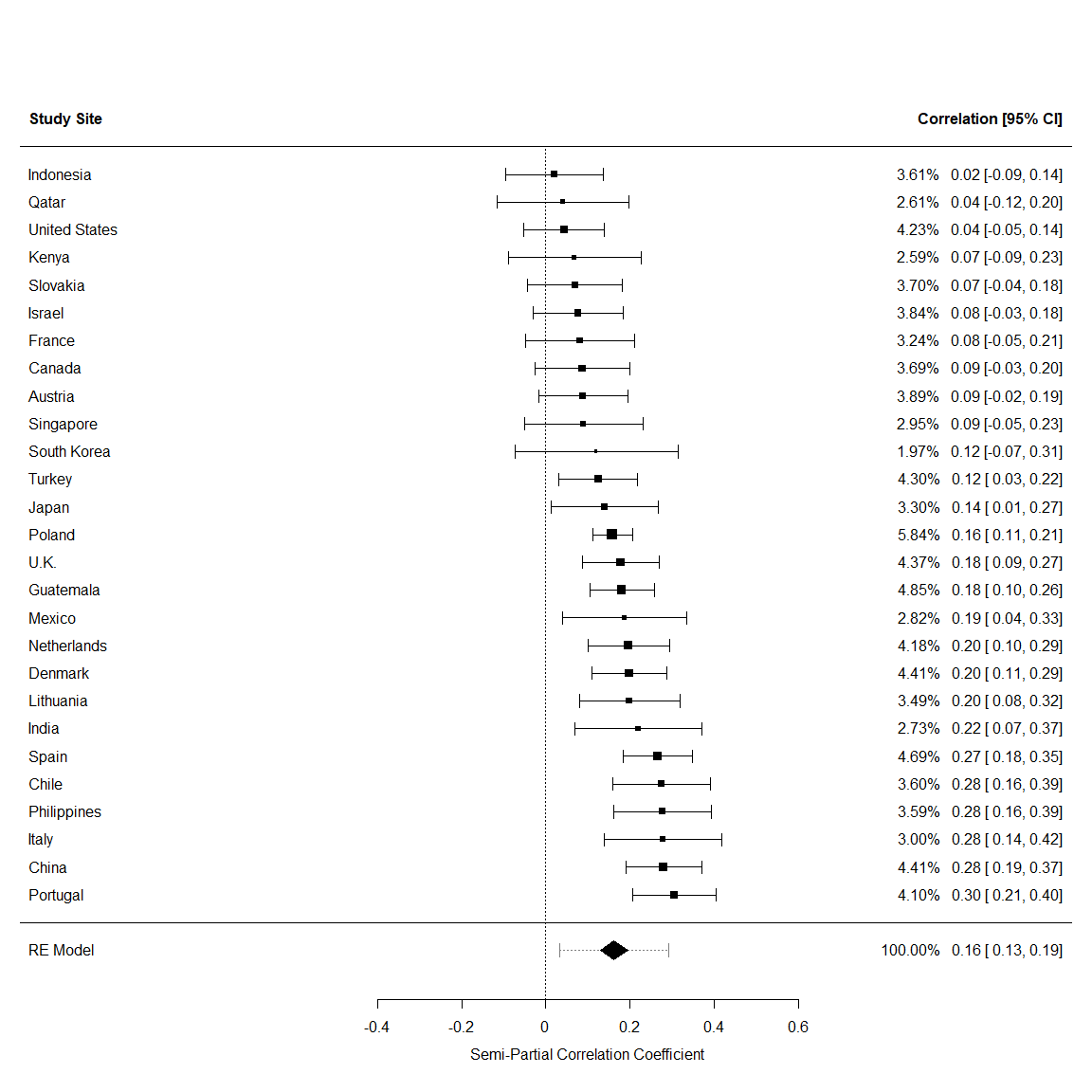


**Figure S6.** Results of random-effects meta-analysis examining the relationship between traditionalism and COVID-19 health precautions after adjusting for suppressor variables, as well as demographic variables and COVID-relevant covariates. See Figure 1 in the main text for a description of how to interpret the forest plot.

### Precautions-traditionalism relationship disattenuated for unreliability

Given variation in scale reliability across study sites (see Table S8), we conducted meta-analyses on the traditionalism-precautions relationship that disattenuated for unreliability. In the main text, we noted that disattenuating for unreliability did not conceptually affect the results. First, we conducted a meta-analysis on the zero-order correlation between traditionalism and COVID-19 health precautions that disattenuated for unreliability; results are shown in Figure S7.


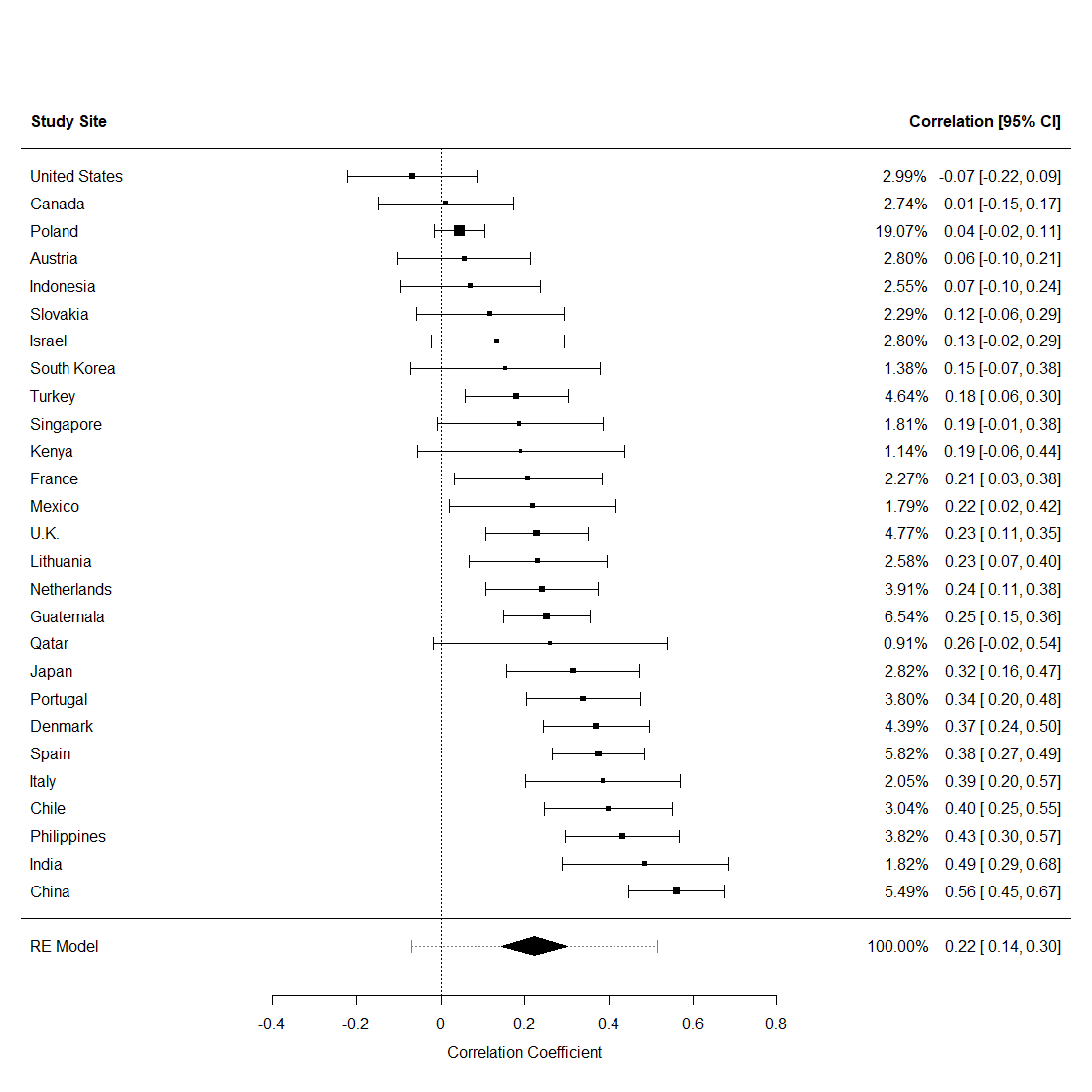


**Figure S7.** Results of random-effects meta-analysis examining the relationship between traditionalism and COVID-19 health precautions after disattenuating for scale unreliability across study sites. See Figure 1 in the main text for a description of how to interpret the forest plot.

Second, we conducted a meta-analysis examining the semi-partial correlation between COVID-19 health precautions and traditionalism after adjusting for the effects of the five suppressor variables, while also disattenuating for scale unreliability (see Figure S8). In both cases, findings reported in the main text were not conceptually changed by disattenuating for unreliability.


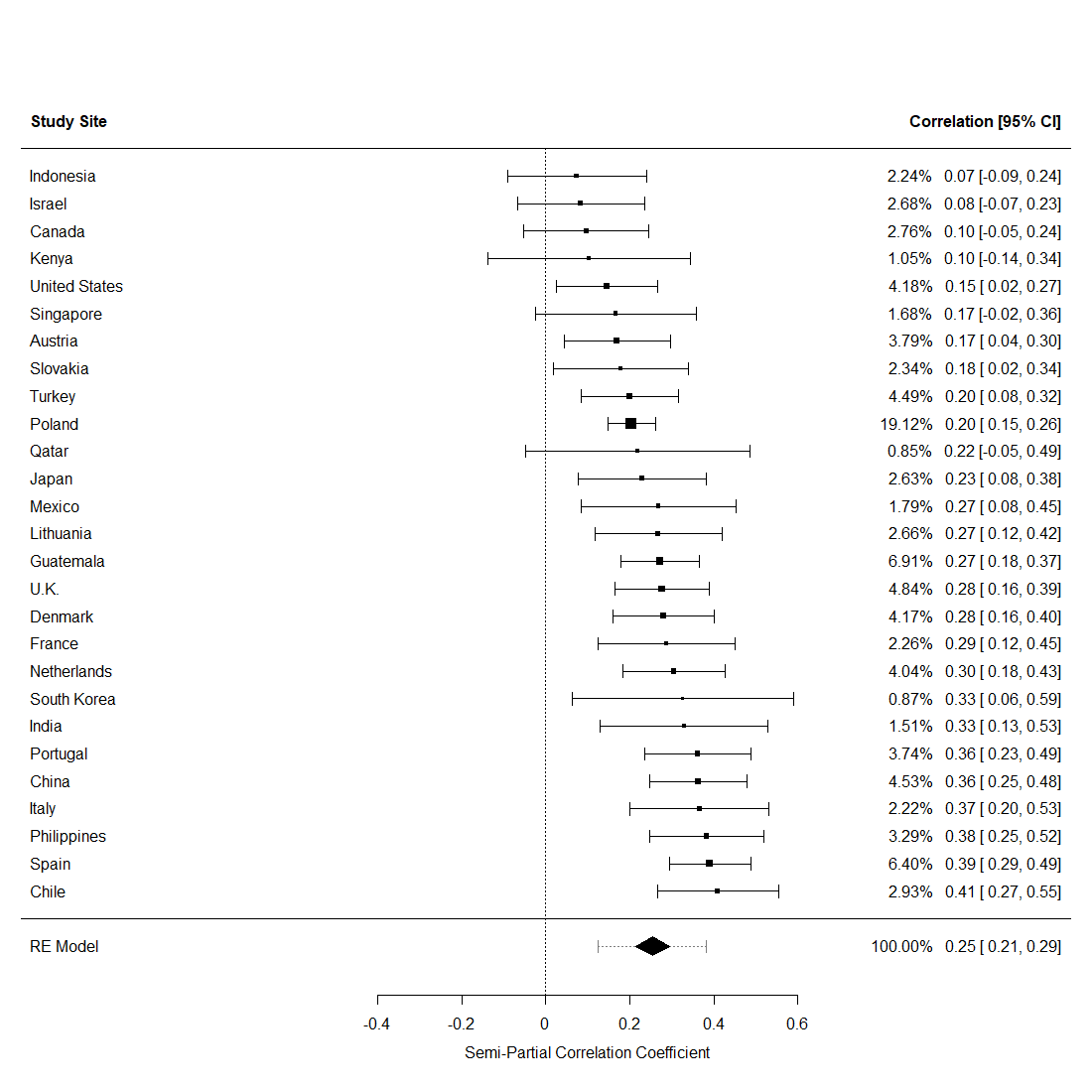


**Figure S8.** Results of random-effects meta-analysis examining the relationship between traditionalism and COVID-19 health precautions after adjusting for the five suppressor variables, and disattenuating for scale unreliability across study sites. See Figure 1 in the main text for a description of how to interpret the forest plot.

### Effects of COVID-19 prevalence on study estimates of traditionalism-precautions relationship

In the main text, we noted that country-specific rates of COVID-19 prevalence did not explain any meaningful variance in effect sizes between study sites. To test this, we conducted two meta-regressions using two different measures of national COVID-19 prevalence. First, we calculated national average daily confirmed cases per million people over the specific period of data collection at each study site, obtained from Our World in Data^30^. Second, we calculated national total cumulative cases per million people since the start of the pandemic at the end of the specific period of data collection at each study site, obtained from the same source. For both meta-regressions, we tested whether the two COVID-19 prevalence metrics moderated the zero-order correlation between traditionalism and COVID-19 health precautions, and thus whether they accounted for any of the variance in heterogeneity in effect sizes across study sites. Neither average daily cases (QM = .51, *p* = .474, R^2^ = .00) nor cumulative cases (QM = 1.67, *p* = .198, R^2^ = .04) moderated the precautions-traditionalism relationship.

### Identifying suppressor variables

The study included seven variables (see Methods section in main text for details) that were tested for possible suppressive effects on the health precautions-traditionalism relationship. As stated in the main text, we conducted mediation analyses to test for suppression across the pooled sample, where suppression was indicated by the presence of negative indirect effects (in contrast to the positive indirect effects that characterize mediation). See the Results section in the main text for further details on the statistical procedure. In the main text, we stated that we identified five suppressor variables in the pooled sample using this procedure, shown here in Table S1.

**Table S1.** Results of mediation analyses testing for suppression of the precautions-traditionalism relationship using a pooled sample across all 27 study sites. Each of the seven candidates were tested separately, and five variables were identified as suppressors. Note that coefficients are unstandardized betas (all seven candidate variables were measured on 1-to-7 Likert-type scales).

| **Suppressor Candidate** | **Indirect effect** | **Lower 95% CI** | **Upper 95% CI** |
| --- | --- | --- | --- |
| SDO | -.018 | -.022 | -.013 |
| Trust in scientists | -.015 | -.023 | -.008 |
| Concern over economy | .009 | .006 | .012 |
| Concern over personal liberties | .000 | -.001 | .001 |
| Liberties-public health tradeoffs | -.014 | -.017 | -.010 |
| Economy-public health tradeoffs | -.022 | -.027 | -.018 |
| Traditions-public health tradeoffs | -.037 | -.043 | -.031 |

Next, we assessed the combined suppressive effects of the five variables at each study site individually, in order to understand whether those suppressors were acting in some socio-political contexts but not others. To test for the combined effects of the suppressors, all five variables were simultaneously entered in an individual mediation analysis for each study site using the R package *lavaan*^13^. As seen in Table S2, the effects of the suppressor variables varied substantially across study sites. Indeed, at two of the study sites, the combined effects of those five variables actually resulted in partial mediation—not suppression—of the health precautions-traditionalism relationship.


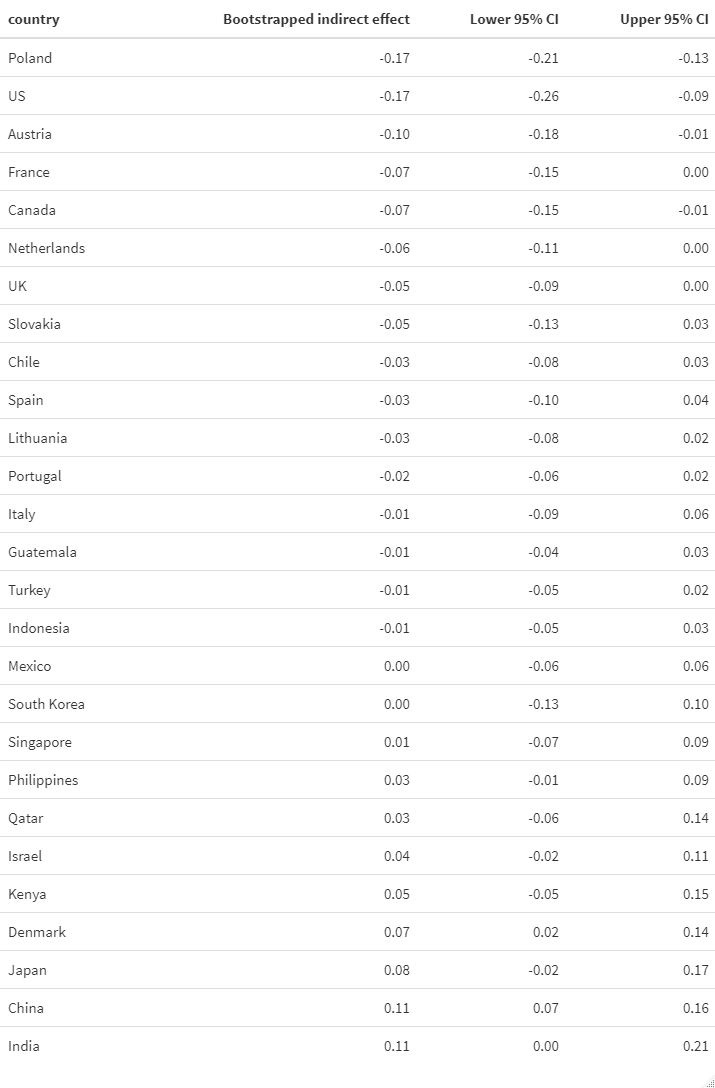


**Table S2.** Results of mediation analyses testing for the combined effects of the five suppressors on the health precautions-traditionalism relationship at each of the 27 study sites.

### Precautions subscale interaction adjusting for covariates, suppressor variables, and planning items

In the main text, we report that the interaction between traditionalism and external-versus-internal precautions subscale (see Figure 4) was robust to the inclusion of the seven previously used demographic and COVID-19-related covariates, as well as the five suppressor variables. Here, we report those results.

When controlling for the demographic and COVID-19-related covariates, there was an interaction between health precautions subscale and traditionalism (*B* = .15, SE = .01, *t*(7,274) = 12.39, *p* < .001). A simple slopes analysis revealed that the correlation between traditionalism and internal-facing precautions (*B* = .27, SE = .01, *t*(7,274) = 21.66, *p* < .001) was about twice as strong as the correlation between traditionalism and external-facing precautions (*B* = .11, SE = .01, *t*(7,274) = 9.24, *p* < .001).

When controlling for the five suppressor variables, there was an interaction between health precautions subscale and traditionalism (*B* = .16, SE = .01, *t*(7,122) = 12.50, *p* < .001). A simple slopes analysis revealed that the correlation between traditionalism and internal-facing precautions (*B* = .33, SE = .01, *t*(7,122) = 26.95, *p* < .001) was about twice as strong as the correlation between traditionalism and external-facing precautions (*B* = .17, SE = .01, *t*(7,122) = 14.11, *p* < .001).

Finally, when controlling for both the suppressor and demographic variables, there was an interaction between health precautions subscale and traditionalism (*B* = .16, SE = .01, *t*(6,882) = 12.26, *p* < .001). A simple slopes analysis revealed that the correlation between traditionalism and internal-facing precautions (*B* = .30, SE = .01, *t*(6,882) = 24.73, *p* < .001) was about twice as strong as the correlation between traditionalism and external-facing precautions (*B* = .14, SE = .01, *t*(6,882) = 11.82, *p* < .001).

In addition, in the main text, we report that the interaction between health precautions subscale and traditionalism does not appear to be confounded by the fact that the internal-facing precautions subscale has more items concerning planning precautions compared to the external-facing precautions (thus providing a plausible alternative framing to the distinction between the two subscales that is driving the reported interaction). To address this possibility, we computed a modified internal-facing precautions composite that excluded all planning-related precautions (we removed the first three items listed under the internal-facing precautions header, see page S2). We then re-conducted the analyses reported in Figure 4 in the main text, however using the modified internal-facing precautions composite in place of the full composite. Using the planning-less composite did not conceptually affect the results, suggesting that the interaction is not being driven by differences between planning versus non-planning precautions. Specifically, there was an interaction between health precautions subscale and traditionalism (*B* = .18, SE = .01, *t*(7,535) = 13.96, *p* < .001). A simple slopes analysis revealed that the correlation between traditionalism and planning-less internal-facing precautions (*B* = .32, SE = .01, *t*(7,535) = 24.66, *p* < .001) was about twice as strong as the correlation between traditionalism and external-facing precautions (*B* = .14, SE = .01, *t*(7,535) = 10.69, *p* < .001).

### Summary statistics and other information by study site

Table S3, below, presents a list of study sites, study-site specific Ns, as well as information on survey languages, recruitment procedures, and participant demographics for each study site. In the main text we report excluding participants on the basis of minimum completeness and correct answers to attention checks. Across all the study sites, 11,983 participants at least started the survey. We excluded 4,139 participants based on the above criteria, to arrive at a final sample size of 7,844. This relatively high attrition rate is unsurprising given that, at a majority of study sites, participants were uncompensated volunteers.

| **Study Site** | **Survey Language** | **Population** | **Recruitment Method** | **Compensation** | **N** | **Mean (SD) Age** | **% Women** | **Mean (SD) Traditionalism Factor Composite** | **Mean (SD) COVID-19 Health Precautions Composite** | **Average daily confirmed COVID-19 cases per million people over data collection period** |
| --- | --- | --- | --- | --- | --- | --- | --- | --- | --- | --- |
| Austria | German | Students; general population | Social media; classrooms | Volunteer | 244 | 34.69 (13.28) | 84 | 4.64 (1.00) | 4.38 (1.04) | 205.84 |
| Canada | English | Students | Subject pools | Course credit | 221 | 19.34 (2.40) | 77 | 4.16 (1.04) | 5.25 (.96) | 109.18 |
| Chile | Spanish | Students; general population | Social media; classrooms | Volunteer | 195 | 31.91 (12.71) | 67 | 4.56 (1.21) | 5.61 (.83) | 359.77 |
| China | Mandarin Chinese | General population | Online workers (Weidiaocha) | CNY ¥6 | 317 | 25.27 (6.21) | 55 | 5.30 (.91) | 5.78 (.81) | .02 |
| Denmark | Danish | General population | Online workers (YouGov) | 75 YouGov points | 307 | 50.11 (18.23) | 50 | 4.77 (.96) | 5.07 (.97) | 233.77 |
| France | French | Students; general population | Social media; classrooms | Volunteer | 176 | 29.80 (13.29) | 66 | 3.78 (1.18) | 3.88 (.99) | 52.18 |
| Guatemala | Spanish | General population | Social media | Volunteer | 457 | 39.65 (12.67) | 80 | 4.83 (1.22) | 5.36 (1.03) | 41.00 |
| India | English | Students | Classrooms | Volunteer | 118 | 28.33 (9.01) | 62 | 5.12 (.99) | 5.33 (1.06) | 8.60 |
| Indonesia | Indonesian | Students; general population | Classrooms; social media | Volunteer | 257 | 31.10 (9.97) | 76 | 5.00 (.80) | 5.57 (.82) | 32.02 |
| Israel | Hebrew | Students | Subject pools | Course credit | 267 | 22.49 (2.44) | 51 | 4.63 (.91) | 4.37 (1.01) | 609.52 |
| Italy | Italian | General population | Social media | Volunteer | 135 | 35.31 (15.50) | 61 | 4.83 (1.05) | 4.99 (1.02) | 235.94 |
| Japan | Japanese | Students | Subject pools; classrooms | Course credit or volunteer | 231 | 22.36 (4.36) | 46 | 4.10 (.92) | 4.81 (.98) | 19.40 |
| Kenya | English | Students; general population | Classrooms; snowball recruitment | Course credit or volunteer | 133 | 23.60 (4.91) | 50 | 5.04 (.96) | 5.22 (1.12) | 8.60 |
| South Korea | Korean | Students | Subject pools; classrooms | Course credit or volunteer | 167 | 23.25 (3.92) | 63 | 4.47 (.73) | 4.86 (.87) | 11.05 |
| Lithuania | Lithuanian | Students; general population | Social media; classrooms | Volunteer | 211 | 28.03 (10.33) | 80 | 3.81 (1.08) | 4.05 (.92) | 213.78 |
| Mexico | Spanish | Students; general population | Social media; classrooms | Volunteer | 153 | 28.75 (10.97) | 65 | 4.44 (1.13) | 5.51 (1.00) | 29.02 |
| Netherlands | Dutch | General population | Online workers (Prolific Academic) | € 1.10 | 300 | 29.58 (10.22) | 41 | 4.56 (.92) | 4.63 (.92) | 340.18 |
| Philippines | English | Students; general population | Social media | Volunteer | 229 | 21.17 (3.64) | 75 | 5.44 (1.05) | 5.76 (.92) | 67.20 |
| Poland | Polish | Students; general population | Social media; classrooms | Volunteer | 1,665 | 22.98 (7.54) | 74 | 4.08 (1.17) | 4.35 (1.15) | 240.09 |
| Portugal | Portuguese | General population | Subject pools | Raffle (5 prizes worth € 10.00) | 264 | 27.58 (8.82) | 76 | 4.03 (1.08) | 5.18 (.85) | 53.41 |
| Qatar | Arabic | Students; general population | Social media; classrooms | Volunteer | 146 | 24.23 (6.84) | 82 | 4.44 (.81) | 5.08 (1.08) | 279.66 |
| Singapore | English | Students | Subject pools | Course credit | 155 | 21.58 (2.03) | 78 | 4.46 (.88) | 4.31 (.87) | 2.77 |
| Slovakia | Slovak | Students | classrooms | Volunteer | 222 | 21.90 (3.89) | 77 | 4.56 (.92) | 4.63 (.96) | 389.92 |
| Spain | Spanish | General population | Social media | Volunteer | 365 | 40.15 (13.74) | 79 | 4.56 (1.24) | 4.94 (1.32) | 562.56 |
| Turkey | Turkish | Students; general population | Social media; classrooms | Volunteer | 352 | 31.71 (16.28) | 77 | 4.15 (1.28) | 5.72 (.84) | 287.61 |
| U.K. | English | General population | Online workers (Prolific Academic) | £0.82 | 316 | 36.81 (13.87) | 70 | 4.16 (1.21) | 5.15 (.99) | 340.47 |
| U.S. | English | General population | Social media | Volunteer | 241 | 33.08 (18.91) | 83 | 3.74 (1.06) | 4.98 (.98) | 305.59 |
| Pooled Sample | - | - | - | - | 7,844 | 28.91 (12.95) | 70 | 4.44 (1.16) | 4.90 (1.14) | 186.64 |

**Table S3.** Summary statistics and other information by study site

### COVID-19 health precautions scale development

In the main text, we report a 12-item composite scale used to measure COVID-19 health precautions, as well as two-subscales—external-facing health precautions, and internal-facing health precautions. Here, we provide details on the scale development procedures and factor analyses used to produce these composites.

Based on a measure of COVID-19 precautions that we used in previous research^31^, participants were asked 15 questions concerning precautionary behaviors in response to COVID-19. Most items inquired about health behaviors, including the frequency of mask wearing, hand washing, social distancing, and disinfecting, and the importance to the participant of stocking up on supplies such as hand sanitizer and household disinfectants. Participants were also asked the extent to which they were following local lockdown restrictions, and whether they had been careful to physically distance from people outside their household. In addition to health behaviors, participants were also asked about religious precautions undertaken to prevent COVID-19 infection. Items were rated on 7-point scales, either from “never” to “as often as possible”, or from “not important at all”, to “extremely important”. See pages S1-S2 for full measures.

We ran an exploratory factor analysis on the pooled sample across all study sites to determine the structure of COVID-19 precautions. First, we used the Kaieser-Meyer-Olkin (KMO) and Bartlett’s test to determine whether these items were suitable for structure detection. The KMO test suggested that the strength of the relationships among the variables was high (KMO = .89), and Bartlett’s test was significant (χ2 (105) = 48,012.74, p < .001), suggesting that the use of factor analysis was appropriate.

The R package *parameters*^22^ was then used to determine how many factors to extract. There was the most agreement between methods for a three-factor solution (see Figure S9).


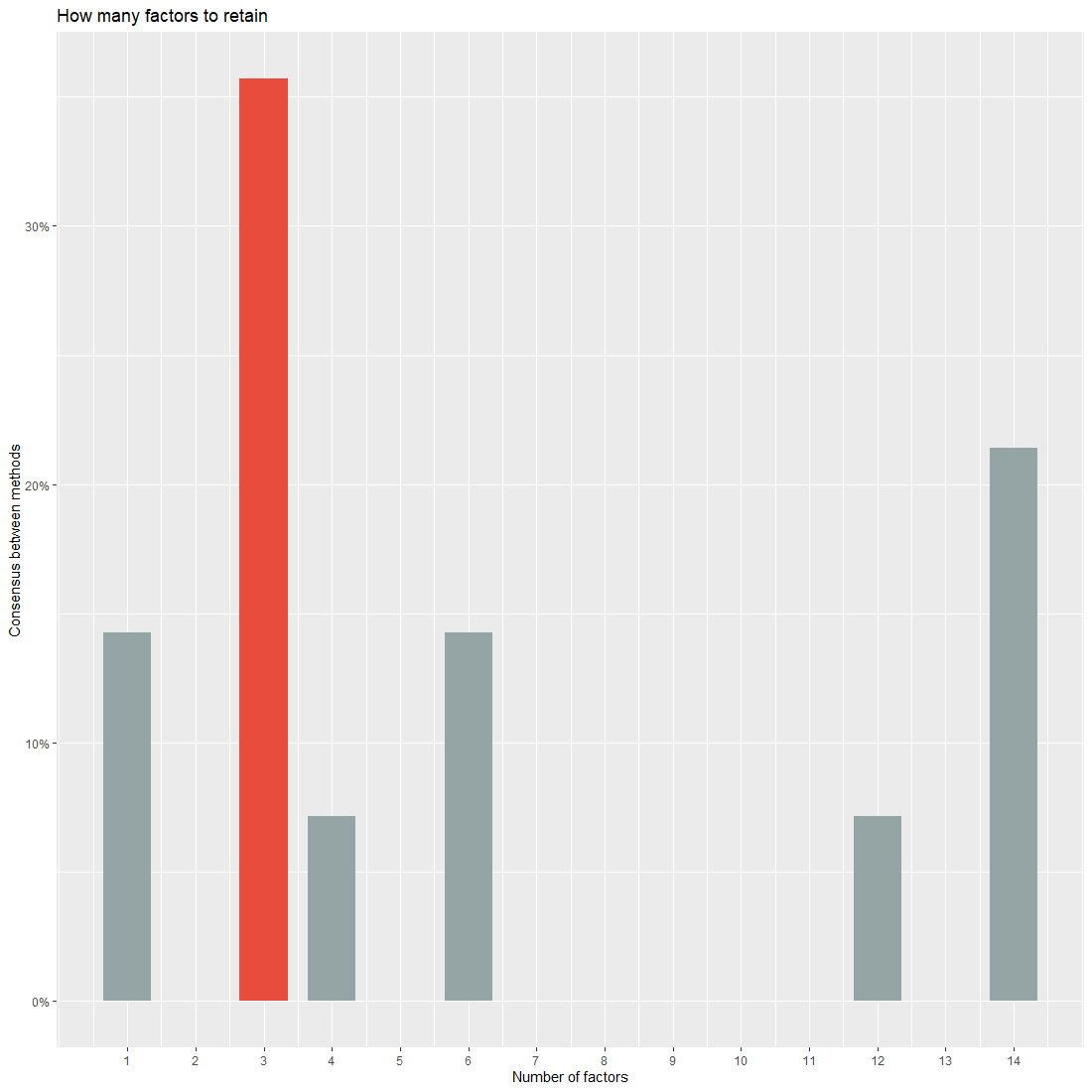
A factor analysis was then conducted with minimum residual extraction, promax rotation, and a Pearson’s correlation matrix. Three factors were extracted. The three factor values had sums of squared loadings of 3.94, 2.86, and 1.76, and explained 26%, 19%, and 12% of the variance, respectively. When extracted, these three factors were conceptually coherent (see Table S4). For each factor, items with factor loadings greater than .40 were averaged together, producing the composite measures used in analyses. We labeled these factor composites as follows: *internal-facing or non-interpersonal health precautions* (e.g., washing hands), *external-facing or interpersonal health precautions* (e.g., observing mask wearing and social distancing), and *religious precautions* (e.g., engaging in prayer). The internal-facing and external-facing composites were largely reliable across study sites, although there was cross-society variation with low alphas in some countries; see Table S5 for study site-specific reliability tests for each composite. Note that we reserve the analyses of the religious precautions composite for a separate project, hence they are not included here.

**Figure S9.** Graphical representation showing agreement between different methods for determining the number of factors to retain.


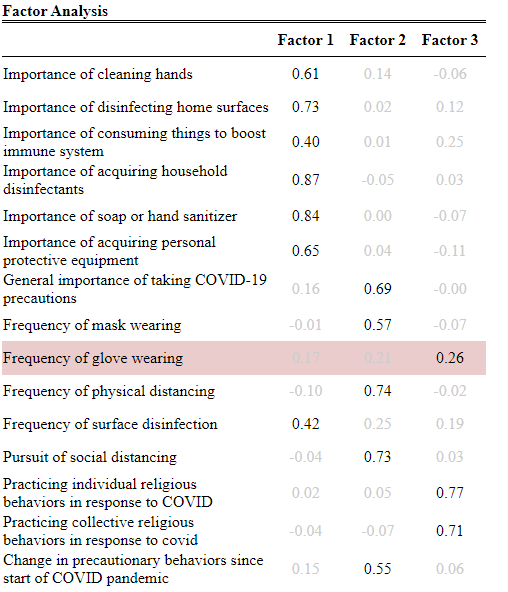


**Table S4.** Factor loadings from an exploratory factor analysis on COVID-19 precautions items.


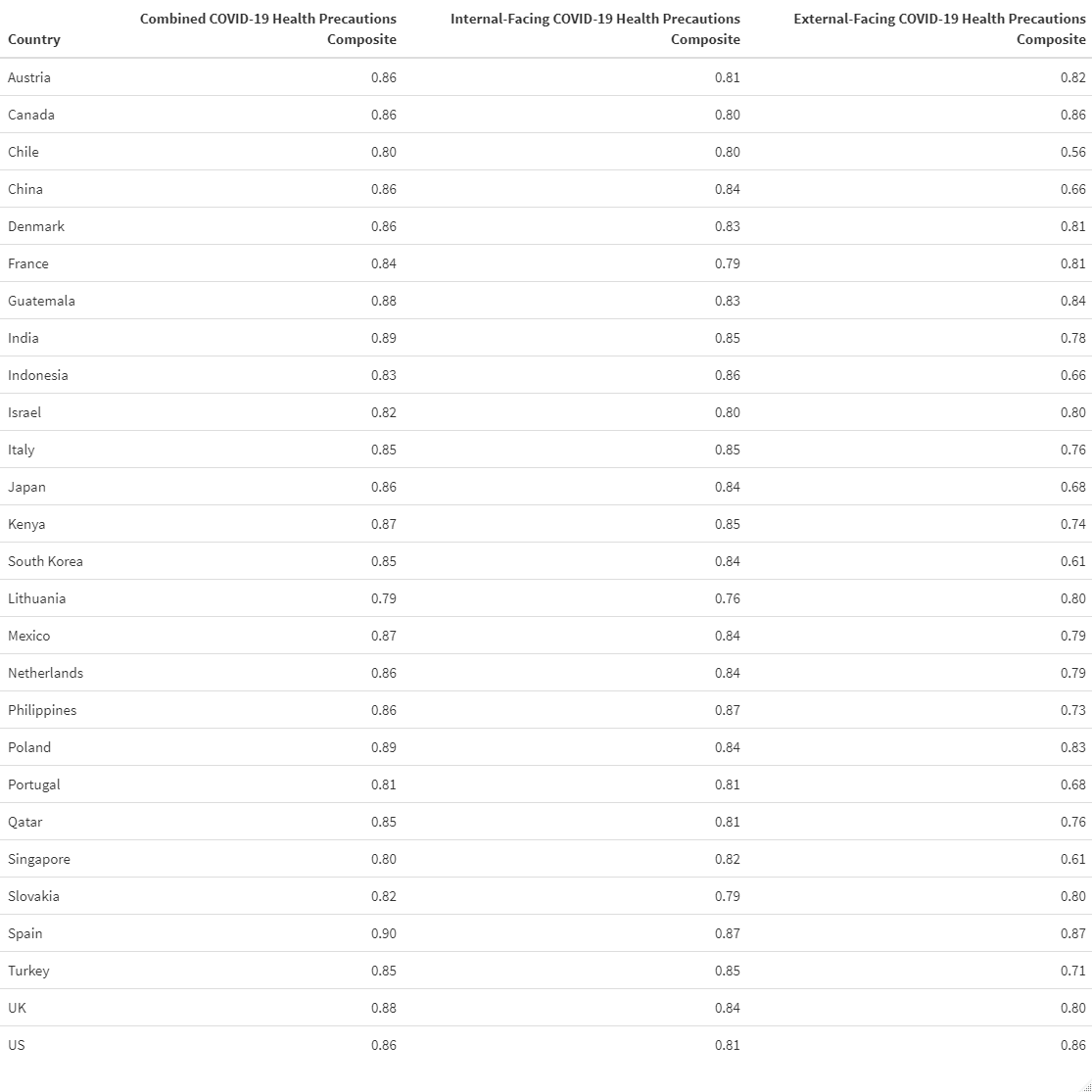


**Table S5.** Cronbach’s alphas for the combined health precautions composite, and the two internal-facing and external-facing subscale composites by study site.

We then fit a random-intercepts linear mixed model (estimated using REML) to examine the relationship between the internal-facing and external-facing precaution composites. The model included country as random effect. The two composites were highly correlated (β = .50, 95% CI [.48, .52), t(7,837) = 56.95, *p* < .001). Given this strong correlation, and for ease of interpretability in the analyses presented in the main text, we created an overall COVID-19 health precautions composite comprised of the raw average of all the items in both the internal-facing and external-facing composites. This combined composite was reliable across study sites (see Table S5), suggesting that these items cohere together. Where main text analyses were presented with the single composite factor (see Figures 1, 2, and 3), results did not conceptually change when using either of the two precautions subscales instead (see Figure S10-S13), although effect sizes were lower for the external-facing subscale, consistent with the interaction reported in Figure 4 in the main text.


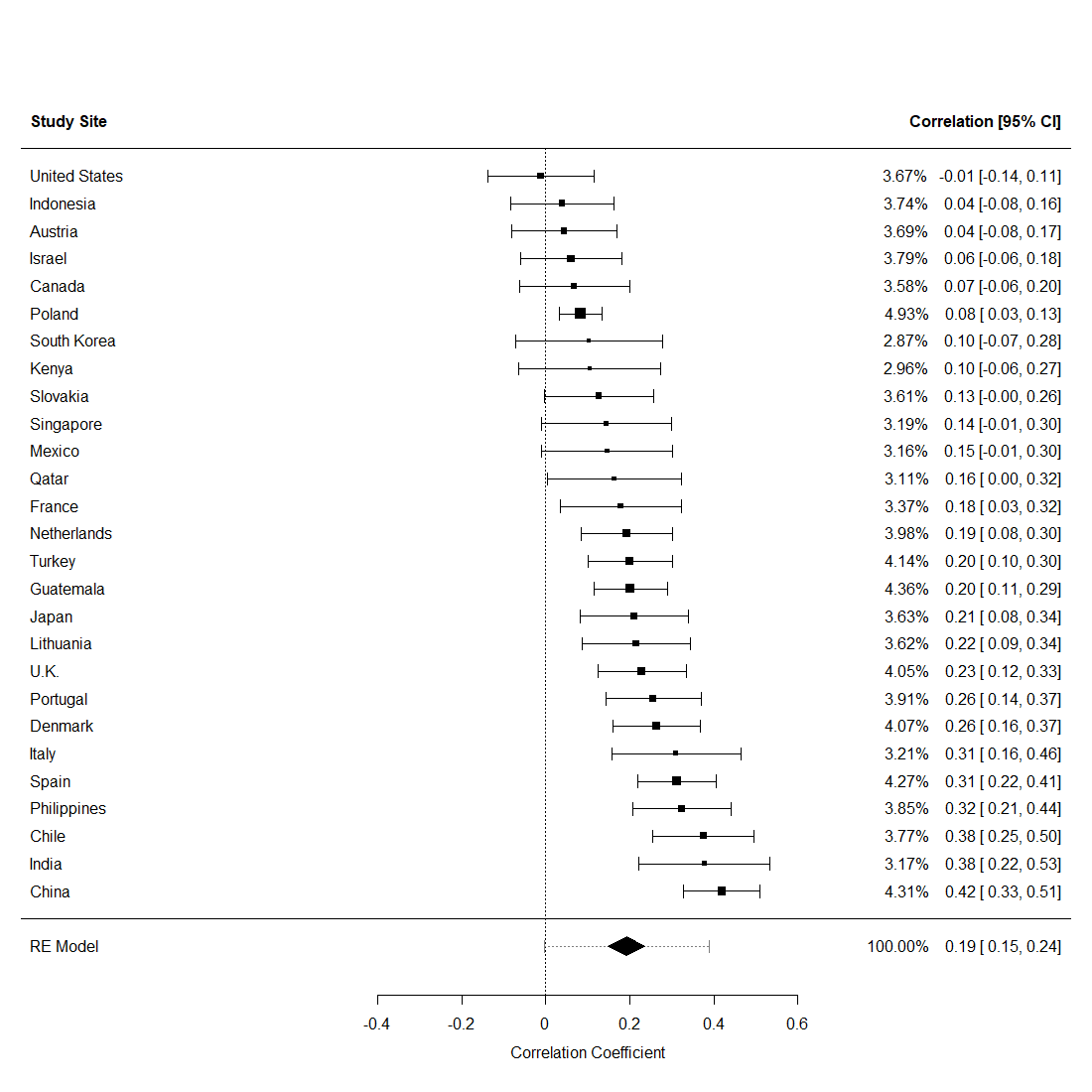


**Figure S10.** Results of random-effects meta-analysis examining the *zero-order correlation* between traditionalism and the *internal-facing precautions composite*. Compare to Figure 1 in the main text.


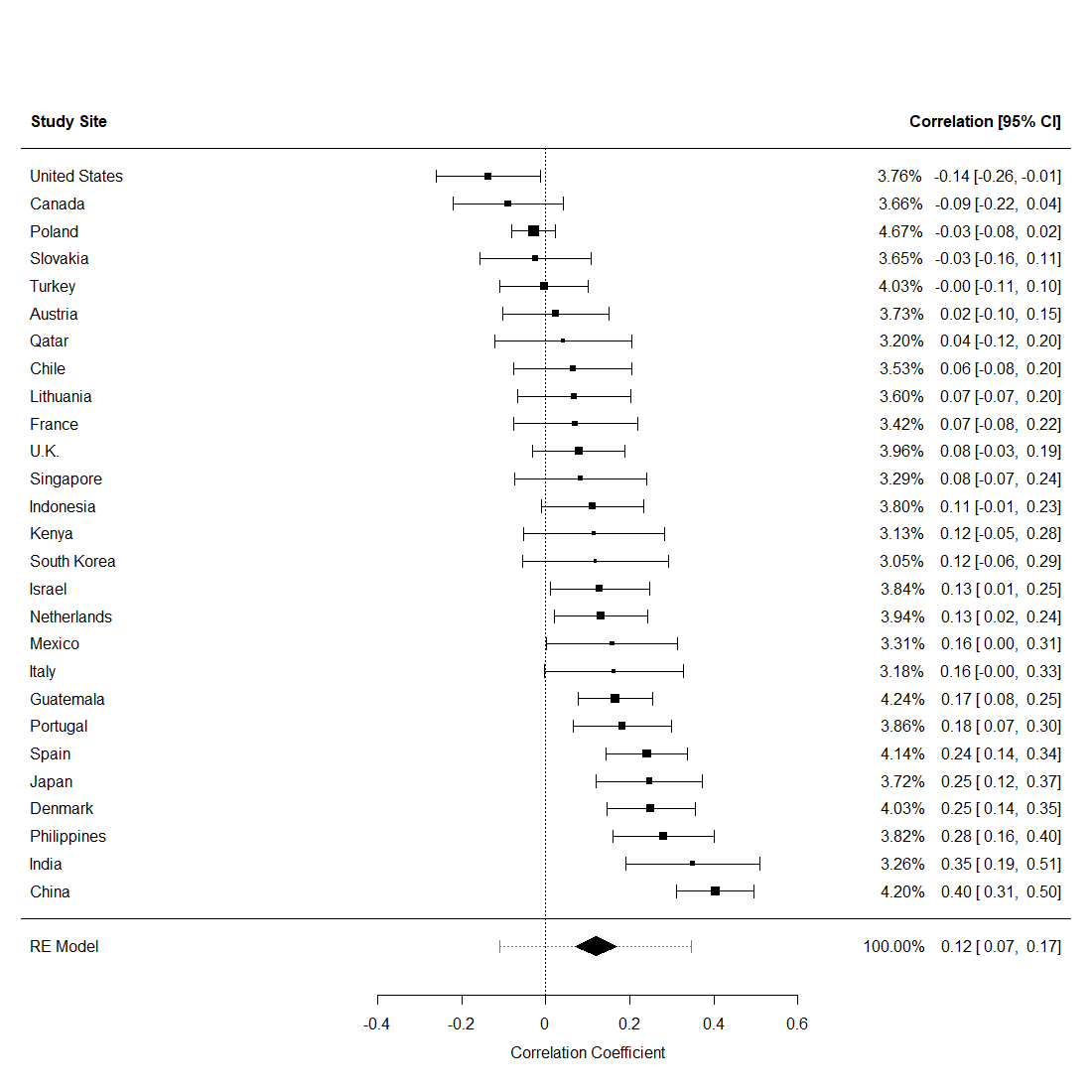


**Figure S11.** Results of random-effects meta-analysis examining the *zero-order correlation* between traditionalism and the *external-facing precautions composite*. Compare to Figure 1 in the main text.

**Figure S12.** Results of random-effects meta-analysis examining the *semi-partial correlation* between traditionalism and the *internal-facing precautions composite* after *adjusting for the effects of the five suppressor variables*. Compare to Figure 3 in the main text.


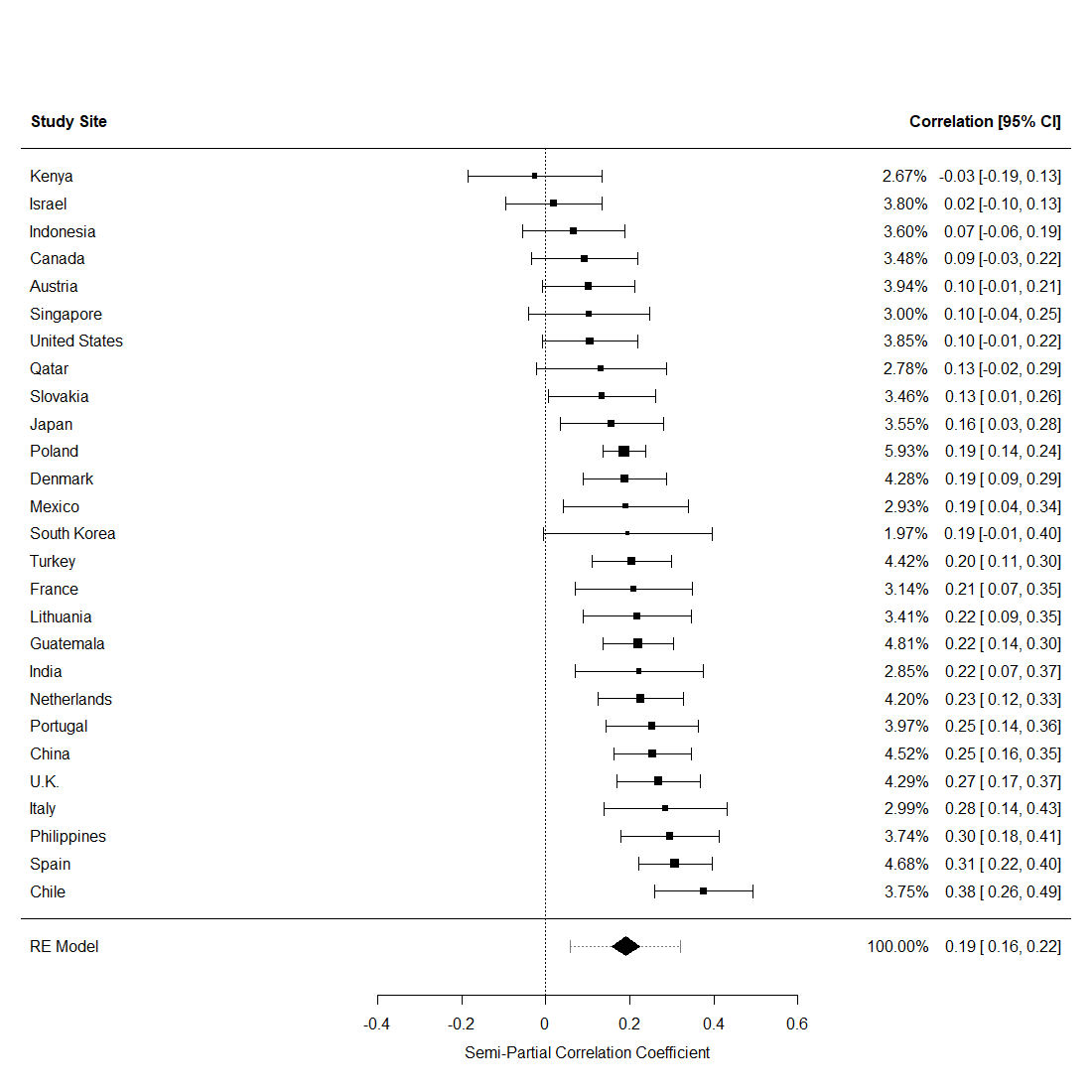


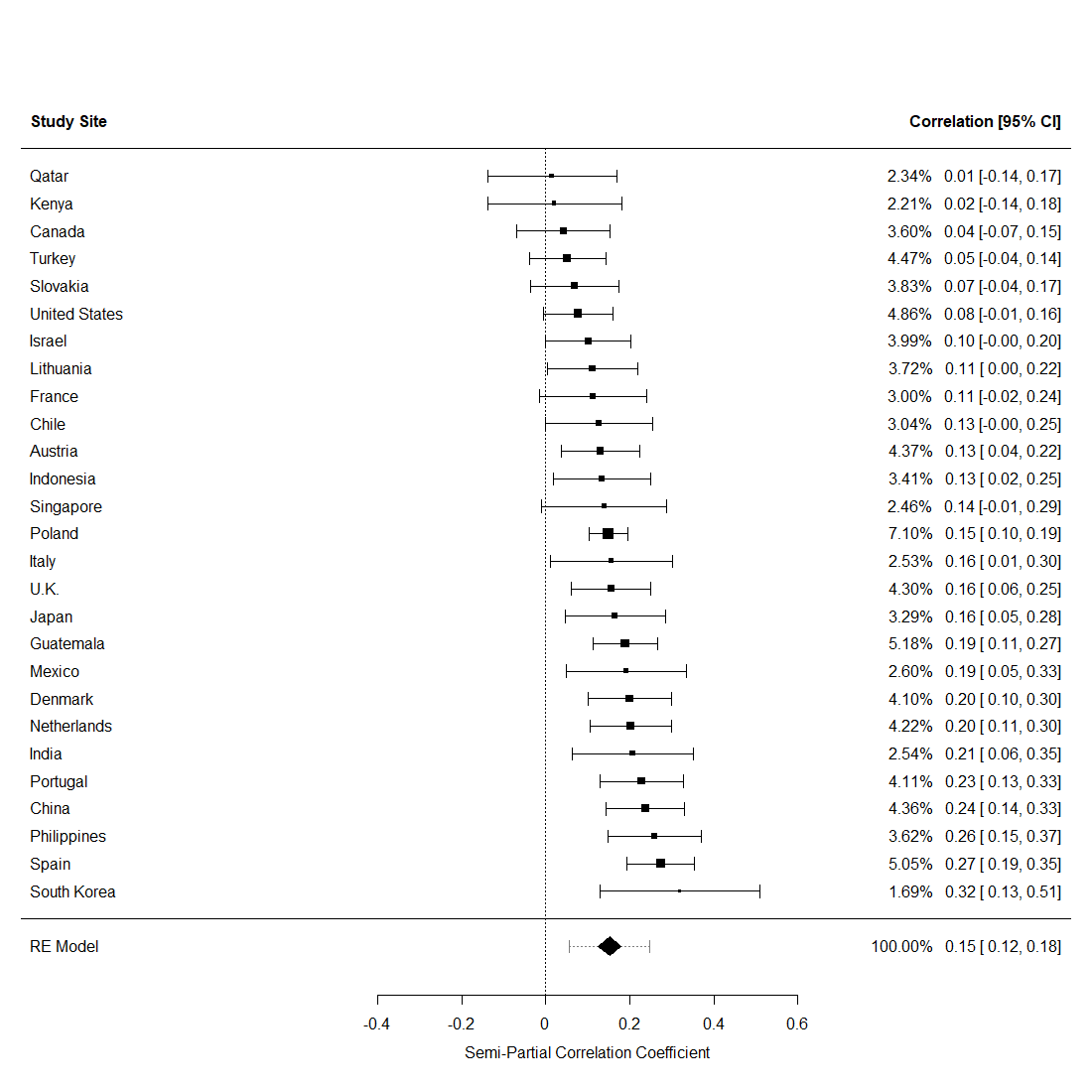


**Figure S13.** Results of random-effects meta-analysis examining the *semi-partial correlation* between traditionalism and the *external-facing precautions composite* after *adjusting for the effects of the five suppressor variables*. Compare to Figure 3 in the main text.

### Traditionalism scale development

We included two pre-validated scales in order to measure traditionalism. First, we included the 6-item conventionalism subscale of the Aggression-Submission-Conventionalism scale^1^, which measures the general tendency to endorse one’s society’s traditional social norms, setting aside the actual content of those traditions (e.g., “Traditions are the foundation of a healthy society and should be respected”). Items were rated on 7-point scales, from “strongly disagree” to “strongly agree”, and half of the items were reverse coded (e.g., “People emphasize tradition too much.”). Second, we used the 4-item authority subscale from the Moral Foundations Questionnaire Short Version^2,32^, which similarly assesses whether individuals respect traditions and authorities, both generally (e.g., “To what extent are the following considerations relevant to your thinking… Whether or not someone conformed to the traditions of society.”), and in relation to specific values regarding gender and age roles (e.g., “Respect for authority is something all children need to learn.”). Items were rated on 7-point scales, either from “Not at all relevant”, to “Extremely relevant”, or from “Strongly Disagree” to “Strongly Agree”. In particular, we chose to include the Moral Foundations and Conventionalism items because they have been widely tested in many languages in cross-cultural psychology research^33–35^.

Because we wanted to measure the tendency to endorse traditional social norms as a general dimension of individual difference as broadly as possible, we were interested in whether all ten traditionalism items together (six from the Conventionalism scale, and four from the Moral Foundations authority scale) would reliably load onto a single traditionalism factor. First, we examined the correlation structure between the ten items by study site. We observed that, at some study sites, the three reverse-coded items from the conventionalism scale did not strongly and reliably negatively correlate with the positively coded items as would be expected (see Table S6 for the correlation between negatively-coded and positively-coded Conventionalism items by study site). Following a literature review, we found that reverse-coded items may frequently be problematic in cross-cultural psychological research^36–38^. We therefore dropped the three reverse-coded conventionalism items before conducting the factor analysis.


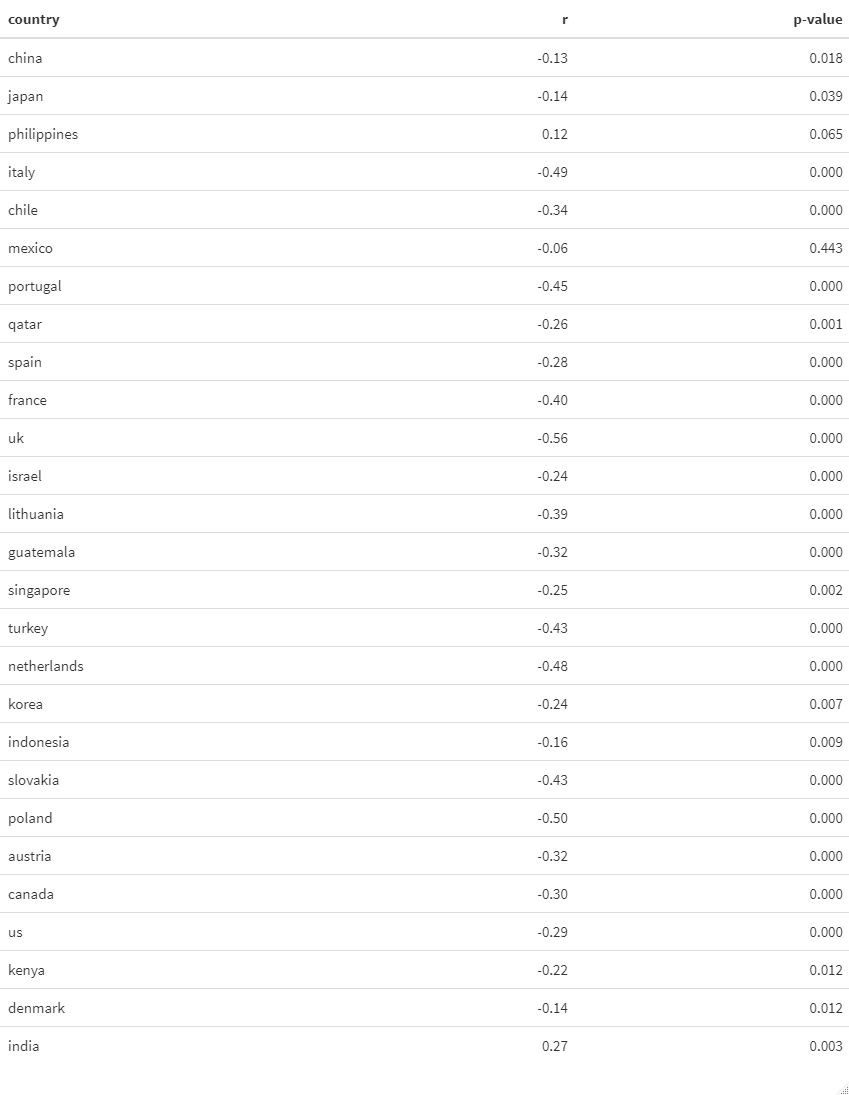


**Table S6.** Product-moment correlations between reverse and non-reverse coded conventionalism items by study site.

We then ran an exploratory factor analysis on the pooled sample across all study sites to determine whether it was appropriate to group the conventionalism and moral foundations authority items (minus the reverse-coded items, see above) into a single factor. First, we used the Kaieser-Meyer-Olkin (KMO) and Bartlett’s test to determine whether these items were suitable for structure detection. The KMO test suggested that the strength of the relationships among the variables was moderately high (KMO = .75), and Bartlett’s test was significant (χ2 (21) = 15,315.74, p < .001), suggesting that the use of factor analysis was appropriate.

The R package *parameters* ^22^ was then used to determine how many factors to extract. There was the most agreement between methods for a one-factor solution (see Figure S14).


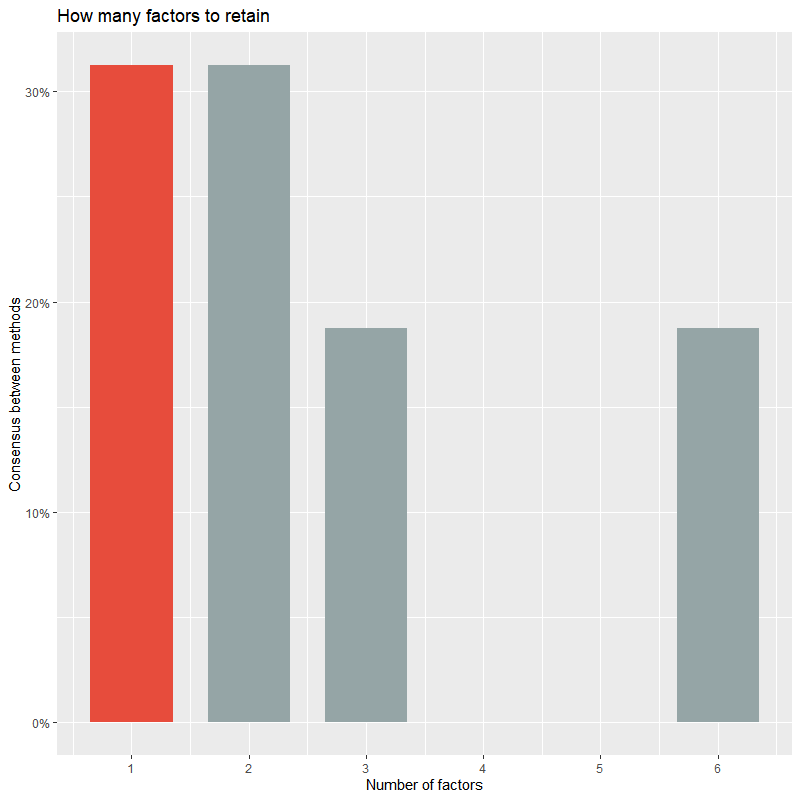


**Figure S14.** Graphical representation showing agreement between different methods for determining the number of factors to retain.

A factor analysis was then conducted with minimum residual extraction, and a Pearson’s correlation matrix. A single factor was extracted. The single factor had a sum of squared loadings of 2.43, and explained 35% of the variance. To create a composite traditionalism factor, we then averaged together those items with factor loadings greater than .5, comprising six out of the seven items (see Table S7). Scale reliability for this composite varied quite widely across study sites (see Table S8).

| **Factor Analysis** |  |
| --- | --- |
|  | **Factor 1** |
| Traditions should be respected – conventionalism scale | .67 |
| People should follow social norms – conventionalism scale | .63 |
| People should respect social norms – conventionalism scale | .69 |
| Importance of whether people show a lack of respect for authority – moral foundations authority scale | .51 |
| Importance of whether people conform to traditions – moral foundations authority scale | .54 |
| Men and women should have different roles in society – moral foundations authority scale | .44 |
| Children should learn respect for authority – moral foundations authority scale | .60 |

**Table S7.** Factor loadings from an exploratory factor analysis on traditionalism items.


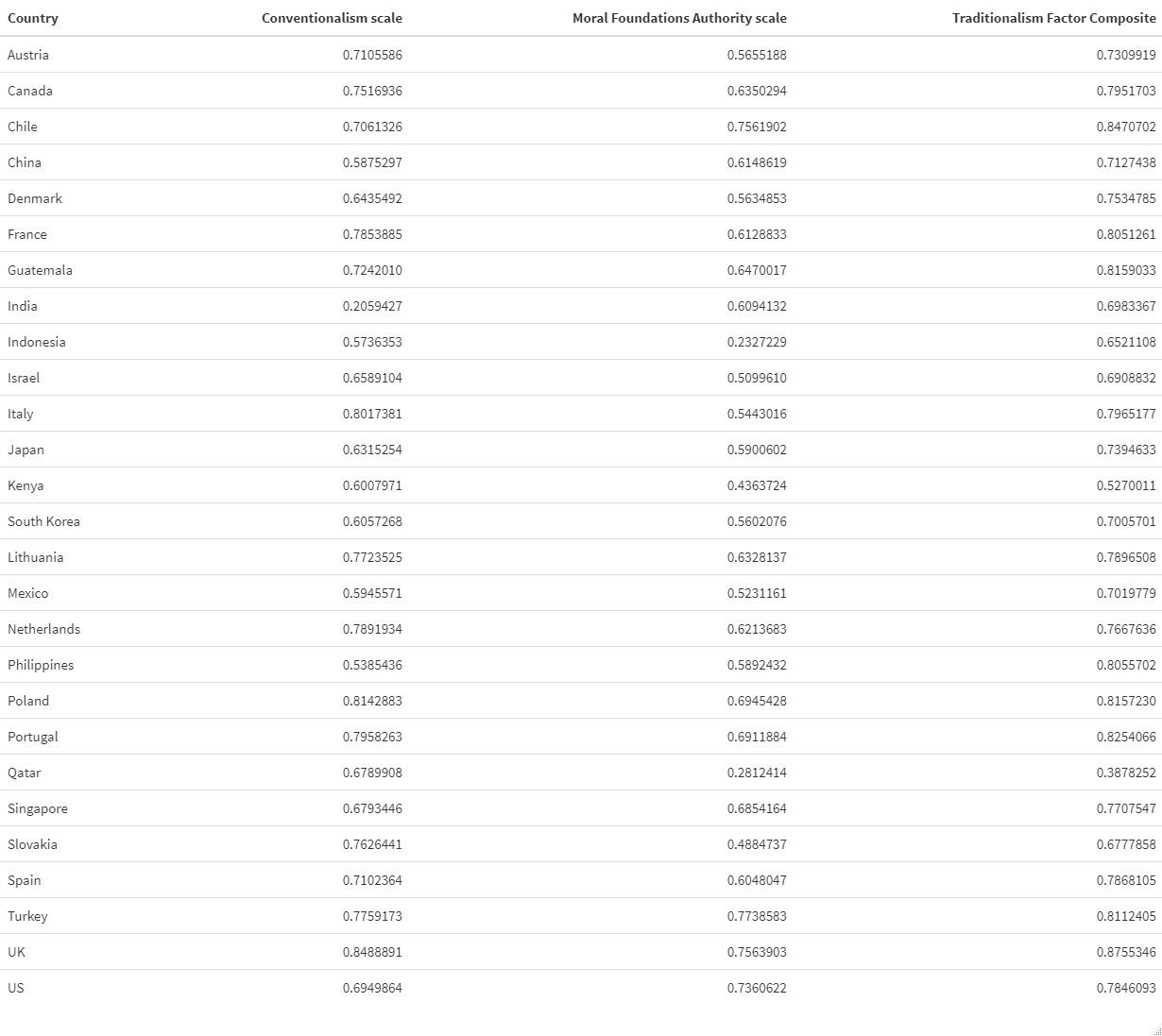


**Table S8.** Cronbach’s alphas by study site for the Traditionalism composite used to measure traditionalism in the main text analyses, as well as for the Moral Foundations Authority and Conventionalism subscales included in the survey.

In order to determine whether the lack of scale reliability at some of the study sites was affecting results, we first conducted meta-analyses that replicated the main text analyses (see Figures 1 and 3), disattenuating for unreliability; this did not conceptually change the results (see Figures S7 and S8). Next, we re-ran the main text meta-analyses using each of the six items individually from our traditionalism factor, in place of the composite traditionalism score (see Figures S15-S26). Although effect sizes varied some, results did not conceptually differ across items, suggesting that the pattern of association between traditionalism-related items and precautions is consistent, and low scale reliability at some study sites did not conceptually impact the results.


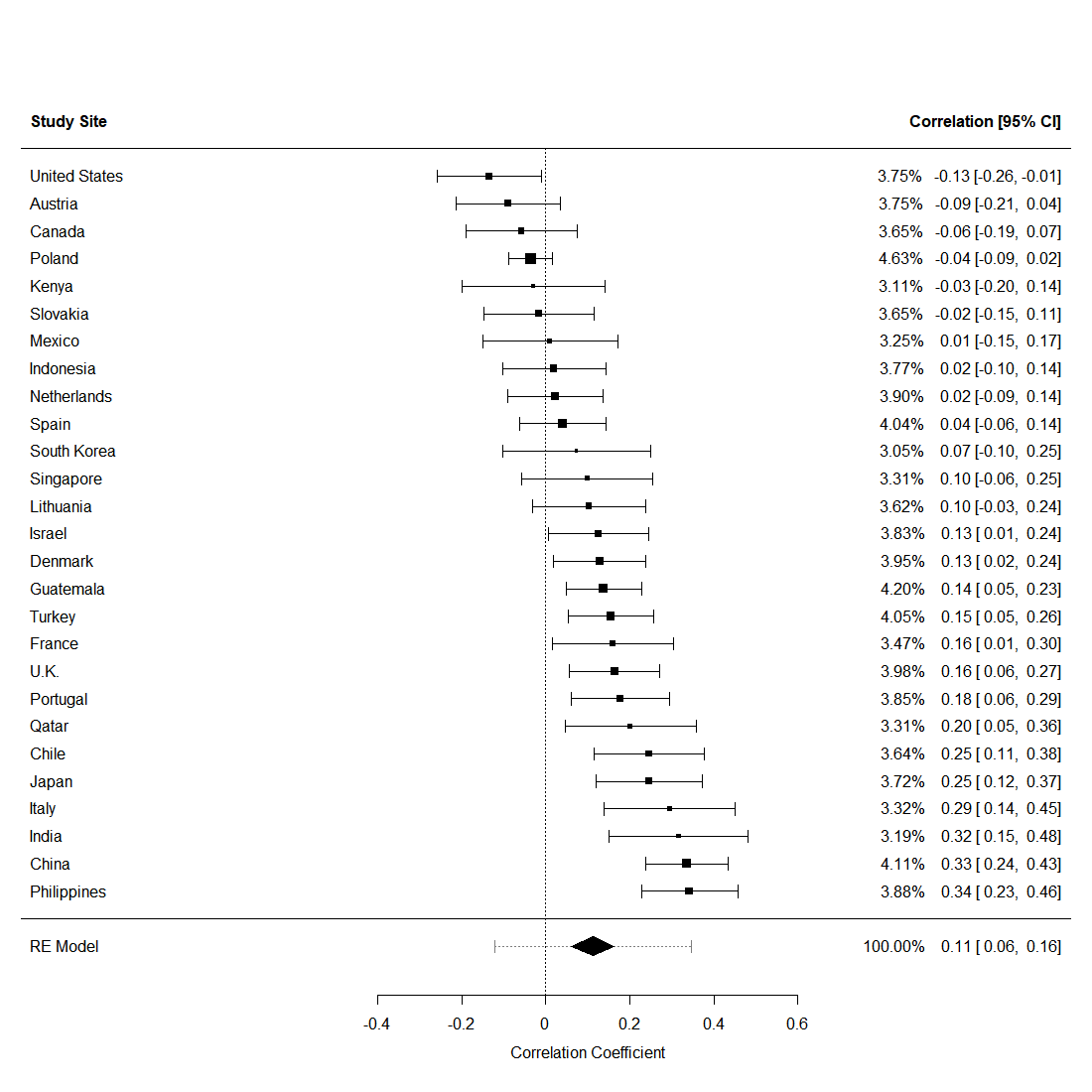


**Figure S15.** Results of random-effects meta-analysis examining the *zero-order correlation* between the traditionalism item *traditions should be respected* and the COVID-19 health precautions composite. Compare to Figure 1 in the main text.


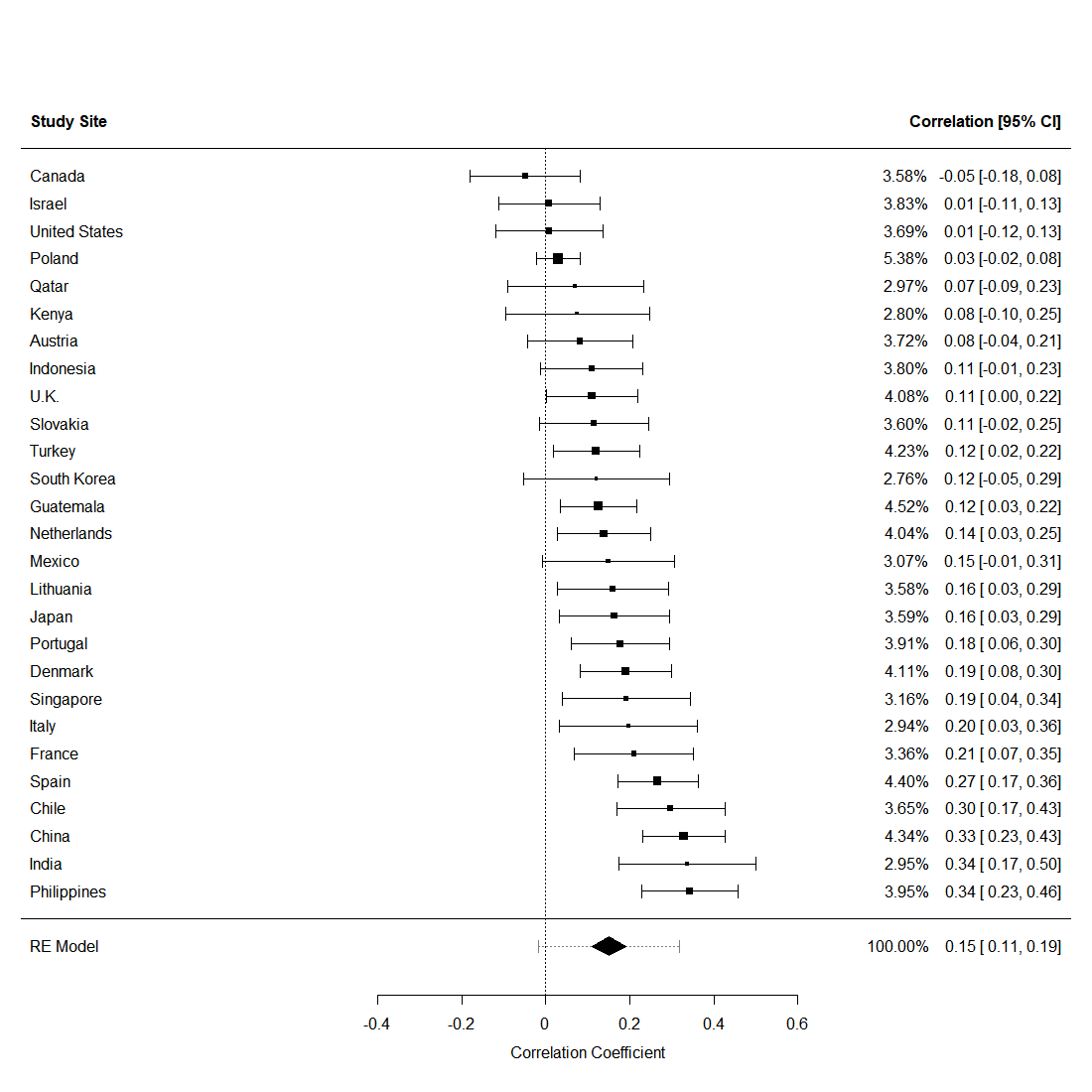


**Figure S16.** Results of random-effects meta-analysis examining the *zero-order correlation* between the traditionalism item *people should follow social norms* and the COVID-19 health precautions composite. Compare to Figure 1 in the main text.


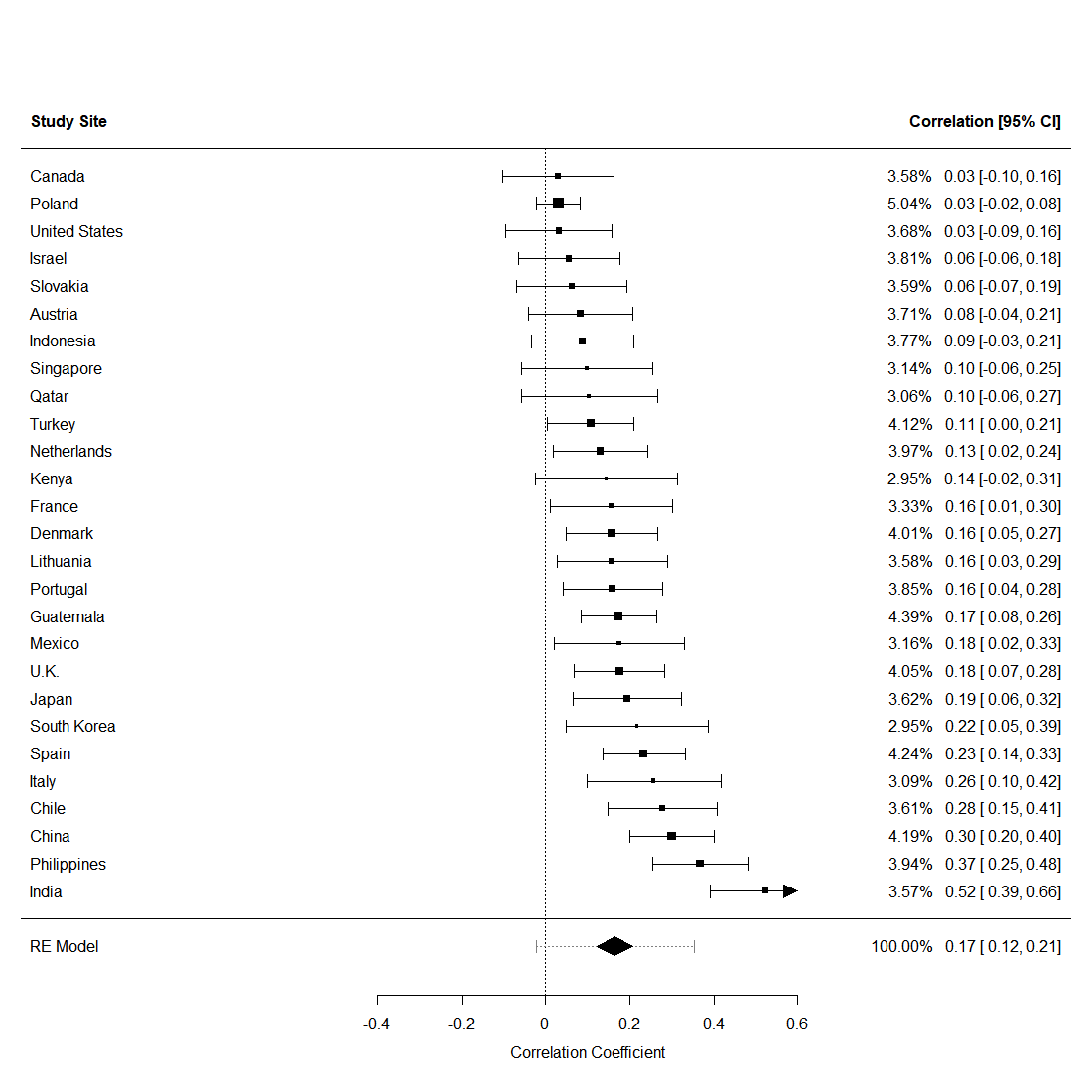


**Figure S17.** Results of random-effects meta-analysis examining the *zero-order correlation* between the traditionalism item *people should respect social norms* and the COVID-19 health precautions composite. Compare to Figure 1 in the main text.


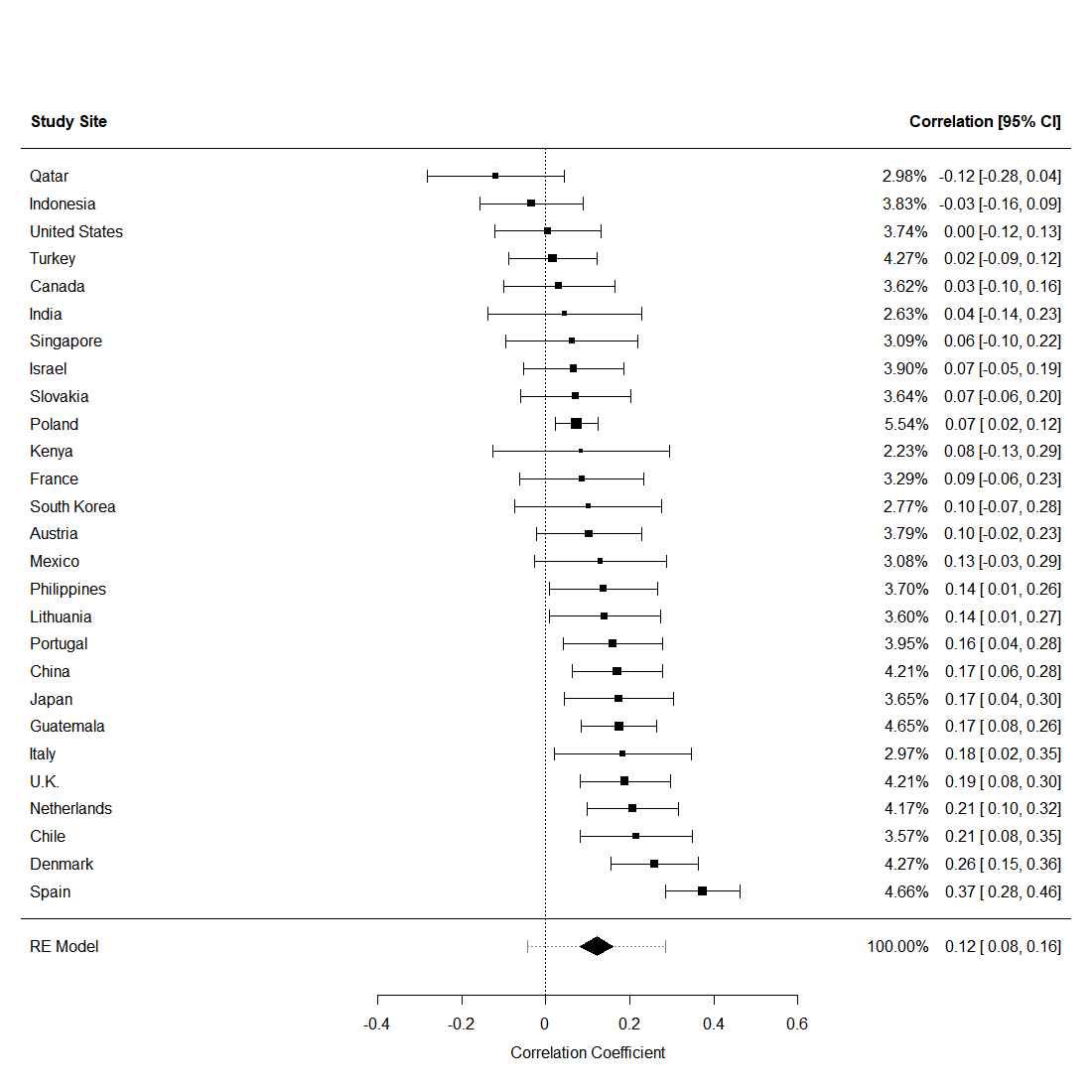


**Figure S18.** Results of random-effects meta-analysis examining the *zero-order correlation* between the traditionalism item *importance of whether people show a lack of respect for authority* and the COVID-19 health precautions composite. Compare to Figure 1 in the main text.


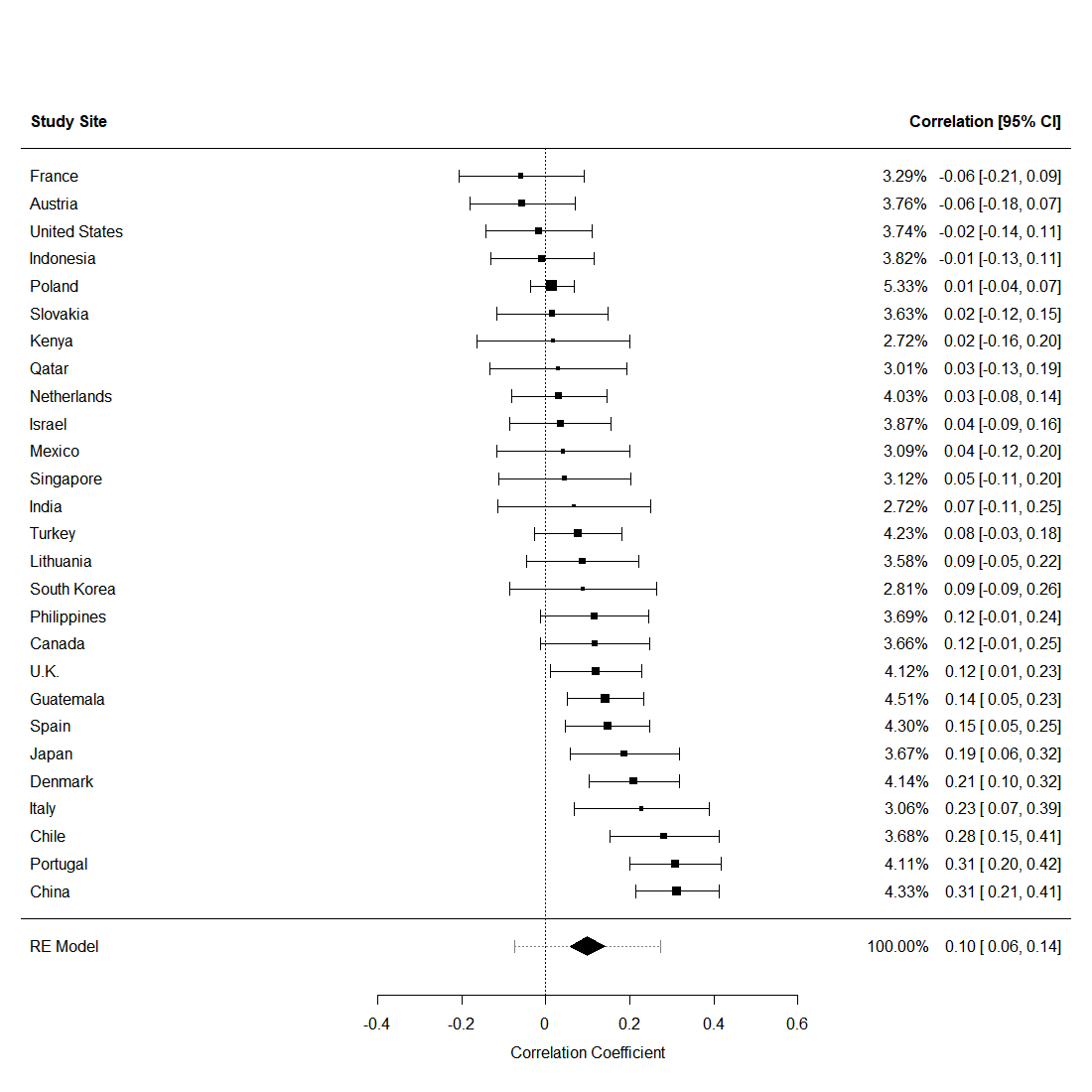


**Figure S19.** Results of random-effects meta-analysis examining the *zero-order correlation* between the traditionalism item *importance of whether people conform to traditions* and the COVID-19 health precautions composite. Compare to Figure 1 in the main text.


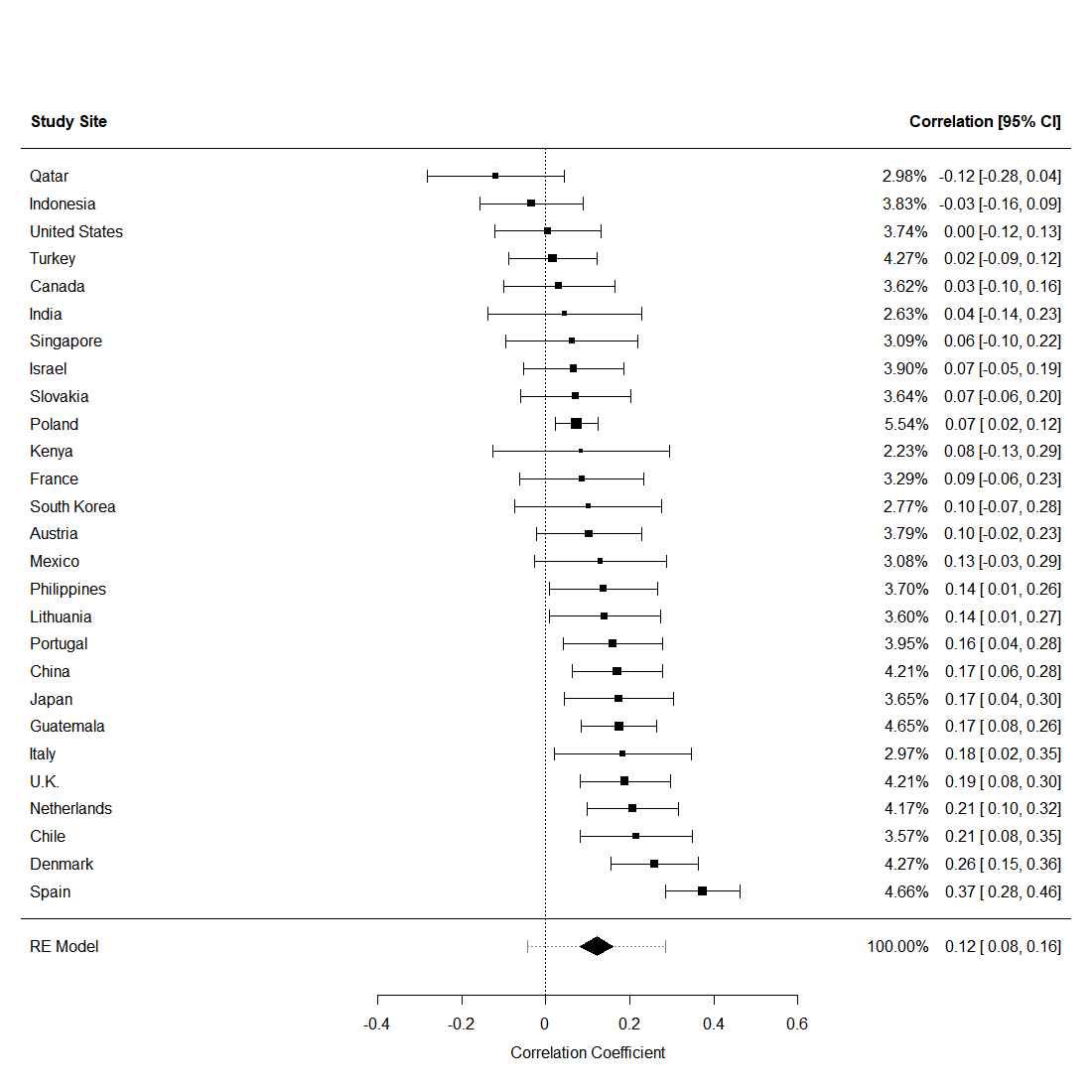


**Figure S20.** Results of random-effects meta-analysis examining the *zero-order correlation* between the traditionalism item *children should learn respect for authority* and the COVID-19 health precautions composite. Compare to Figure 1 in the main text.


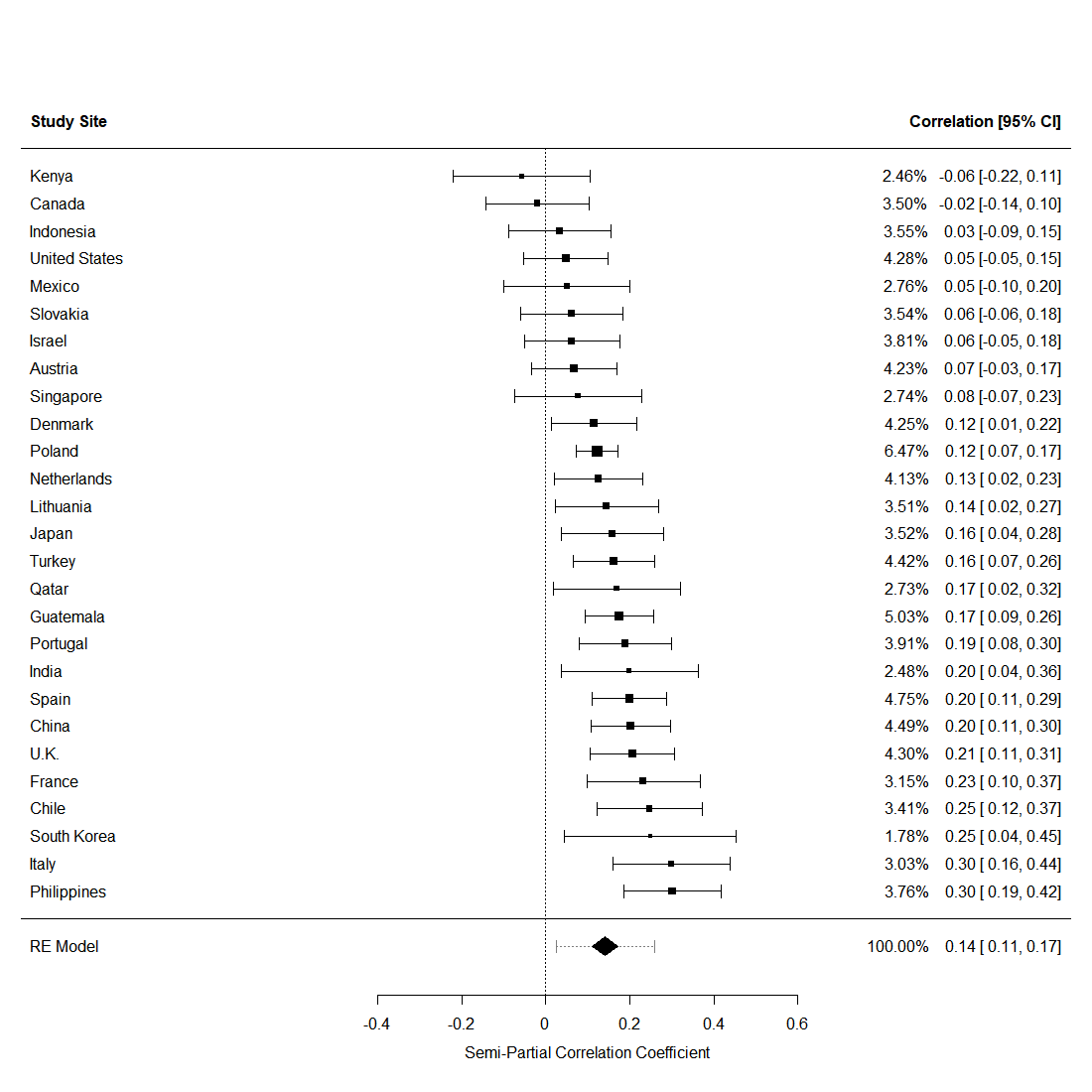


**Figure S21.** Results of random-effects meta-analysis examining the *semi-partial correlation* between the traditionalism item *traditions should be respected* and the COVID-19 health precautions composite after *adjusting for the effects of the five suppressor variables*. Compare to Figure 3 in the main text.


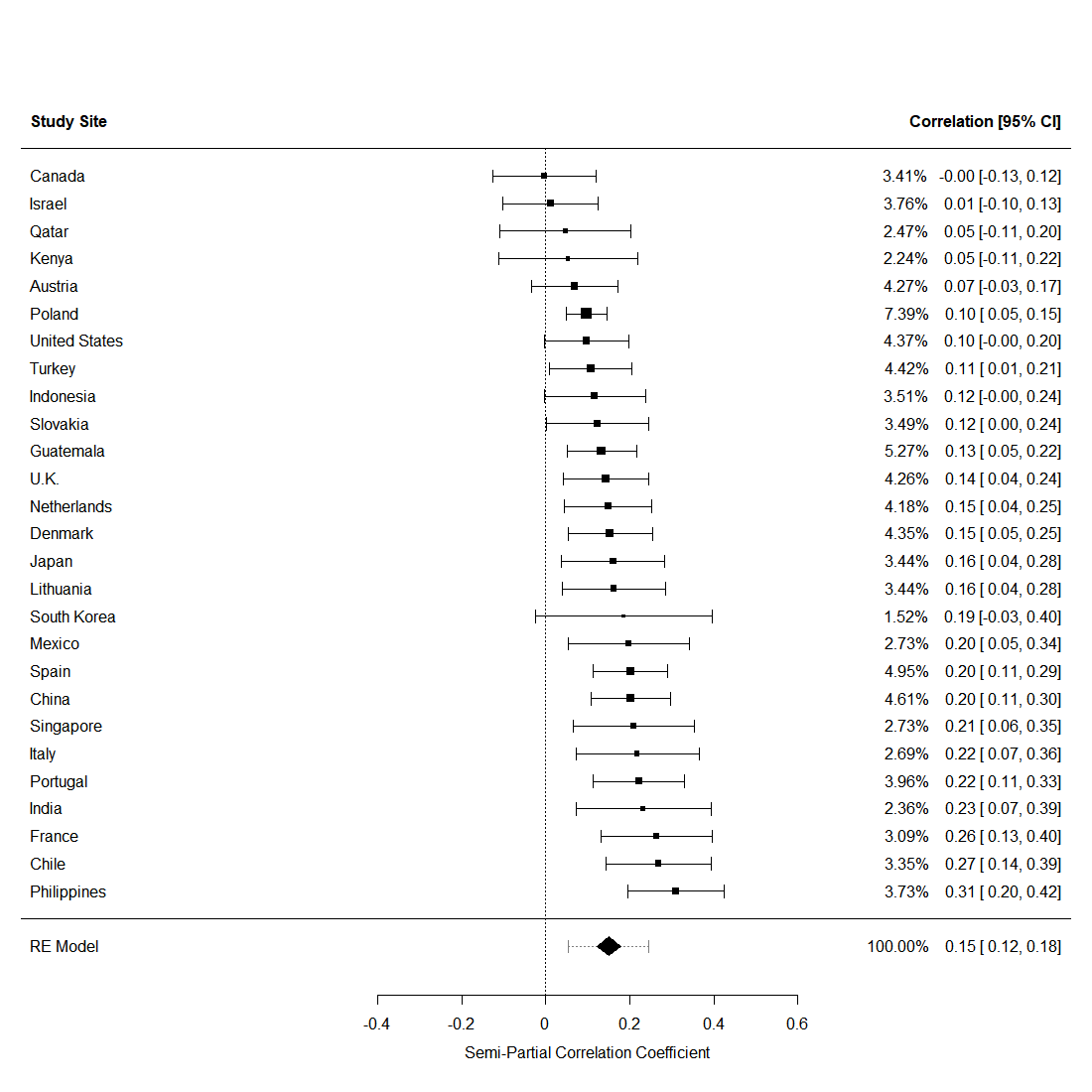


**Figure S22.** Results of random-effects meta-analysis examining the *semi-partial correlation* between the traditionalism item *people should follow social norms* and the COVID-19 health precautions composite after *adjusting for the effects of the five suppressor variables*. Compare to Figure 3 in the main text.


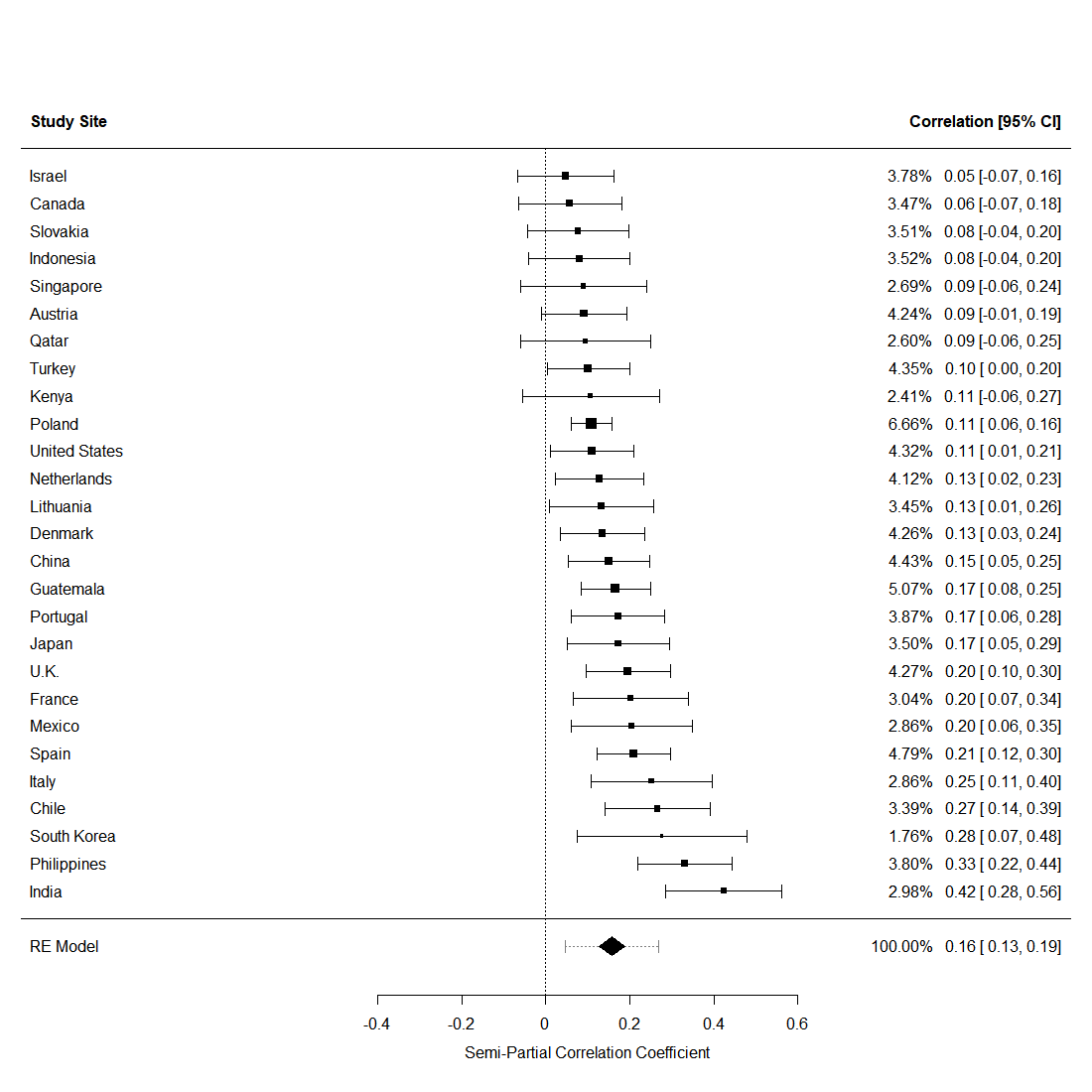


**Figure S23.** Results of random-effects meta-analysis examining the *semi-partial correlation* between the traditionalism item *people should respect social norms* and the COVID-19 health precautions composite after *adjusting for the effects of the five suppressor variables*. Compare to Figure 3 in the main text.


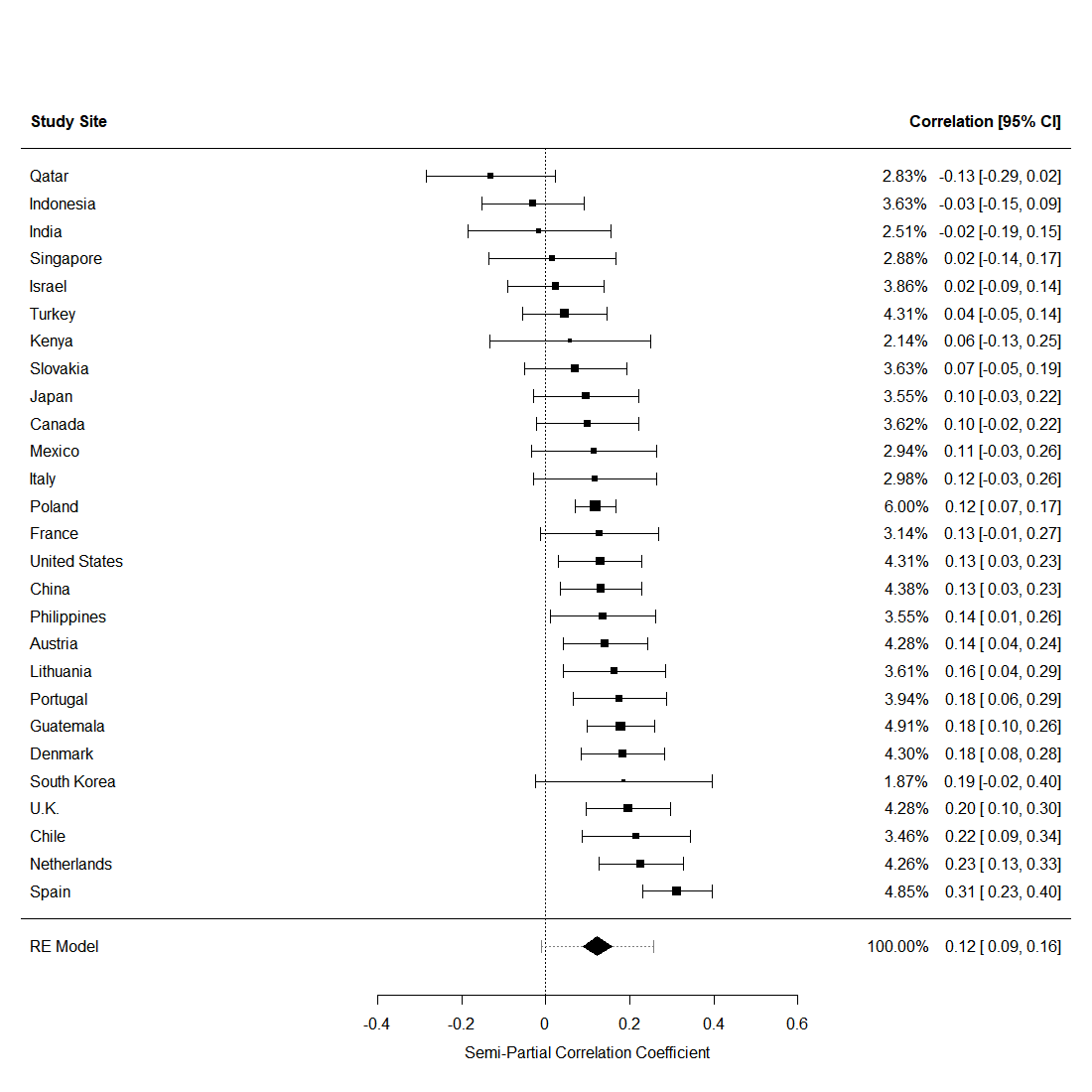


**Figure S24.** Results of random-effects meta-analysis examining the *semi-partial correlation* between the traditionalism item *importance of whether people show a lack of respect for authority* and the COVID-19 health precautions composite after *adjusting for the effects of the five suppressor variables*. Compare to Figure 3 in the main text.


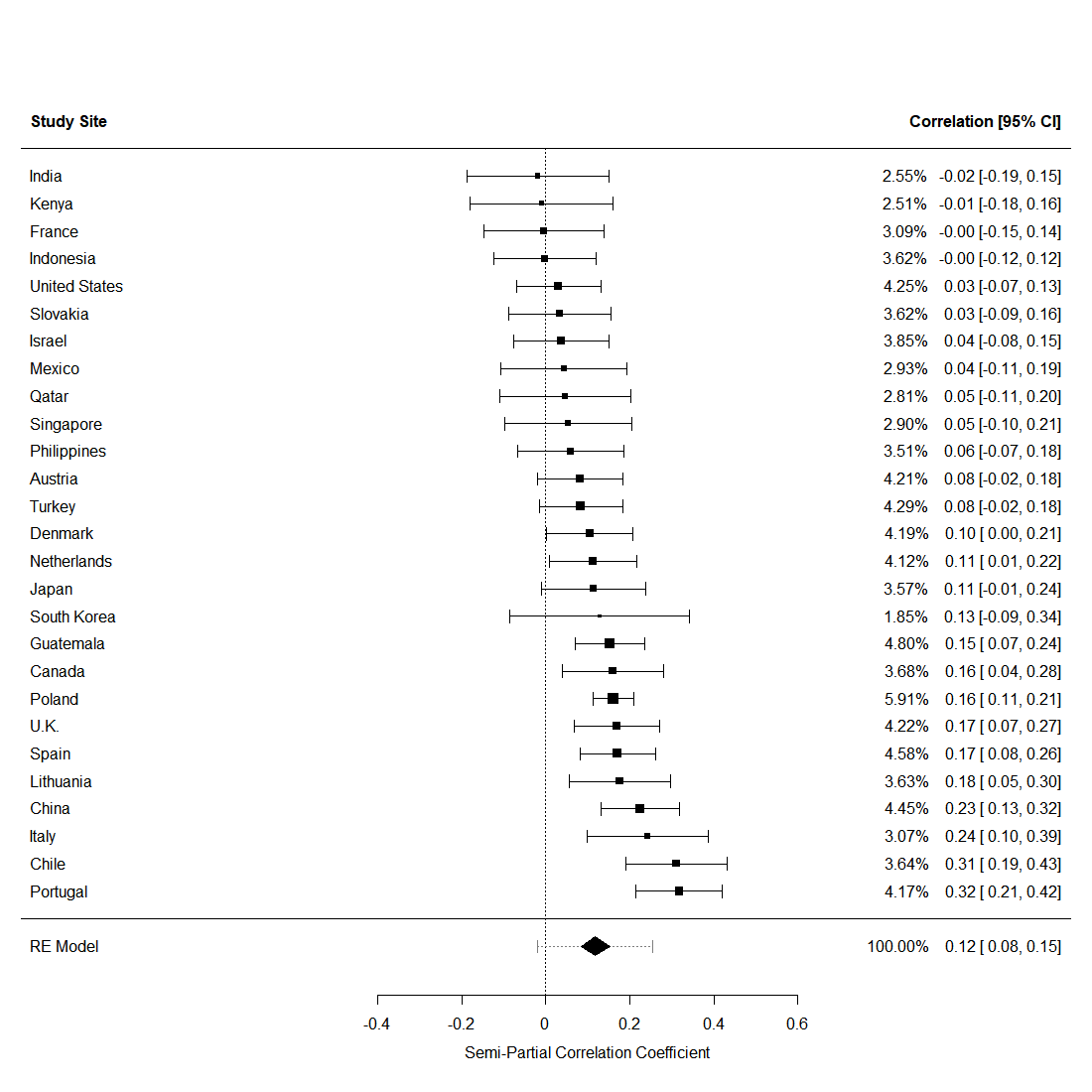


**Figure S25.** Results of random-effects meta-analysis examining the *semi-partial correlation* between the traditionalism item *importance of whether people conform to traditions* and the COVID-19 health precautions composite after *adjusting for the effects of the five suppressor variables*. Compare to Figure 3 in the main text.


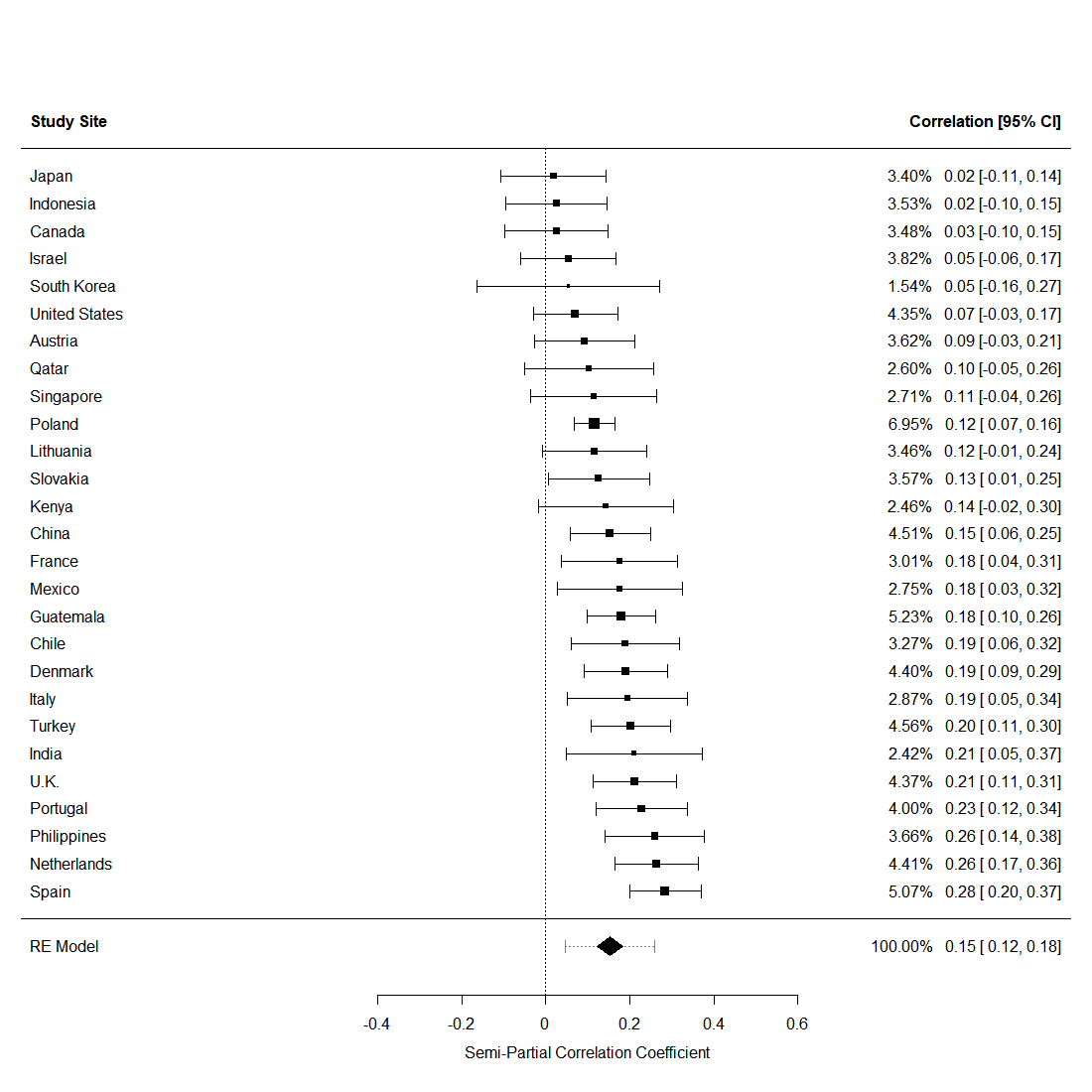


**Figure S26.** Results of random-effects meta-analysis examining the *semi-partial correlation* between the traditionalism item *children should learn respect for authority* and the COVID-19 health precautions composite after *adjusting for the effects of the five suppressor variables*. Compare to Figure 3 in the main text.

We also tested whether results reported with the traditionalism composite were sensitive to the fact that the reverse-coded conventionalism items had been removed. main text results did not substantially conceptually change when we used a more expansive traditionalism composite that included all items (including the reverse coded ones) from the conventionalism and moral foundations authority subscales (see Figures S27-S28). However, effect sizes were smaller, which we attribute to the noise introduced by the reverse-coded items. Likewise, meta-analysis results did not conceptually change when substituting composites based on either the original Conventionalism or Moral Foundations authority subscales for the main text traditionalism composite (see Figures S29-S32), although effect sizes tended to be lower due to increased noise. See Table S8 for scale reliabilities for the Conventionalism and Authority composites. Taken in sum, traditionalism could be measured in several different ways based on the data that were collected. Although there were researcher degrees of freedom in making decisions about how to construct a reliable traditionalism composite, using alternative decision-points results in conceptually similar findings, suggesting that results are robust to these kinds of research decisions.


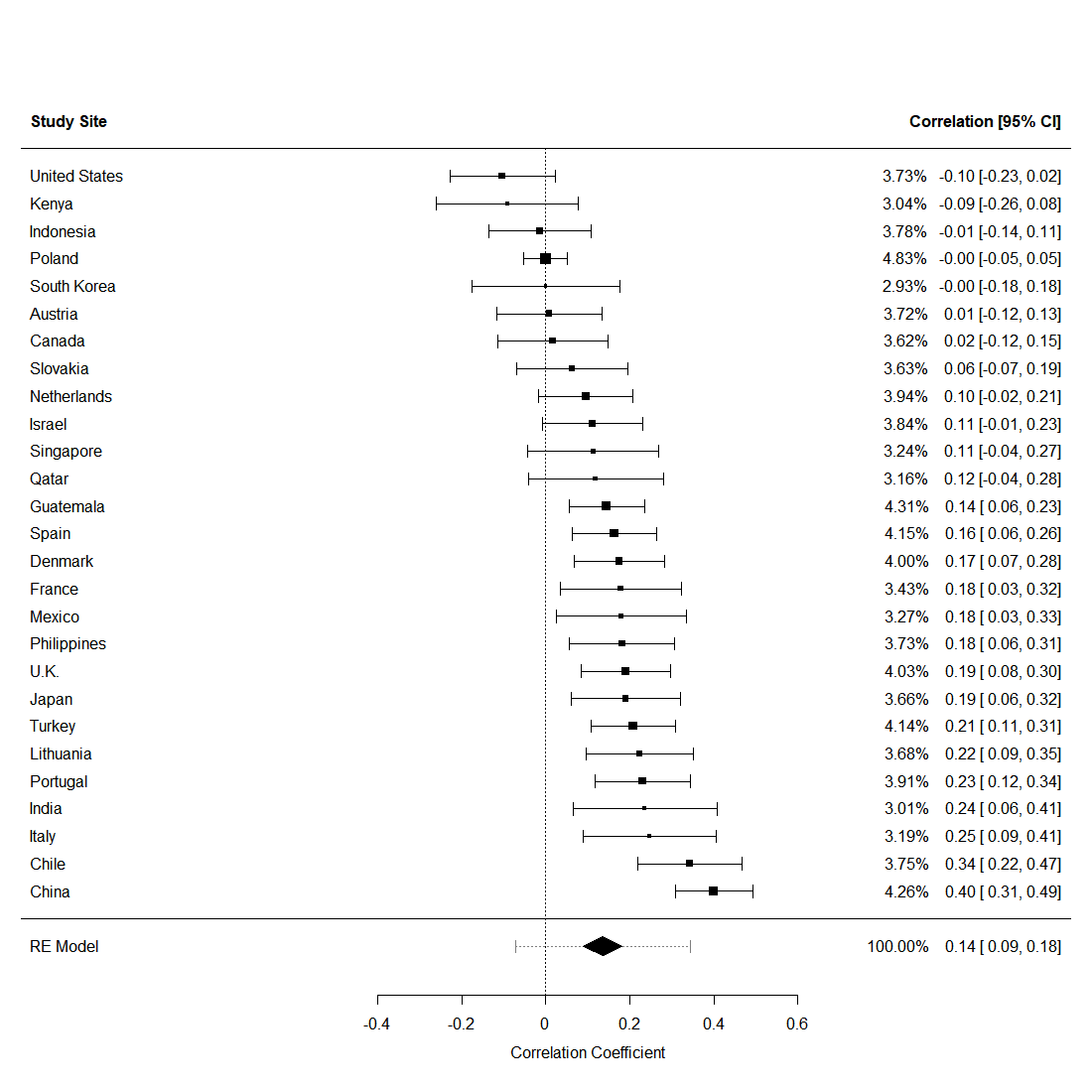


**Figure S27.** Results of random-effects meta-analysis examining the *zero-order correlation* between a traditionalism composite comprising all items from the Conventionalism and Moral Foundations Authority subscales (including reverse-coded ones) and the COVID-19 health precautions composite. Compare to Figure 1 in the main text.


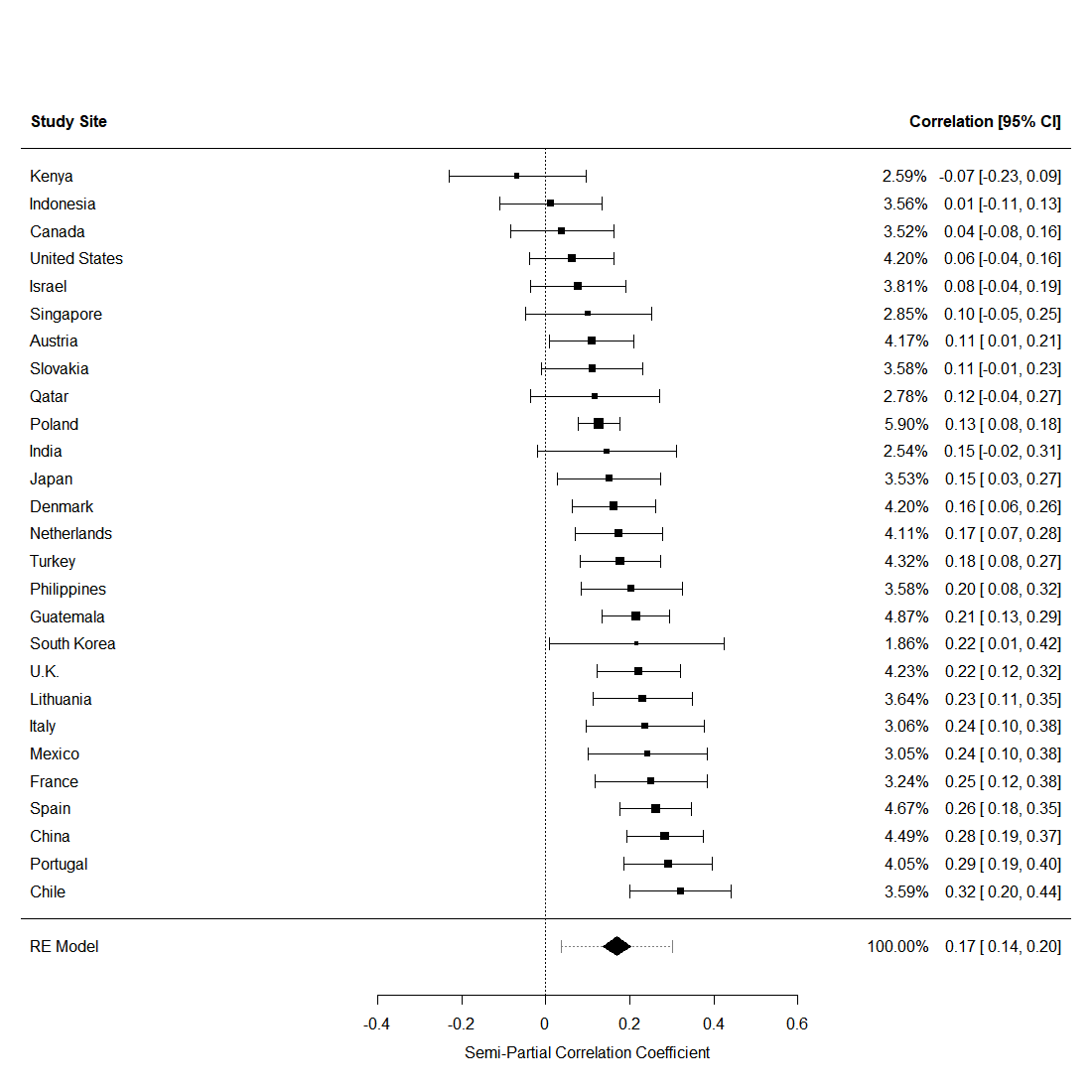


**Figure S28.** Results of random-effects meta-analysis examining the *semi-partial correlation* between a traditionalism composite comprising all items from the Conventionalism and Moral Foundations Authority subscales (including reverse-coded ones) and the COVID-19 health precautions composite after adjusting for the effects of the five suppressor variables. Compare to Figure 3 in the main text.


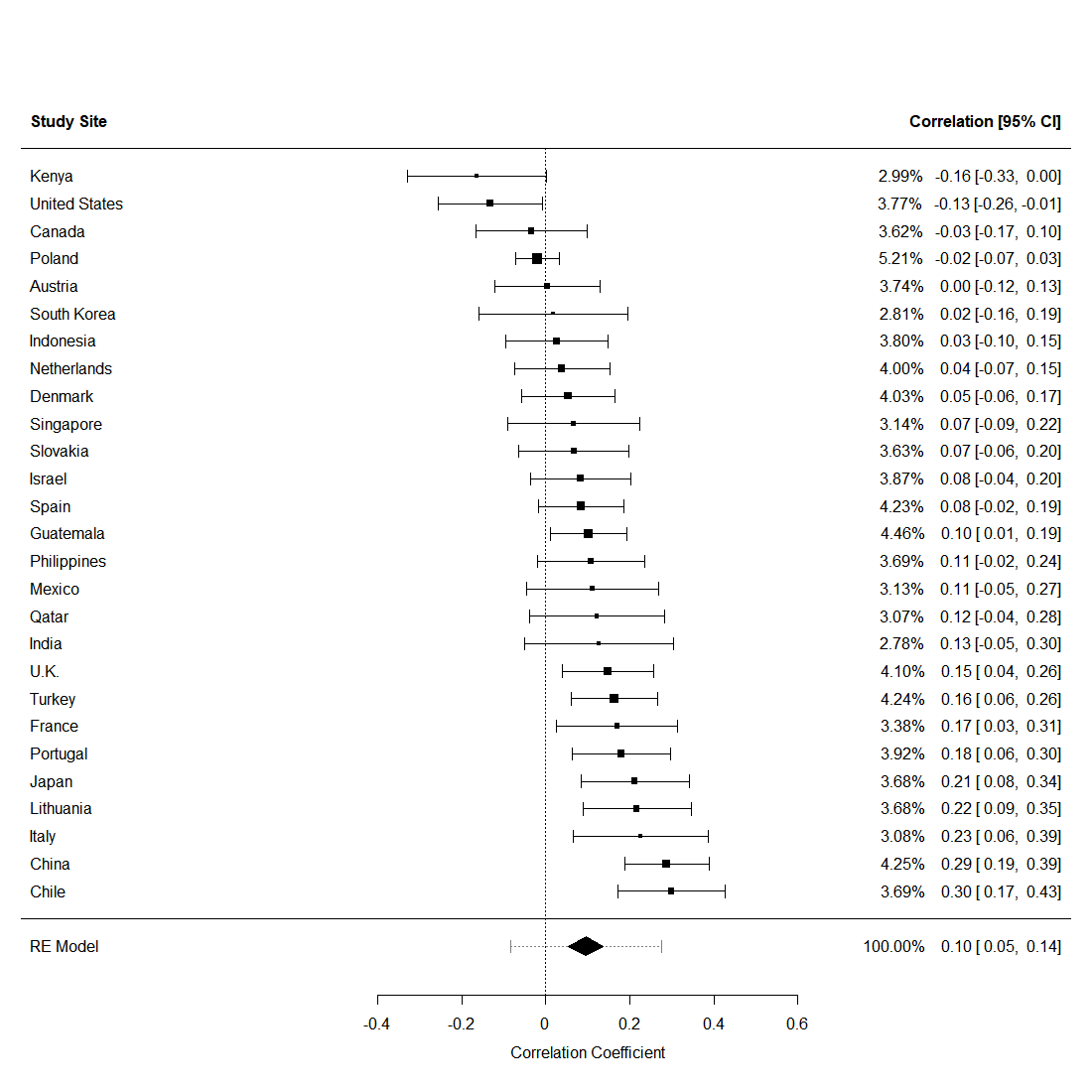


**Figure S29.** Results of random-effects meta-analysis examining the *zero-order correlation* between the Conventionalism subscale and the COVID-19 health precautions composite. Compare to Figure 1 in the main text.

**Figure S30.** Results of random-effects meta-analysis examining the *semi-partial correlation* between the Conventionalism subscale and the COVID-19 health precautions composite after adjusting for the effects of the five suppressor variables. Compare to Figure 3 in the main text.


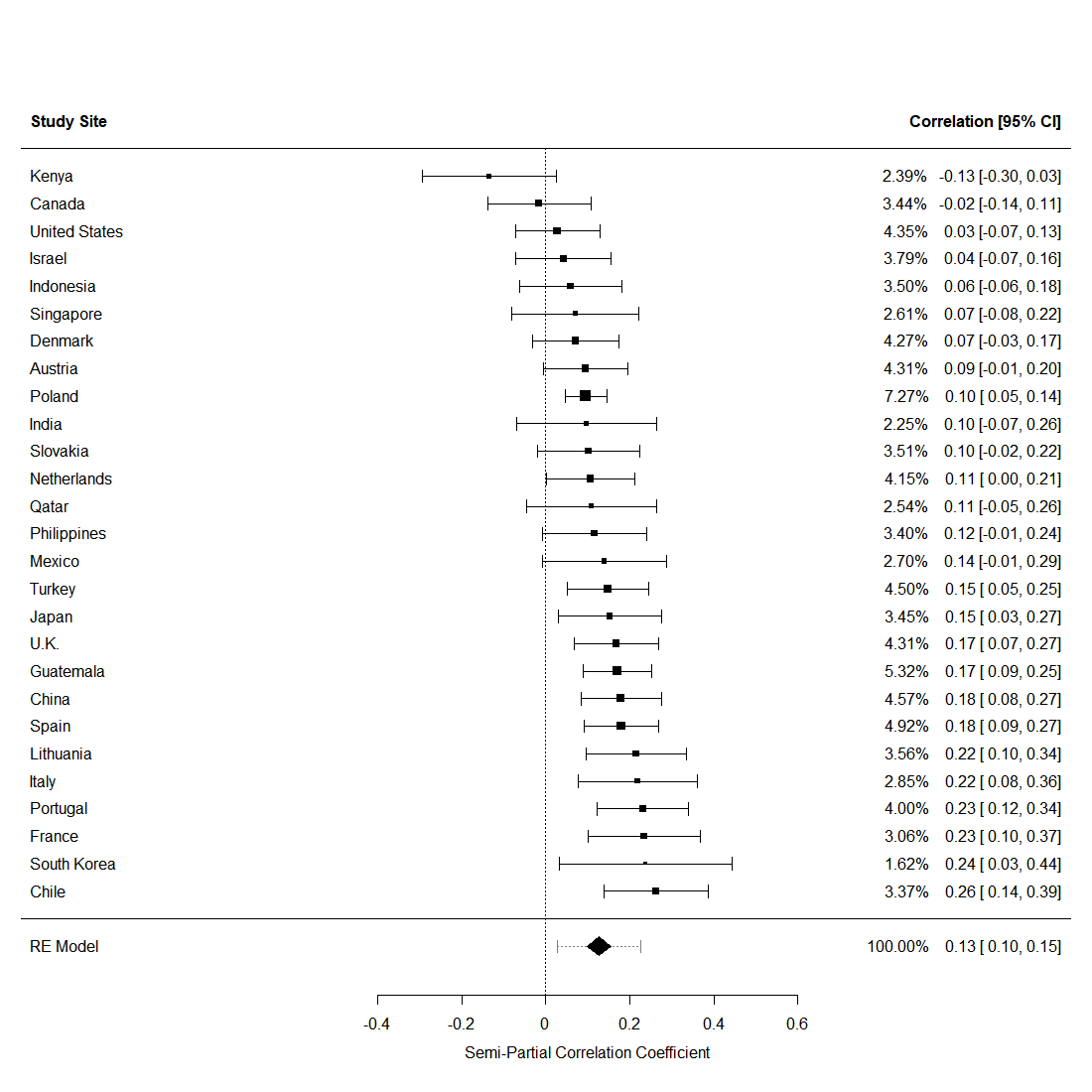


**Figure S31.** Results of random-effects meta-analysis examining the *zero-order correlation* between the Moral Foundations Authority subscale and the COVID-19 health precautions composite. Compare to Figure 1 in the main text.


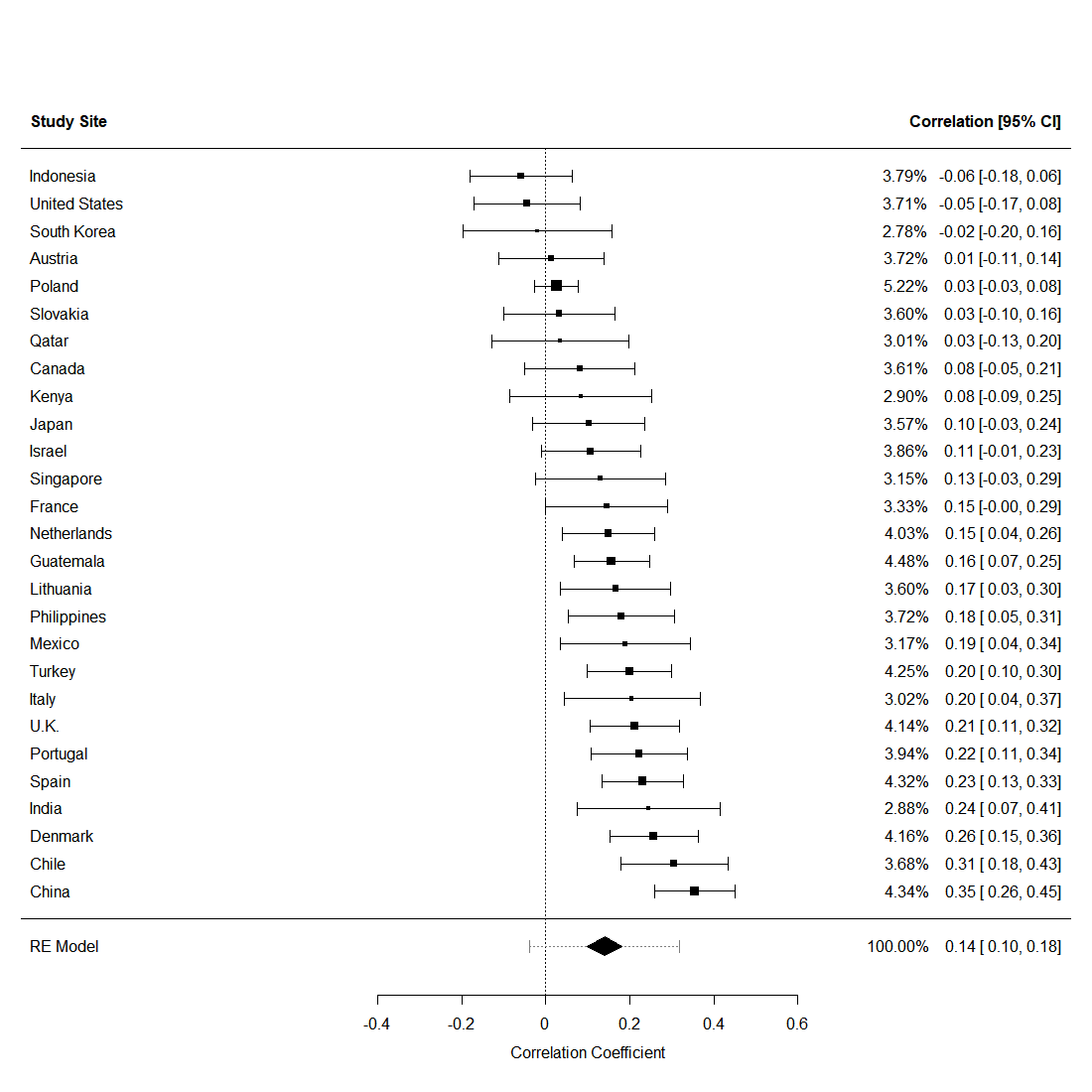


**Figure S32.** Results of random-effects meta-analysis examining the *semi-partial correlation* between the Moral Foundations Authority subscale and the COVID-19 health precautions composite after adjusting for the effects of the five suppressor variables. Compare to Figure 3 in the main text.


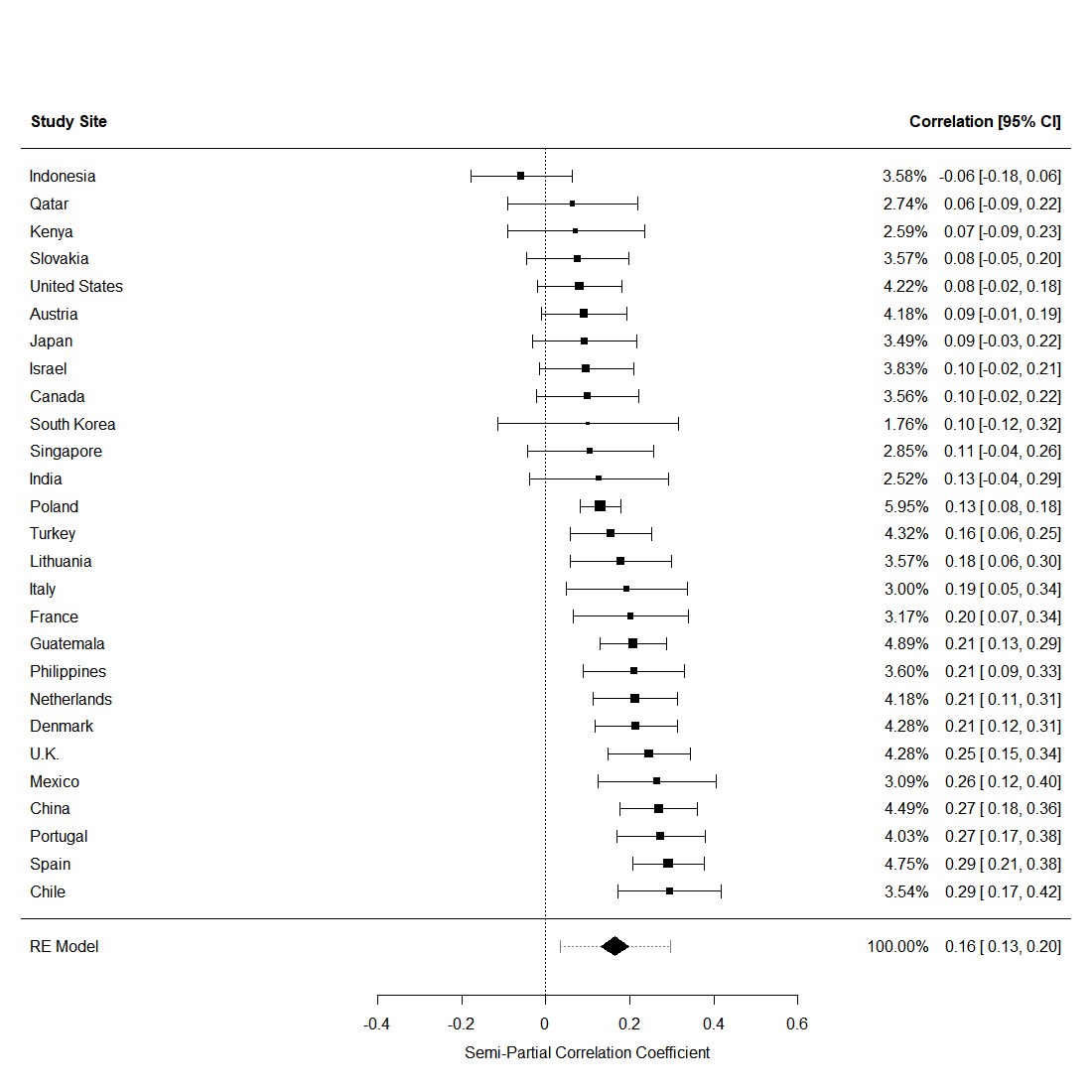


### Traditionalism-precautions relationship using factor scores

In the main text analyses, we used composite variables for traditionalism and COVID-19 precautions that were comprised of raw averages of all the items that loaded onto each respective factor (e.g. traditionalism, internal-facing precautions, etc.). See pages S30 and S38 for details on factor analyses. However, in the main text, we report that using factor scores instead of raw averages in the main text analyses did not conceptually affect the results. Here, we present those results. First, we extracted factor scores for the traditionalism factor, the internal-facing precautions factor, the external-facing precautions factor, and the combined overall public health precautions factor (where all public health precautions items—both external- and internal-facing—load onto a single factor). Then, we examined the correlations between the factor scores, and the composite variables based on raw averages. We fit a series of random-intercepts linear mixed models (estimated using REML) to examine the relationships between the factor scores and composited averages, including country as a random effect. For all four variables, the factor scores were highly correlated with the composited averages, as follows: traditionalism (marginal R2 = .97, β = .98, 95% CI [.97, .98), t(7,304) = 491.55, *p* < .001); internal-facing precautions (marginal R2 = .96, β = .98, 95% CI [.98, .99), t(7,462) = 396.65, *p* < .001); external-facing precautions (marginal R2 = .97, β = .98, 95% CI [.98, .98), t(7,462) = 500.29, *p* < .001); overall public health precautions (marginal R2 = .98, β = .99, 95% CI [.98, .99), t(7,523) = 607.76, *p* < .001).

We then re-analyzed the main text results using the factor scores instead of the composited averages. The relationship between traditionalism and COVID-19 health precautions did not conceptually change as a product of using the factor scores, see Figures S33 and S34. Likewise, the interaction between subscale and traditionalism was conceptually unaltered when using factor scores instead of composited averages (compare to Figure 4 in the main text). Using the factor scores, there was an interaction between health precautions subscale and traditionalism (*B* = .09, SE = .01, *t*(6,993) = 10.37, *p* < .001). A simple slopes analysis revealed that the correlation between traditionalism and internal-facing precautions (*B* = .22, SE = .01, *t*(6,993) = 20.79, *p* < .001) was about twice as strong as the correlation between traditionalism and external-facing precautions (*B* = .13, SE = .01, *t*(6,993) = 12/02, *p* < .001). Note that the factor scores were rescaled to the original 1-7 scale used by participants.


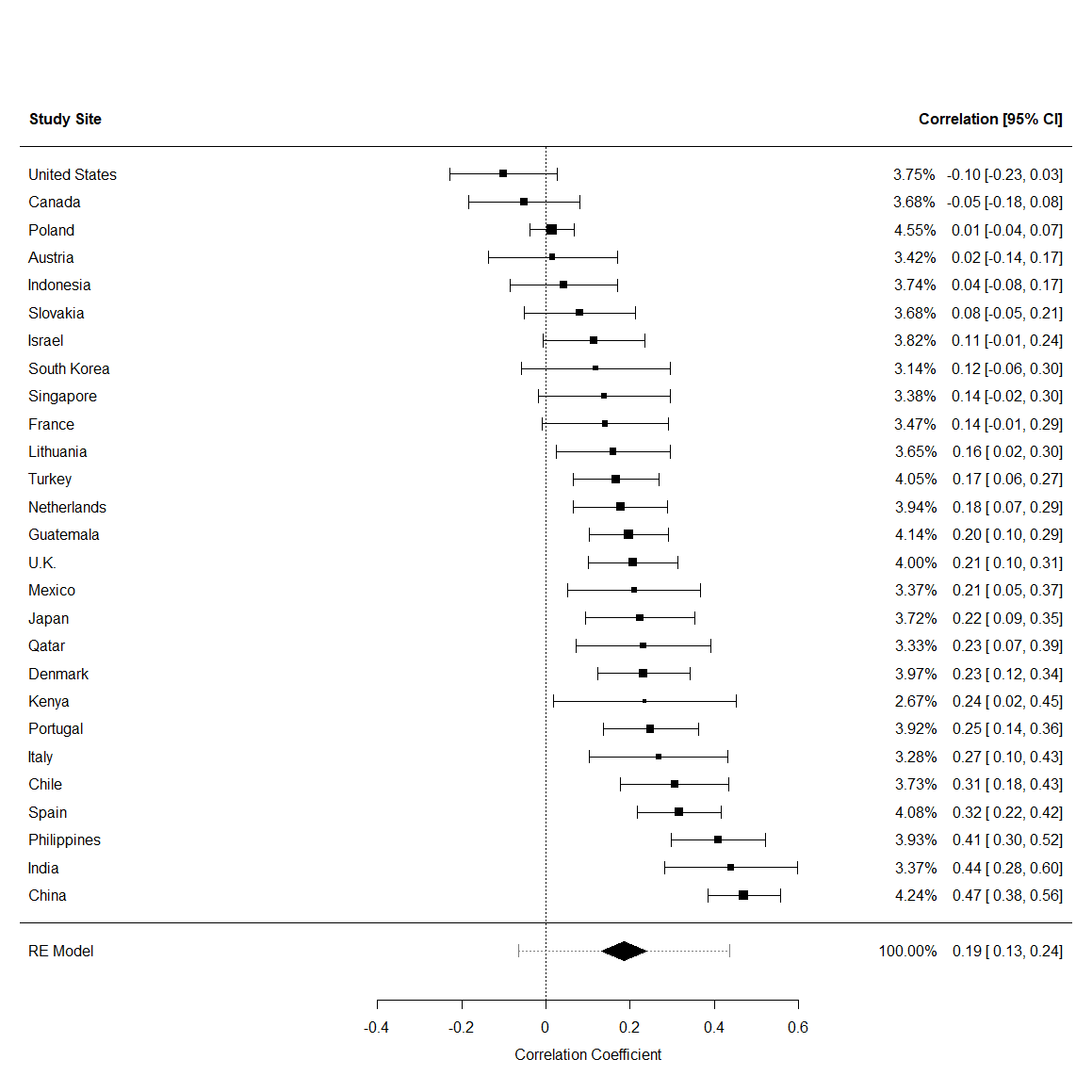


**Figure S33.** Results of random-effects meta-analysis examining the *zero-order correlation* between the traditionalism factor scores and the COVID-19 health precautions factor scores. Compare to Figure 1 in the main text.


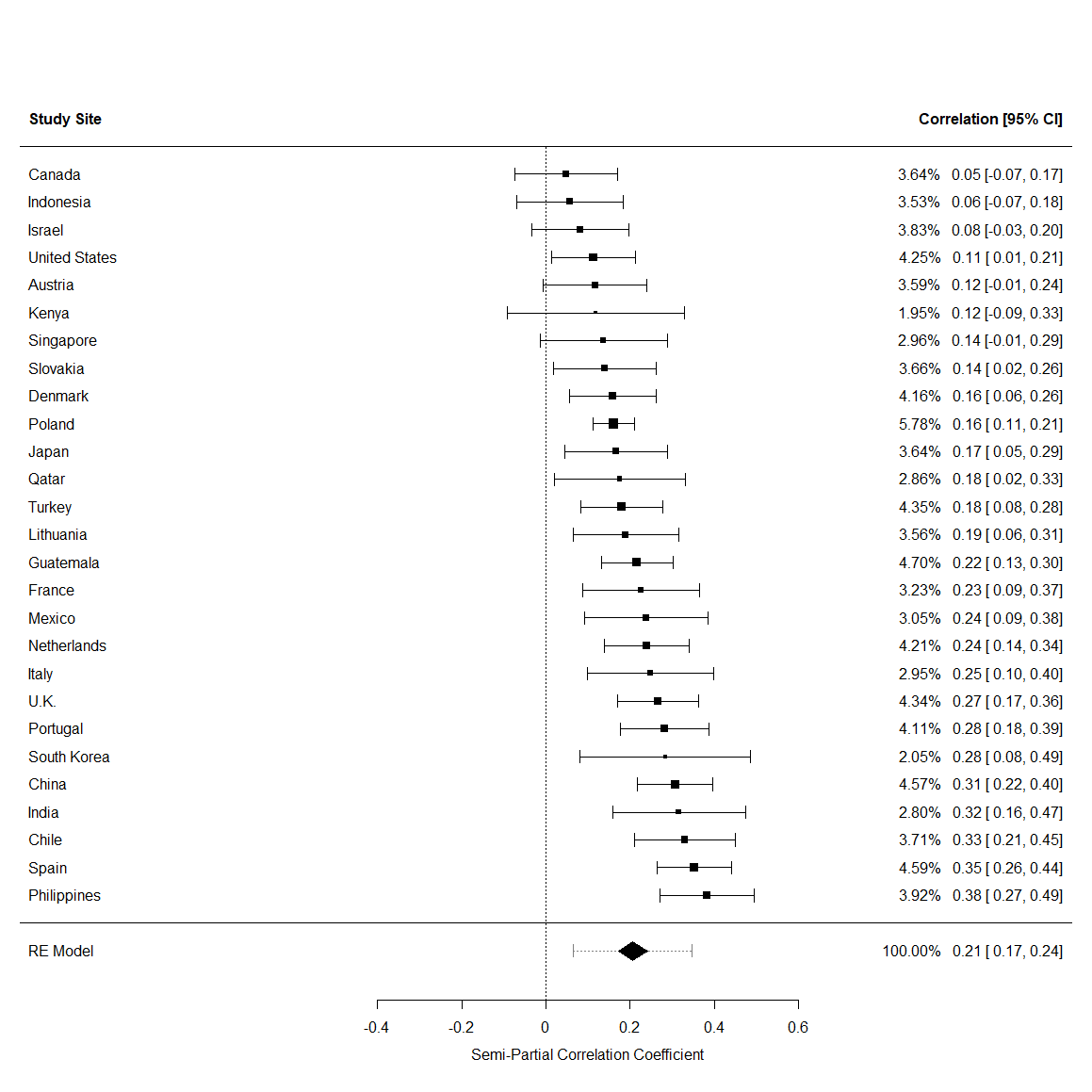


**Figure S34.** Results of random-effects meta-analysis examining the *semi-partial correlation* between the traditionalism factor scores and the COVID-19 health precautions factor scores after adjusting for the effects of the five suppressor variables. Compare to Figure 3 in the main text.

### Suppressor variable descriptives by country

In order to understand the distribution of attitudes measured by the suppressor variables (trust in scientists, social dominance orientation, and the perception of tradeoffs between COVID-19 precautions on the one hand, and personal liberties, the economy, and one’s traditions respectively on the other), we assessed mean response levels of those variables across the countries in the sample using random-effects meta-analyses of those means. Results indicate variability across nations in mean responses along the five suppressor variables (see figures S37-S41).

*Trust in scientists:*


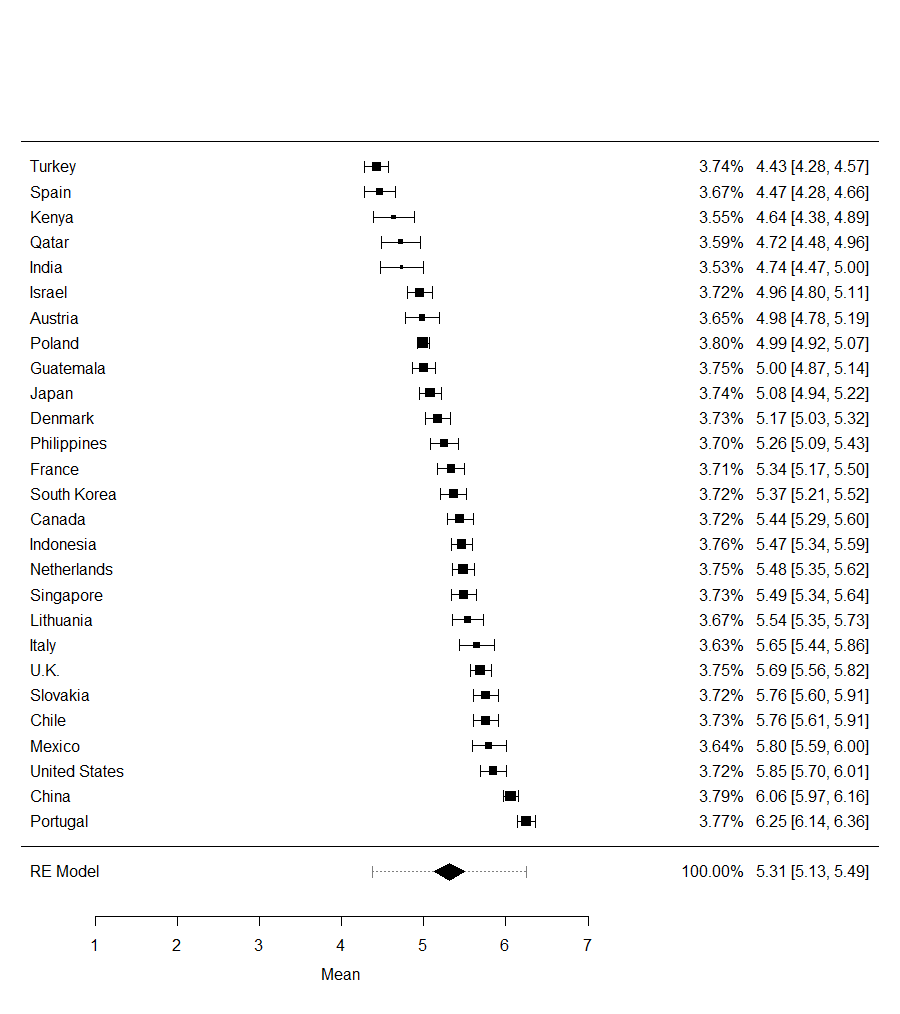


**Figure S35.** Random-effects meta-analysis of mean level of trust in scientists at each study site. Trust in scientists was measured along a 1-7 scale. Refer to Figure 1 for details on interpreting forest plots.

Note that samples were not nationally representative, and that sampling procedures differed across study sites, and thus that it would not be appropriate to draw strong inferences about differences between societies on the dimension measured here.


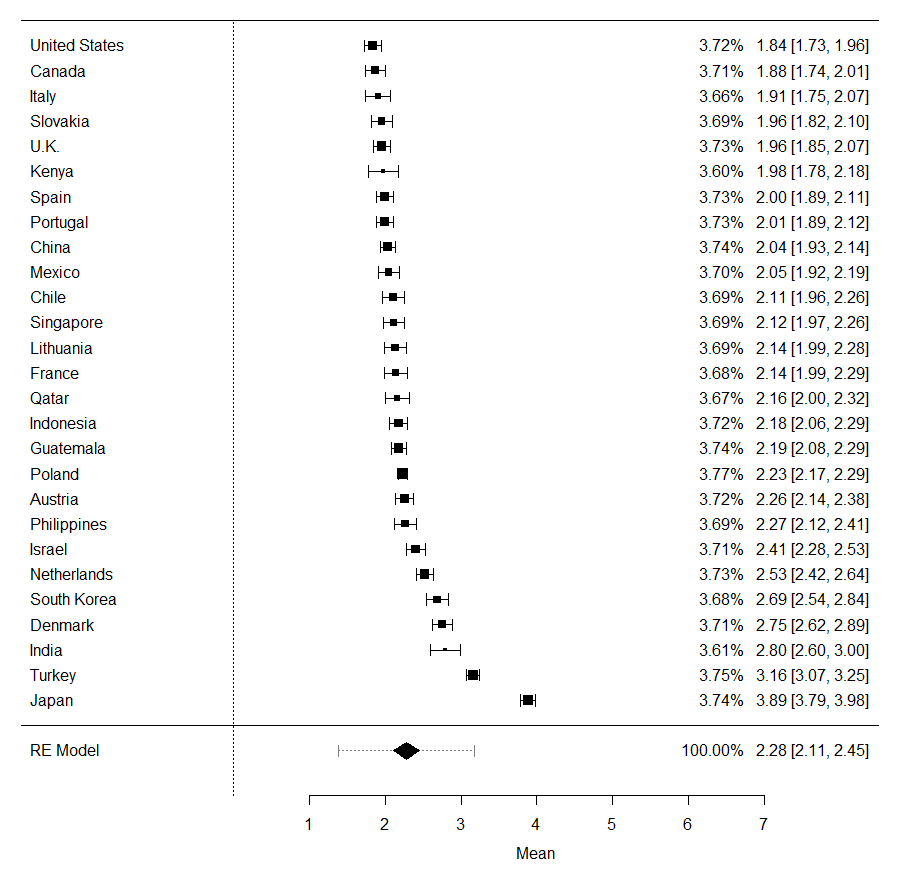
*Social dominance orientation:*

**Figure S36.** Random-effects meta-analysis of mean level of social dominance orientation at each study site. SDO was measured along a 1-7 scale. Refer to Figure 1 for details on interpreting forest plots.

Note that samples were not nationally representative, and that sampling procedures differed across study sites, and thus that it would not be appropriate to draw strong inferences about differences between societies on the dimension measured here.

*Perceived tradeoff between COVID-19 precautions and personal liberties:*


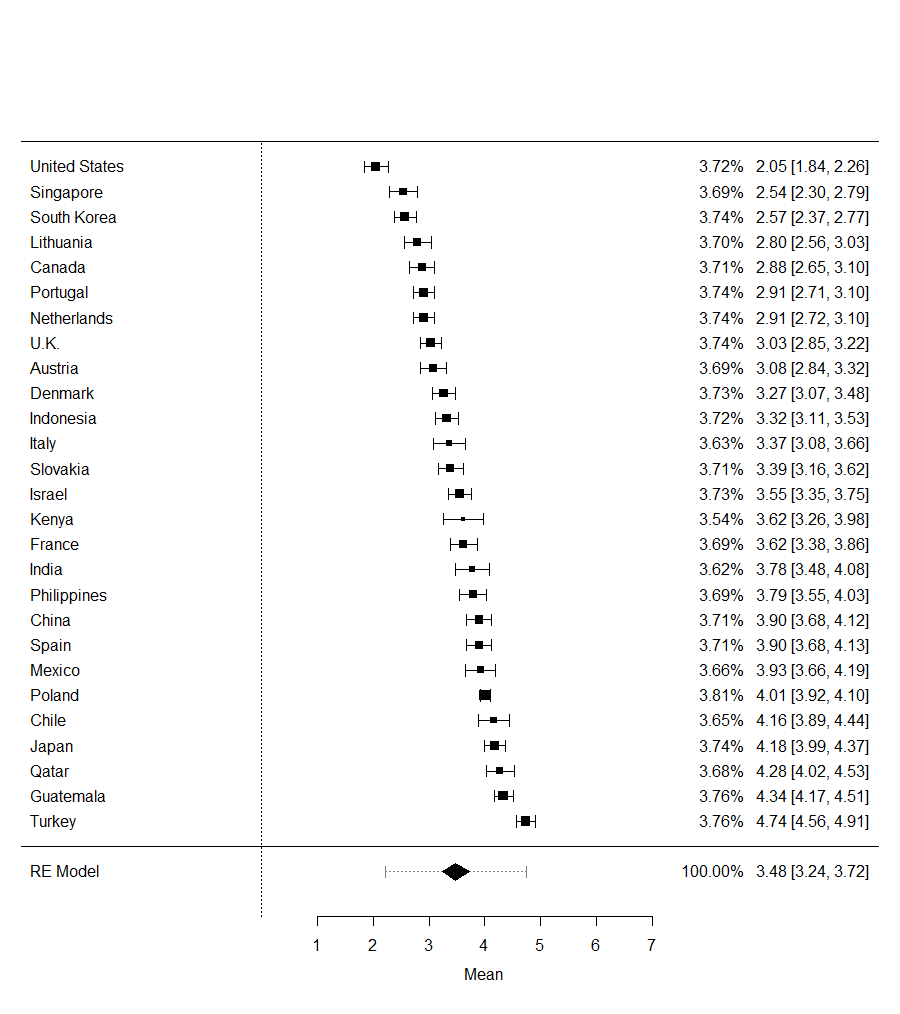


**Figure S37.** Random-effects meta-analysis of mean level of perceived tradeoffs between COVID-19 precautions and personal liberties at each study site. Tradeoffs were measured along a 1-7 scale. Refer to Figure 1 for details on interpreting forest plots.

Note that samples were not nationally representative, and that sampling procedures differed across study sites, and thus that it would not be appropriate to draw strong inferences about differences between societies on the dimension measured here.

*Perceived tradeoff between COVID-19 precautions and the economy:*


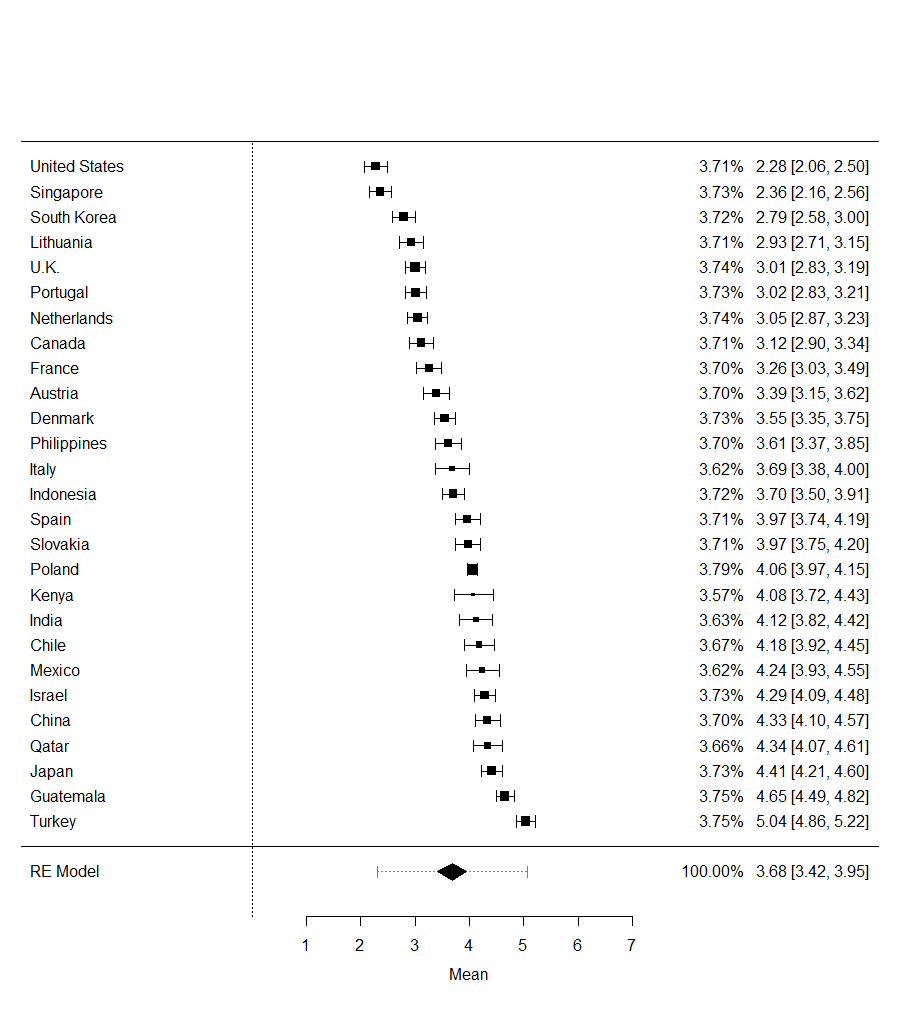


**Figure S38.** Random-effects meta-analysis of mean level of perceived tradeoffs between COVID-19 precautions and the economy at each study site. Tradeoffs were measured along a 1-7 scale. Refer to Figure 1 for details on interpreting forest plots.

Note that samples were not nationally representative, and that sampling procedures differed across study sites, and thus that it would not be appropriate to draw strong inferences about differences between societies on the dimension measured here.

*Perceived tradeoff between COVID-19 p*
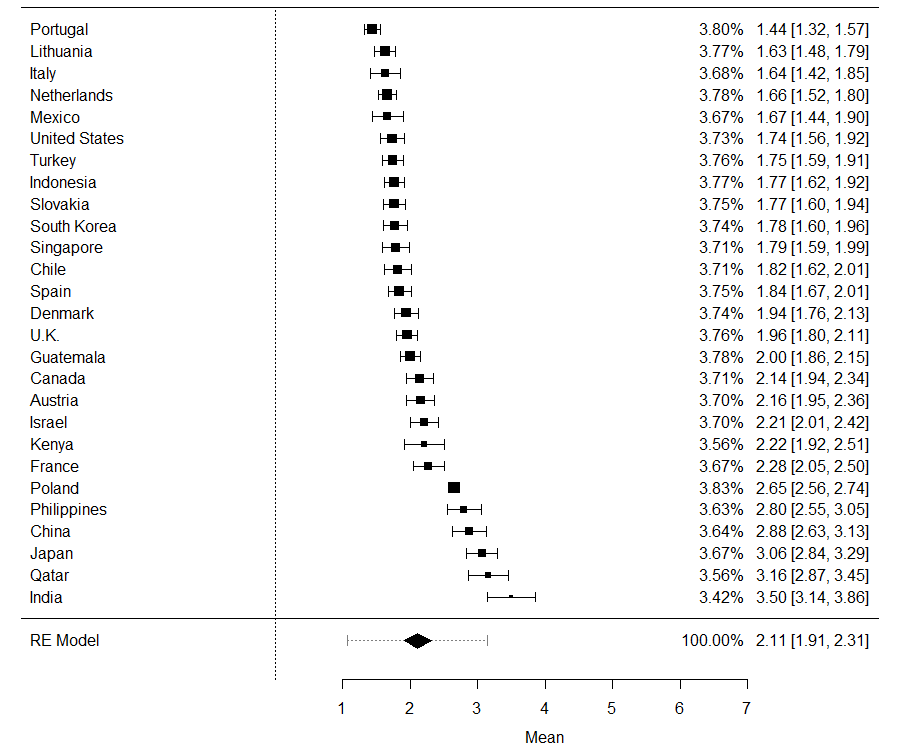
*recautions and one’s traditions:*

**Figure S39.** Random-effects meta-analysis of mean level of perceived tradeoffs between COVID-19 precautions and one’s traditions at each study site. Tradeoffs were measured along a 1-7 scale. Refer to Figure 1 for details on interpreting forest plots.

Note that samples were not nationally representative, and that sampling procedures differed across study sites, and thus that it would not be appropriate to draw strong inferences about differences between societies on the dimension measured here.

## Additional Analyses

### COVID-19 precautions and gender

Given the literature on sex differences and disgust ^see 39 for an overview^, we assessed whether self-reported COVID-19 precautions differed as a function of gender (participants were asked about their gender, not their sex assigned at birth, however the two are likely to strongly correlated in our sample). First, we visualized differences in mean COVID-19 precautions between women and men across all 27 countries in the sample (Figure S35). Then, we meta-analyzed the mean precautions difference between women and men (see Figure S36). Overall, pooling across all countries, women report taking more precautions than men on average (see overall meta-analyzed estimate in Figure S36). However, the magnitude of the difference varies across countries (range = -.02-.88; I^2^ = .39; 95% prediction intervals = .10-.55), and the difference is statistically significant in 14 of 27 countries. This suggests that while women may have tended to take more COVID-19 precautions than men overall, the precise pattern varies across nations.


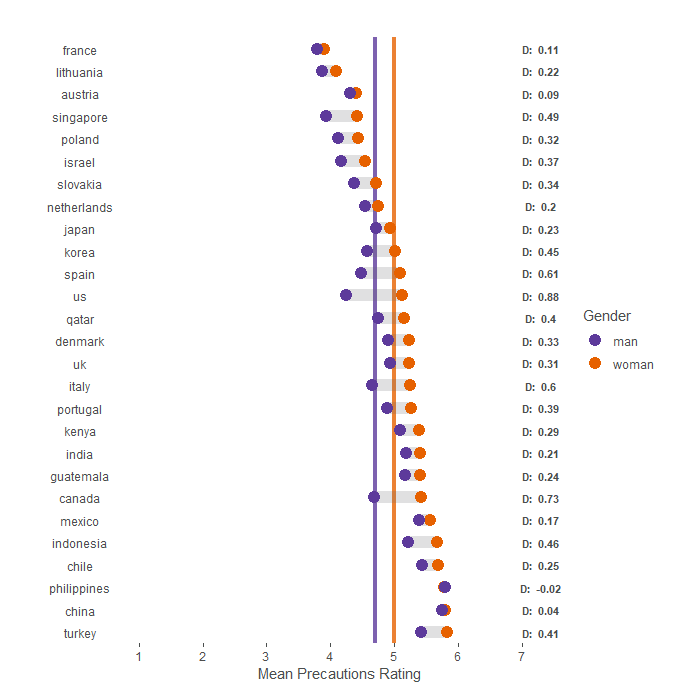


**Figure S40.** Plot of gender differences in COVID-19 precautions by country. Countries are along the y-axis, the mean precautions rating on a scale from 1-7 on the x-axis. The vertical lines represent the unweighted average precautions rating pooling across all countries. The dots represent the average precautions rating for women and men respectively for each study site, while the grey bars illustrate the distance between those two means. Along the right-hand column, the difference along the 1-7 scale between the women and men means are displayed numerically.


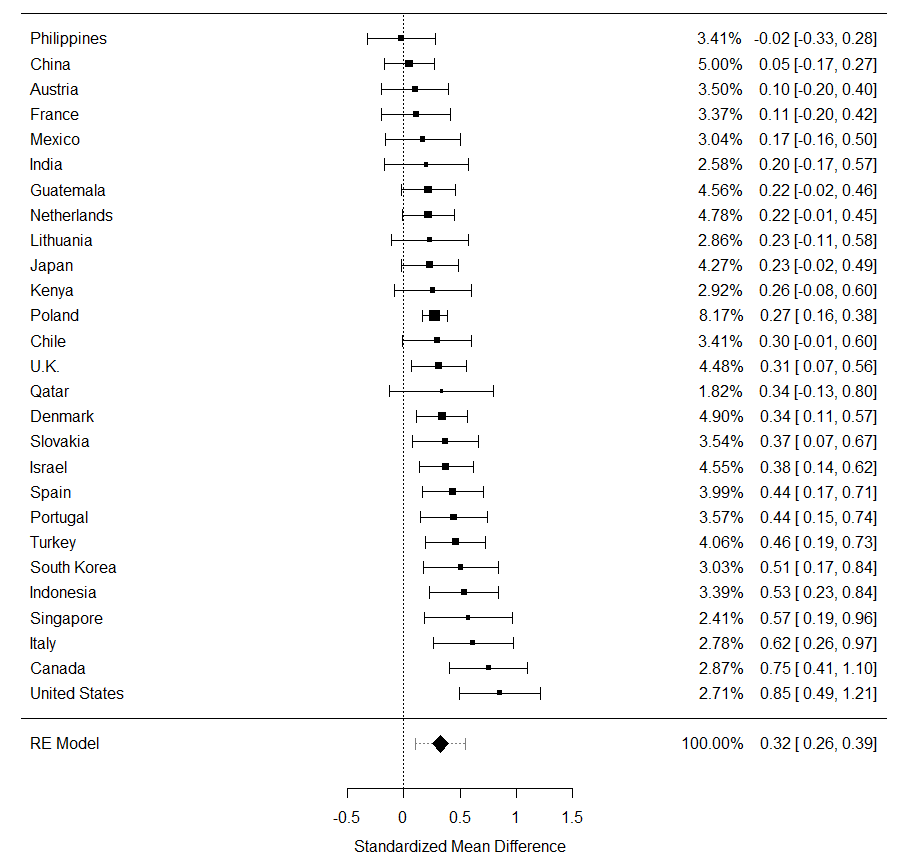


**Figure S41.** Meta-analysis of the standardized mean difference (accounting for heteroscedastic population variances^40^) in COVID-19 precautions between women and men by country. Refer to Figure 1 for details on interpreting forest plots.

##

## References

1. Dunwoody, P. & Funke, F. The aggression-submission-conventionalism scale: Testing a new three factor measure of authoritarianism. *J. Soc. Polit. Psychol.* **4**, 571–600 (2016).

2. Graham, J., Haidt, J. & Nosek, B. A. Questionnaires | Moral Foundations Theory. https://moralfoundations.org/questionnaires/ (2008).

3. Pratto, F. *et al.* Social dominance in context and in individuals: Contextual moderation of robust effects of social dominance orientation in 15 languages and 20 countries. *Soc. Psychol. Personal. Sci.* **4**, 587–599 (2013).

4. Tybur, J., Lieberman, D. & Griskevicius, V. Microbes, mating, and morality: Individual differences in three functional domains of disgust. *J. Pers. Soc. Psychol.* **97**, 103–22 (2009).

5. Navarrete, C. D. Death Concerns and Other Adaptive Challenges: The Effects of Coalition-Relevant Challenges on Worldview Defense in the US and Costa Rica. *Group Process. Intergroup Relat.* **8**, 411–427 (2005).

6. R Core Team. *R: A Language and Environment for Statistical Computing*. (R Foundation for Statistical Computing, 2020).

7. RStudio Team. *RStudio: Integrated Development Environment for R*. (RStudio, Inc., 2019).

8. Wickham, H., Hester, J., Chang, W. & Bryan, J. *devtools: Tools to Make Developing R Packages Easier*. (2021).

9. Wickham, H. *ggplot2: Elegant Graphics for Data Analysis*. (Springer-Verlag New York, 2016).

10. Auguie, B. gridExtra: Miscellaneous Functions for ‘Grid’ Graphics. (2017).

11. Long, J. A. *interactions: Comprehensive, User-Friendly Toolkit for Probing Interactions*. (2019).

12. Zhu, H. *kableExtra: Construct Complex Table with ‘kable’ and Pipe Syntax*. (2021).

13. Rosseel, Y. lavaan: An R Package for Structural Equation Modeling. *J. Stat. Softw.* **48**, 1–36 (2012).

14. Bates, D., Mächler, M., Bolker, B. & Walker, S. Fitting linear mixed-effects models using lme4. *J. Stat. Softw.* **67**, 1–48 (2015).

15. Kuznetsova, A., Brockhoff, P. B. & Christensen, R. H. B. lmerTest Package: Tests in Linear Mixed Effects Models. *J. Stat. Softw.* **82**, 1–26 (2017).

16. Venables, W. N. & Ripley, B. D. *Modern Applied Statistics with S*. (Springer, 2002).

17. Bates, D. & Maechler, M. *Matrix: Sparse and Dense Matrix Classes and Methods*. (2021).

18. Tingley, D., Yamamoto, T., Hirose, K., Keele, L. & Imai, K. mediation: R package for causal mediation analysis. *J. Stat. Softw.* **59**, 1–38 (2014).

19. Viechtbauer, W. Conducting meta-analyses in R with the metafor package. *J. Stat. Softw.* **36**, 1–48 (2010).

20. Genz, A. & Bretz, F. *Computation of Multivariate Normal and t Probabilities*. (Springer-Verlag, 2009).

21. Genz, A. *et al.* *mvtnorm: Multivariate Normal and t Distributions*. (2021).

22. Lüdecke, D., Ben-Shachar, M. S., Patil, I. & Makowski, D. Extracting, Computing and Exploring the Parameters of Statistical Models using R. *J. Open Source Softw.* **5**, 2445 (2020).

23. Revelle, W. psych: Procedures for Psychological, Psychometric, and Personality Research. (2019).

24. Makowski, D., Ben-Shachar, M. S., Patil, I. & Lüdecke, D. Automated Results Reporting as a Practical Tool to Improve Reproducibility and Methodological Best Practices Adoption. *CRAN* (2021).

25. Zeileis, A. Object-Oriented Computation of Sandwich Estimators. *J. Stat. Softw.* **16**, 1–16 (2006).

26. Zeileis, A., Köll, S. & Graham, N. Various Versatile Variances: An Object-Oriented Implementation of Clustered Covariances in R. *J. Stat. Softw.* **95**, 1–36 (2020).

27. Wickham, H. & Seidel, D. *scales: Scale functions for visualization*. (2020).

28. Lüdecke, D. *sjPlot: Data Visualization for Statistics in Social Science*. (2020).

29. Wickham, H. *et al.* Welcome to the tidyverse. *J. Open Source Softw.* **4**, 1686 (2019).

30. Ritchie, H. *et al.* Coronavirus pandemic (COVID-19). *Our World Data* (2020).

31. Samore, T., Fessler, D. M. T., Sparks, A. M. & Holbrook, C. Of Pathogens and Party Lines: Social Conservatism Positively Associates with COVID-19 Precautions among U.S. Democrats but not Republicans. Preprint at https://doi.org/10.31234/osf.io/9zsvb (2020).

32. Graham, J. *et al.* Mapping the moral domain. *J. Pers. Soc. Psychol.* **101**, 366–385 (2011).

33. Doğruyol, B., Alper, S. & Yilmaz, O. The five-factor model of the moral foundations theory is stable across WEIRD and non-WEIRD cultures. *Personal. Individ. Differ.* **151**, 109547 (2019).

34. Iurino, K. & Saucier, G. Testing Measurement Invariance of the Moral Foundations Questionnaire Across 27 Countries. *Assessment* **27**, 365–372 (2020).

35. Tybur, J. M. *et al.* Parasite stress and pathogen avoidance relate to distinct dimensions of political ideology across 30 nations. *Proc. Natl. Acad. Sci.* **113**, 12408–12413 (2016).

36. Suárez-Álvarez, J., Pedrosa, I. & Lozano, L. M. Using reversed items in Likert scales: A questionable practice. *Psicothema* 149–158 (2018) doi:10.7334/psicothema2018.33.

37. Swain, S. D., Weathers, D. & Niedrich, R. W. Assessing Three Sources of Misresponse to Reversed Likert Items. *J. Mark. Res.* **45**, 116–131 (2008).

38. Wong, N., Rindfleisch, A. & Burroughs, J. Do Reverse-Worded Items Confound Measures in Cross-Cultural Consumer Research? The Case of Material Value Scale. *J. Consum. Res.* **30**, 72–91 (2003).

39. Sparks, A. M., Fessler, D. M. T., Chan, K. Q., Ashokkumar, A. & Holbrook, C. Disgust as a mechanism for decision making under risk: Illuminating sex differences and individual risk-taking correlates of disgust propensity. *Emot. Wash. DC* **18**, 942–958 (2018).

40. Bonett, D. G. Meta-analytic interval estimation for standardized and unstandardized mean differences. *Psychol. Methods* **14**, 225–238 (2009).
